# Pentoxifylline, Inflammation, and Endothelial Function in HIV-Infected Persons: A Randomized, Placebo-Controlled Trial

**DOI:** 10.1371/journal.pone.0060852

**Published:** 2013-04-09

**Authors:** Samir K. Gupta, Deming Mi, Michael P. Dubé, Chandan K. Saha, Raymond M. Johnson, James H. Stein, Matthias A. Clauss, Kieren J. Mather, Zeruesenay Desta, Ziyue Liu

**Affiliations:** 1 Division of Infectious Diseases, Department of Medicine, Indiana University School of Medicine, Indianapolis, Indiana, United States of America; 2 Department of Biostatistics, Indiana University School of Medicine, Indianapolis, Indiana, United States of America; 3 Division of Infectious Diseases, Department of Medicine, University of Southern California Keck School of Medicine, Los Angeles, California, United States of America; 4 Division of Cardiovascular Medicine, Department of Medicine, University of Wisconsin School of Medicine and Public Health, Madison, Wisconsin, United States of America; 5 Department of Cellular & Integrative Physiology, Center for Vascular Biology, Indiana University School of Medicine, Indianapolis, Indiana, United States of America; 6 Division of Endocrinology & Metabolism, Department of Medicine, Indiana University School of Medicine, Indianapolis, Indiana, United States of America; 7 Division of Clinical Pharmacology, Department of Medicine, Indiana University School of Medicine, Indianapolis, Indiana, United States of America; University of Nebraska Medical Center, United States of America

## Abstract

**Background:**

Untreated HIV may increase the risk of cardiovascular events. Our preliminary *in vitro* and *in vivo* research suggests that pentoxifylline (PTX) reduces vascular inflammation and improves endothelial function in HIV-infected persons not requiring antiretroviral therapy.

**Methods:**

We performed a randomized, placebo-controlled trial of PTX 400 mg orally thrice daily for 8 weeks in 26 participants. The primary endpoint was change in flow-mediated dilation (FMD) of the brachial artery after 8 weeks. Nitroglycerin-mediated dilation (NTGMD) and circulating markers of inflammation, cellular immune activation, coagulation, and metabolism were also assessed.

**Results:**

The difference in mean absolute change (SD) in FMD after 8 weeks between the placebo [−1.06 (1.45)%] and PTX [−1.93 (3.03)%] groups was not significant (P = 0.44). No differences in NTGMD were observed. The only significant between-group difference in the changes in biomarkers from baseline to week 8 was in soluble tumor necrosis factor receptor-1 (sTNFRI) [−83.2 pg/mL in the placebo group vs. +65.9 pg/mL in the PTX group; P = 0.03]. PTX was generally well-tolerated.

**Conclusions:**

PTX did not improve endothelial function and unexpectedly increased the inflammatory biomarker sTNFRI in HIV-infected participants not requiring antiretroviral therapy. Additional interventional research is needed to reduce inflammation and cardiovascular risk in this population.

**Trial Registration:**

ClinicalTrials.gov NCT00796822

## Introduction

HIV-infected patients are at an increased risk for developing cardiovascular disease (CVD) compared to the uninfected population [1], [2]. Much of this increased risk had previously been attributed to the dysmetabolic effects associated with the use of antiretroviral therapy (ART) [3]. However, recent data strongly suggest that HIV-related systemic inflammation also contributes to this excess CVD risk as well as overall mortality, especially in those not receiving ART [4], [5], [6], [7].

Endothelial dysfunction is a key and initial promoter of atherosclerosis in the general population [8], [9]. Because systemic inflammation is associated with endothelial dysfunction [10], reduction of inflammation may improve endothelial health and consequently reduce the risk of future CVD events [11], [12].

We have previously shown that pentoxifylline (PTX), a phosphodiesterase inhibitor, can downregulate the endothelial activation marker vascular cell adhesion molecule-1 (VCAM-1) in an *in vitro* HIV-1 endothelial cell model [13]. In addition, we demonstrated in an open-label, single arm, 8 week pilot trial of PTX given to HIV-infected patients not requiring ART that PTX use was well-tolerated and reduced circulating levels of sVCAM-1 and interferon-γ-induced protein 10 (IP-10). Moreover, flow-mediated dilation (FMD) of the brachial artery [14], a validated measure of *in vivo* endothelial function and predictor of future cardiovascular events [11], [12], improved significantly. Taken together, these data suggest that PTX may inhibit leukocyte adhesion pathways that are involved in vascular inflammation and dysfunction in those with HIV infection. If so, then PTX would potentially be an inexpensive, safe, and readily available therapy to reduce systemic inflammation and improve the cardiovascular risk profile in the HIV-infected population. On the basis of these initial observations, we conducted a randomized, double-blind, placebo-controlled trial of PTX in HIV-infected participants not on ART to test the hypothesis that PTX would reduce systemic inflammation and improve FMD in this population.

## Methods

### Study Design

The protocol and informed consent form for this trial and supporting CONSORT checklist are available as supporting information; see Protocol and Consent S1 and Checklist S1.

We performed a randomized, double-blind, placebo-controlled, parallel group trial of oral PTX 400 mg given thrice daily for 8 weeks in HIV-infected study participants not requiring ART per DHHS Guidelines at the time of the study (ClinicalTrials.gov NCT00796822). We excluded those on ART in order to isolate the effects of PTX on untreated HIV and to avoid the potentially confounding effects of ART on endothelial function [15], [16] and to assess why lack of ART may predispose to endothelial dysfunction. Participants had study procedures performed at baseline, 4 weeks, and 8 weeks. Participants underwent assessment for eligibility at a screening visit and within 21 days were then randomized 1∶1 via a computerized random-generated list (with a block size of 4 for the first 24 participants and a block size of 2 for the remaining 2 participants) to either PTX or matching placebo. PTX was given as 400 mg extended-release tablets purchased commercially. Both PTX and placebo were over-encapsulated with gelatin capsules with cellulose backfill to provide matching color, taste, size, smell, and texture. Adherence was assessed by 3-day recall of missed doses at each study visit. Adverse events were assessed at each visit and in between scheduled visits as needed. Assessment of successful blinding was performed at the end of trial participation by asking the participants if they thought they knew to which study arm they were assigned.

The protocol underwent one major amendment and revision in February 2009 to clarify eligibility criteria. Over the course of the trial, there were 8 protocol deviations (5 for out of window study visits to accommodate participant schedules, 2 for out of window measurements of HIV-1 RNA levels due to faulty lab equipment, 1 for not having advanced flow cytometry measurements performed at one study visit).

### Study Population

Participants were recruited from the HIV outpatient clinics associated with the Indiana University Health medical system. Primary inclusion criteria included documented HIV-1 infection, age ≥18 years, CD4 cell count ≥350/µL at screening, no receipt of ART within 6 months of screening and no anticipated need for ART during the course of trial participation. Major exclusion criteria included diagnosed cardiovascular disease, diabetes, hypertension, thyroid abnormalities, other systemic inflammatory disease (although hepatitis B or C co-infection was allowed); pregnancy or breastfeeding during the trial; known intolerance to PTX or other methylxanthines; creatinine clearance <50 mL/min, hemoglobin <9.0 g/dL, alanine (ALT) or aspartate (AST) aminotransferase >3 times upper limit of normal, total bilirubin >2.5 times upper limit of normal; ongoing fever or active infection/malignancy during a study visit; or use of anti-inflammatory (including aspirin or non-steroidal anti-inflammatory drugs), lipid-lowering, or anticoagulation agent at screening or during the trial.

### Study Procedures

Participants were required to fast and not smoke for at least 8 hours prior to all study procedures. FMD and nitroglycerin-mediated dilation (NTGMD) studies were performed at all study visits according to recommended guidelines [17] by a single registered vascular ultrasonographer who was certified by the University of Wisconsin Atherosclerosis Imaging Research Program Core Laboratory. After resting supine for 10-minutes in a temperature-controlled room, a blood pressure cuff was placed on the widest part of proximal right forearm approximately 1 cm distal to the antecubital fossa. Using a 10 MHz resolution linear array vascular ultrasound transducer with an Acuson CV70 ultrasound machine, the brachial artery was located above the elbow and scanned in longitudinal sections. After recording baseline B-mode digital images of the brachial artery and spectral Doppler images of flow, the forearm cuff was inflated to 250 mmHg for 5 minutes to induce reactive hyperemia. Immediately after deflation, spectral Doppler images are obtained to verify hyperemia. FMD of the brachial artery was measured 60 and 90 seconds after cuff deflation. The relative FMD (%) was calculated as the ratio between the largest post-cuff release and the baseline diameter. Fifteen minutes later, repeat brachial artery images were obtained and 400 mcg of sublingual nitroglycerin was administered. The artery was re-imaged 3 minutes later. NTGMD was calculated in an analogous fashion. Images were sent electronically to the University of Wisconsin core imaging laboratory for quality control and interpretation by a blinded, single, experienced technician using Access Point Web software (Freeland Systems, Westminster, CO).

Cellular immune activation, defined as circulating proportions of CD3+CD8+CD38+HLA-DR+ cells, was assessed by flow cytometry using fresh whole blood on the same day as the collection at baseline and at week 8. Circulating serum levels of PTX were measured using an in-house high performance liquid chromatography assay. Circulating inflammatory markers [high sensitivity C-reactive protein (hsCRP), serum interleukin-6 (IL-6), soluble tumor necrosis factor-α receptors I and II (sTNFRI, sTNFRII), tissue inhibitor of metalloproteinase-1 (TIMP-1), monocyte chemoattractant protein-1 (MCP-1), interferon-γ-induced protein 10 (IP-10)], a coagulation marker [plasminogen activating inhibitor antigen-1 (PAI-1 Ag)], an endothelial activation marker [soluble vascular cell adhesion molecule-1 (sVCAM-1)], and metabolic markers [lipid fractions, insulin, glucose] were measured in batches from archived frozen samples (kept at −80°C) at the University of Vermont Laboratory for Clinical Biochemistry Research. The homeostasis model assessment-insulin resistance (HOMA-IR) was used to estimate insulin resistance from fasting glucose and insulin measures [18]. hsCRP, IL-6, IP-10, lipids, and insulin were measured from serum, MCP-1, sTNFRI and II, sVCAM-1, and TIMP-1 were measured from EDTA plasma, and PAI-1 Ag was measured from citrated plasma. Safety laboratories were assessed at the Indiana University Health commercial laboratory at each study visit.

### Statistical Analysis

The sample size was determined based on a two-sample, independent, two-tailed t-test with 5% type I error for the primary endpoint of change in FMD from baseline to week 8. Using the results from our pilot trial [14], we conservatively estimated a predicted absolute change in FMD of 3.5% with PTX (assuming no change in placebo-treated participants) and we assumed a common standard deviation of 2.6%. A sample size of 10 per group was estimated to provide at least 80% power to detect this effect size. Allowing for a 20% dropout rate, we planned to recruit 13 subjects per group.

Continuous variables were summarized by treatment groups using descriptive statistics. Categorical variables were summarized using frequency counts and percentages. Baseline clinical and demographic data were compared between two treatment groups. Continuous variables were summarized by treatment groups using descriptive statistics. Categorical variables were summarized using frequency counts and percentages. Baseline clinical and demographic data were compared between two treatment groups. Categorical variables were examined using Fisher’s exact test. We employed Student’s t-test for comparisons of continuous measures as we found no evidence of violation of the normality assumption for these variables; of note, HIV-1 RNA level, HOMA-IR, and hsCRP required logarithmic transformation to approximate normal distributions prior to such analysis. Independent two-sample Student’s t-test was used to compare the mean changes in FMD from baseline to week 8 between the placebo and PTX groups. To account for missing week 8 FMD measures, Lachin’s worst-rank analysis approach [19] was used as part of the intent-to-treat analysis.

As we specifically wished to evaluate the effects of covariates on the primary outcomes, we also performed multiple linear regressions adjusted for treatment on the changes in FMD and NTGMD for 4 and 8 weeks. In these models, the indicator variable for PTX was kept regardless of its significance, while other potential baseline covariates, including age, sex, race, body mass index, FMD, and laboratory data, were included one at a time.

The computerized randomization list was generated by the study statistician and kept by the study pharmacist who then provided study drug or placebo in matching containers. Study participants, study personnel, and all outcome assessors were blinded to the allocation. Analyses were performed as intention to treat but without corrections for multiple testing for the secondary analyses. Two-sided P-values <0.05 were considered statistically significant. All analyses were performed in SAS 9.3 (SAS Inc., Cary, NC).

### Ethics Statement

This trial was approved by the Indiana University Institutional Review Board. All participants provided written, informed consent prior to screening.

## Results

### Study Cohort Characteristics

**Figure 1:**
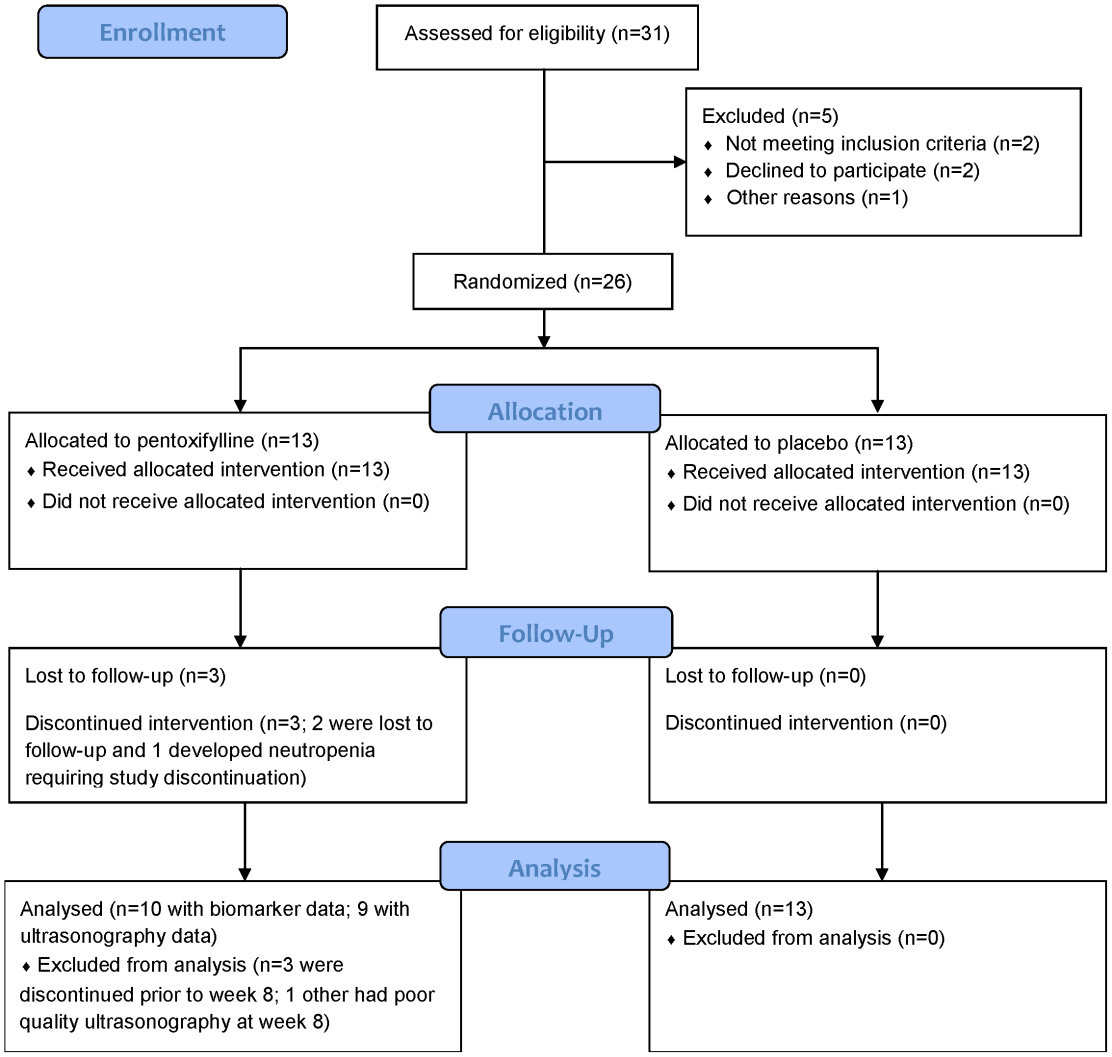
Flow of participants through the trial.

Figure 1 outlines the flow of the study participants through the trial. Thirty-one potential participants underwent screening. Study recruitment, enrollment, and follow-up assessments were performed from May 2009 through October 2011. Of these, 1 was found to be pregnant, 1 required a prohibited medication, 1 could not provide blood samples (difficult venipuncture), and 2 withdrew consent prior to randomization. The characteristics of the remaining 26 participants are shown in Table 1. The majority of participants were non-Hispanic, non-smoking, black men. Of note, no white women enrolled into the trial. The mean (standard deviation, SD) CD4 cell count and HIV-1 RNA level for the entire study group was 555 (169)/µL and 4.0 (0.9) log_10_copies/mL, respectively. None had active hepatitis B or C co-infection. There were no significant differences in the baseline characteristics between arms. All 13 participants in the placebo arm completed the 8 week trial. However, 2 of the PTX participants were lost to follow-up by week 4, 1 developed Grade 2 neutropenia at week 4 and was subsequently withdrawn, and 1 had poor ultrasound data quality at week 8. Thus, 11 and 9 participants, respectively, of the 13 initial PTX participants had evaluable vascular imaging data at the weeks 4 and 8 study visits; 11 and 10, respectively, in the PTX group and samples available for biomarker analysis.

**Table 1:** Baseline Characteristics of the Trial Participants (N = 26).

Characteristic	Placebo (N = 13)	Pentoxifylline (N = 13)
Age, years	34 (10.9)	40 (11.6)
Male	11 (85%)	8 (62%)
Black race	8 (62%)	8 (62%)
Hispanic ethnicity	1 (8%)	0 (0%)
Current smoker	5 (38%)	6 (46%)
Body mass index, kg/m^2^	27.7 (5.9)	26.1 (4.6)
CD4 cell count/µL	583 (175)	524 (165)
HIV–1 RNA level, log_10_copies/mL	4.0 (1.2)	4.0 (0.7)

Notes: Data presented as means (standard deviations) or as No. (%). No statistically significant differences were found between these baseline characteristics.

At the 4 week visit, 15 of the 24 evaluable study participants, respectively, claimed no missed study drug and the remainder claimed no more than 3 missed doses in the 3 days prior to the study visit. At week 8, 12 of the evaluable 22 participants claimed no missed doses and 7 of the remaining 10 claimed no more than 3 missed doses in the 3 days prior to the study visit. At weeks 4 and 8, 3 PTX participants at each time point had no measurable PTX drug concentration; 2 of the 3 PTX participants had no measurable drug concentration at both weeks 4 and 8.

Because one participant in the PTX group was removed due to an adverse event, unblinding of this participant’s randomization assignment may have occurred. To assess potential selection bias after withdrawal of this participant, the Berger-Exner test [20] was performed and was found to have a P-value of 0.11, suggesting no significant selection bias.

### Changes in Vascular Measures

**Figure 2:**
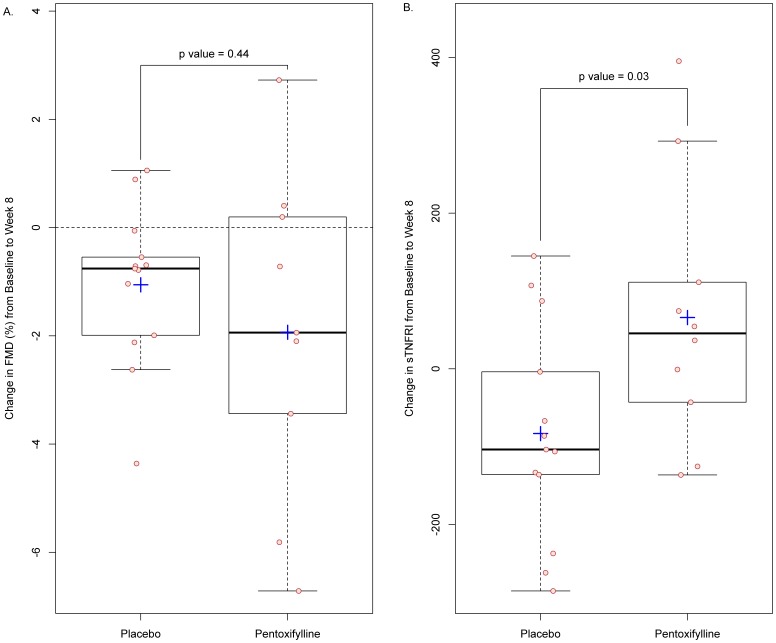
Changes in flow-mediated dilation (FMD) of the brachial artery and soluble tumor necrosis factor receptor-1 (sTNFRi) from baseline to week 8. Panel A shows the changes in FMD; Panel B shows the changes in sTNFRI. Circles indicate actual values. Top and bottom of boxes indicate 75^th^ and 25^th^ percentiles, respectively. Internal horizontal lines indicate median values and plus-signs indicate mean values. External horizontal lines/whiskers indicate 25^th^ or 75^th^ percentiles ± (1.5 times interquartile range).

The absolute mean values of the primary outcome measure, FMD, and other vascular measures at each time point are shown in Table 2. FMD generally declined in both treatment groups (Figure 2A). The mean (SD) difference in absolute change in FMD at 8 weeks between the placebo [−1.06 (1.45)%] and PTX [−1.93 (3.03)%] groups was not significant (P = 0.44). Using Lachin’s worst-rank analysis approach to account for missing week 8 FMD measurements, the difference in change in FMD from baseline to week 8 was again not significant (P = 0.08).

**Table 2:** Vascular Measures during the Trial.

Characteristic	Placebo	Pentoxifylline	P-value^1^
**Resting brachial artery** **diameter, cm**			
Baseline	0.44 (0.05)	0.42 (0.08)	0.60
Week 4	0.44 (0.05)	0.43 (0.08)	0.72
Week 8	0.44 (0.04)	0.43 (0.07)	0.68
**Flow-mediated dilation (%)**			
Baseline	3.46 (2.32)	4.09 (2.87)	0.54
Week 4	3.36 (2.78)	2.35 (1.53)	0.27
Week 8	2.40 (1.58)^2^	2.80 (1.69)^2^	0.58
**Nitroglycerin-mediated** **dilation (%)**			
Baseline	13.66 (7.02)	15.88 (6.59)	0.42
Week 4	16.68 (7.11)	13.91 (6.51)	0.34
Week 8	15.28 (5.76)	17.16 (8.16)	0.57

Notes: Data presented as means (standard deviation). All 13 placebo subjects had vascular imaging data available at all study visits. Of the 13 pentoxifylline subjects, 11 and 9 had vascular imaging data available at weeks 4 and 8 respectively.

1 For comparisons between placebo and pentoxifylline groups.

2 The reduction in FMD from baseline to week 8 was statistically significant within the placebo group (P = 0.02) but not within the pentoxifylline group (P = 0.09). No significant changes were found within groups in any other vascular measure from baseline to week 8 or from baseline to week 4.

The within-group changes in FMD from baseline through week 8 was statistically significant in the placebo group (P = 0.02) but not within the PTX group (P = 0.09); no significant changes were found within groups from baseline through week 4. In models that explored possible predictors of change in FMD after 8 weeks and PTX treatment, both sex (P = 0.003) and week 0 FMD (P<0.0001) were identified; men had significantly less of a decrease in FMD compared to women and those with greater FMD at baseline had greater decreases in FMD. Of note, smoking status was not associated with FMD change.

**Figure 3:**
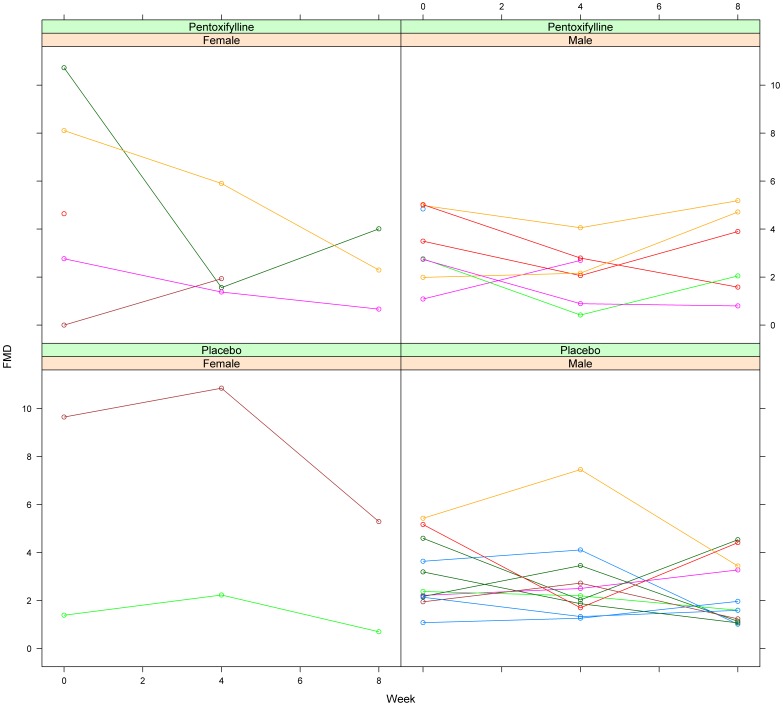
Individual changes in FMD by treatment group (pentoxifylline vs. placebo) and by sex (female vs. male).

A post-hoc exploratory analysis of differences in FMD by sex and baseline FMD is shown in Figure 3. As expected, women had higher FMD at baseline (3 of 7 women vs. 0 of 19 men had baseline FMD >6.0%); these 3 women had large decreases in FMD during the trial. As shown in Table 3, brachial artery diameters at baseline were, as expected, significantly larger in men compared to women, but this did not translate into a significant difference in mean baseline FMD. Baseline brachial artery diameters did not change over time in either men or women. We then examined a model including PTX, sex, and week 0 FMD on changes in FMD after 8 weeks; week 0 FMD remained significantly associated (P = 0.0009) and sex was marginally associated (P = 0.09) with change in FMD after 8 weeks.

**Table 3:** Vascular Measures during the Trial by Sex.

Characteristic	Men	Women	P-value^1^
**Resting brachial artery** **diameter, cm**			
Baseline	0.45 (19; 0.05)	0.38 (7; 0.06)	0.04
Week 4	0.45 (18; 0.05)	0.37 (6; 0.06)	0.02
Week 8	0.44 (17; 0.04)	0.39 (5; 0.07)	0.15
**Flow-mediated** **dilation (%)**			
Baseline	3.20 (19; 1.42)	5.33 (7; 4.21)	0.24
Week 4	2.54 (18; 1.57)	3.98 (6; 3.76)	0.40
Week 8	2.56 (17; 1.52)	2.59 (5; 2.04)	0.97

Notes: Data presented as means (N; standard deviations). All 13 placebo subjects had vascular imaging data available at all study visits. Of the 13 pentoxifylline subjects, 11 and 9 had vascular imaging data available at weeks 4 and 8 respectively.

1 For comparisons between men and women.

There was also no significant difference (P = 0.14) in mean absolute change (SD) in FMD at week 4 between the placebo [−0.10 (1.58)%] and PTX [−1.62 (2.92)%] groups. In models including all other variables and PTX assignment, no significant effects of these other variables on FMD were found.

In analyses including only those PTX participants who had detectable PTX drug concentrations, no significant differences in the changes in FMD at either 4 or 8 weeks between groups were observed (data not shown). There were no significant differences in change in FMD at week 8 in those who did have and did not have detectable PTX levels (data not shown).

There were no significant changes in NTGMD at either week 4 or week 8 between the two groups.

### Changes in Biomarkers

The absolute mean values of the immunologic, virologic, inflammatory, coagulation, and metabolic parameters assessed during the trial are presented in Table 4. PAI-1 Ag was significantly higher in the PTX group at both baseline and at week 8. However, there were no significant differences in the changes in PAI-1 Ag between groups. As shown in Figure 2B, the only significant difference in the changes in these biomarkers from baseline to week 8 was in sTNFRI [−83.2 pg/mL in the placebo group vs. 65.9 pg/mL in the PTX group; P = 0.03]. This change in sTNFRI was not significantly correlated with the change in FMD. Of note, there were no significant within-group changes in any biomarker through week 8.

**Table 4:** Immunologic, Virologic, Inflammatory, Coagulation, Metabolic, and Pentoxifylline Concentration Levels during the Trial.

Characteristic	Placebo	Pentoxifylline	P-value^1^
**CD4 cell count/µL**			
Baseline	583 (175)	524 (165)	0.39
Week 4	534 (206)	601 (269)	0.52
Week 8	526 (165)	494 (208)	0.72
**CD3+CD8+CD38+HLA-DR+proportions (%)**			
Baseline	36 (19)	39 (15)	0.69
Week 8	36 (19)	41 (17)	0.52
**HIV-1 RNA, log10copies/mL**			
Baseline	4.0 (1.2)	4.0 (0.7)	0.88
Week 4	3.9 (1.1)	4.0 (4.5)	0.72
Week 8	4.0 (1.0)	4.0 (0.8)	0.87
**hsCRP, log_10_mg/L**			
Baseline	0.27 (0.41)	0.25 (0.57)	0.95
Week 4	0.36 (0.57)	0.37 (0.80)	0.74
Week 8	−0.01 (0.45)	0.15 (0.81)	0.58
**IL-6, pg/ml**			
Baseline	1.91 (1.53)	2.61 (2.59)	0.41
Week 4	3.44 (6.52)	2.91 (2.45)	0.79
Week 8	1.7 (1.55)	5.26 (10.2)	0.30
**TIMP-1, ng/ml**			
Baseline	99 (17)	117 (35)	0.12
Week 4	98 (20)	112 (31)	0.21
Week 8	91 (14)	109 (25)	0.06
**sVCAM-1, ng/mL**			
Baseline	1037 (399)	1108 (273)	0.60
Week 4	1081 (419)	1156 (283)	0.6
Week 8	1098 (462)	1130 (281)	0.84
**sTNFRI, pg/mL**			
Baseline	1125 (249)	851 (379)	0.04
Week 4	1047 (244)	805 (298)	0.04
Week 8	1042 (205)	805 (298)	0.12
**sTNFRII, pg/mL**			
Baseline	7239 (2606)	7736 (2141)	0.60
Week 4	7163 (2106)	7762 (2562)	0.54
Week 8	7455 (2405)	7549 (1945)	0.92
**MCP-1, pg/mL**			
Baseline	237 (75)	202 (76)	0.24
Week 4	193 (55)	205 (100)	0.71
Week 8	217 (55)	207 (106)	0.80
**IP-10, pg/mL**			
Baseline	432 (330)	552 (331)	0.36
Week 4	386 (283)	528 (324)	0.27
Week 8	377 (295)	440 (182)	0.53
**PAI-1 Ag, ng/ml**			
Baseline	17.3 (6.9)	43.3 (27.5)	0.01
Week 4	20.3 (13.0)	22.4 (14.5)	0.72
Week 8	18.6 (8.6)	29.4 (10.6)	0.02
**Total cholesterol, mg/dL**			
Baseline	150 (34)	149 (27)	0.92
Week 4	145 (38)	152 (31)	0.67
Week 8	146 (26)	142 (34)	0.78
**HDL-C, mg/dL**			
Baseline	39 (11)	38 (11)	0.68
Week 4	38 (10)	39 (12)	0.85
Week 8	39 (11)	35 (10)	0.33
**LDL-C, mg/dL**			
Baseline	95 (29)	90 (28)	0.65
Week 4	92 (31)	95 (31)	0.83
Week 8	90 (21)	86 (28)	0.73
**Triglycerides, mg/dL**			
Baseline	81 (29)	108 (45)	0.08
Week 4	77 (30)	89 (41)	0.43
Week 8	84 (33)	112 (78)	0.31
**HOMA-IR**			
Baseline	2.04 (2.19)	2.96 (3.97)	0.48
Week 4	1.97 (1.82)	2.28 (2.99)	0.78
Week 8	2.19 (1.72)	2.85 (2.92)	0.54
**Pentoxifylline concentration, ng/mL**			
Week 4	0 (0)	89 (120)	
Week 8	0 (0)	61 (65)	

Notes: Data presented as means (standard deviations) or as No. (%). All 13 placebo subjects had samples available at all study visits. Of the 13 pentoxifylline subjects, 11 and 10 had samples available at weeks 4 and 8, respectively. hsCRP, high sensitivity C-reactive protein; IL-6, interleukin-6; HDL-C, high density lipoprotein-cholesterol; LDL-C, low density lipoprotein-cholesterol; TIMP-1, tissue inhibitor of metalloproteinase-1; sVCAM-1, soluble vascular cell adhesion molecule-1; sTNFRI and sTNFRII, soluble tumor necrosis factor-α receptors I and II; MCP-1, monocyte chemoattractant protein-1; IP-10, interferon-γ-induced protein-10; PAI-1 Ag, plasminogen activating inhibitor antigen-1; HOMA-IR, homeostatic model assessment-insulin resistance.

1 For comparisons between placebo and pentoxifylline groups.

### Safety

There were no serious adverse events during the trial. As shown in Table 5, most study participants reported at least one physical symptom during the trial, none of which were treatment-limiting. There were no significant differences between the number of total reported symptoms or laboratory abnormalities in the placebo group compared to the PTX group. All toxicities were considered grade 1 and not treatment-limiting except for one grade 2 neutropenia which prompted study discontinuation in 1 participant at week 4.

**Table 5:** Adverse Events during the Trial.

Characteristic	Placebo	Pentoxifylline	P-value^1^
**Symptoms**			
Total	21	14	0.73
Gastrointestinal	8	7	
Rash	2	1	
Flushing	2	0	
Headache	1	2	
Cough	2	0	
Other	6	4	
**Laboratory Abnormalities**			
Total	2	6	0.86
Neutropenia	0	2	
Hypokalemia	0	1	
Hyperkalemia	1	0	
Elevated liver function tests	1	1	
Hyperglycemia	0	1	
Dipstick proteinuria	0	1	

Notes: Data are cumulative number of adverse events.

1 For comparisons between placebo and pentoxifylline groups.

## Discussion

PTX did not reduce circulating markers of inflammation and did not improve arterial FMD, a measure of endothelial function, in this randomized, placebo-controlled trial of HIV-infected persons not requiring ART. PTX also did not reduce the levels of the leukocyte adhesion molecules sVCAM-1 or IP-10, although this effect was found in our smaller, open-label pilot study [14] and in our *in vitro* endothelial cell model combining TNF-α with HIV-secreted proteins [13]. The inflammatory marker sTNFRI surprisingly increased in the PTX arm compared to placebo.

We cannot discount the possibility that the increase in sTNFRI in the PTX group would have an adverse clinical impact if PTX were continued as a chronic intervention. However, we did not find a correlation between change in sTNFRI and FMD in this study, thus making it difficult to make any inference of the clinical relevance of the change in sTNFRI as it relates to endothelial function. In addition, no changes in other inflammatory markers were significantly different between groups, making the change in this one biomarker potentially a chance finding due to multiple testing. Previous studies have suggested that PTX reduces TNF-α expression *in vitro* and *in vivo* by inhibiting nuclear factor-kappa B and may thus even inhibit HIV replication [21], [22], [23], [24]. However, Clerici et al. found that TNF-α expression may actually increase during the first 12 weeks of use of PTX 400 mg thrice daily [25], a finding which is concordant our trial’s finding of significant increases in sTNFRI, a more stable circulating marker of TNF-α production than circulating TNF-α itself. These contrasting results may be explained by the fact that the study by Clerici et al. and the current trial included asymptomatic patients with relatively preserved CD4 cell counts who were not receiving ART as opposed to the previously mentioned studies that involved severely immunocompromised patients with higher levels of TNF-α. This suggests that PTX may be more beneficial in patients with reduced CD4 counts and who may have a greater inflammatory burden, a group we are currently studying in a separate PTX trial in patients initiating ART (NCT00864916).

Despite its purported beneficial effects on the endothelium, the influence of oral PTX on vascular function in humans has only previously been studied in HIV-negative, type 2 diabetics [26]. In that study, similar to the current report, there was no benefit of PTX on endothelial function. The characteristics of the participants in our smaller pilot study [14] were generally similar to those who enrolled in this larger and more definitive trial. The only appreciable differences were the greater number of black participants in the current trial compared to the pilot study (62% vs. 33%) and the greater body mass index in the current trial participants (26.9 vs. 20.6 kg/m^2^). However, neither black race nor body mass index were predictors of FMD change in this trial and so were unlikely to have led to these discrepant results. In addition, the PTX drug concentrations found at weeks 4 and 8 were comparable to those seen in our pilot trial. Of note, NTGMD did not change in either arm during this trial, suggesting that there were no changes in the inherent ability of the vascular endothelium to react to nitric oxide. Therefore, it is not clear why we observed the impressive improvements in FMD in our pilot trial and yet negative results in the current trial, although we cannot rule out the possibilities that the pilot trial’s results were due to chance or biased due to the open-label design.

FMD generally declined in this study cohort, with the largest reductions in FMD occurring in those with the highest FMD at baseline, thus, regression to the mean cannot be excluded. When examining baseline FMD and sex together, baseline FMD remained significantly associated with changes in FMD after 8 weeks with sex only marginally associated with this change. A larger sample size may have led to finding that sex remained independently associated with change in FMD. Of note, we cannot exclude the possibility that PTX may be beneficial in white HIV-infected women as none were enrolled in this trial.

Although this study was relatively small, the sample size was conservatively and justifiably based on our positive pilot results. Moreover, given the FMD outcomes observed, it would be unlikely to find differences between arms, let alone a positive benefit of PTX, in a larger trial. This sample had sufficient power to find differences in the inflammatory markers found to be significantly reduced in our pilot trial, namely sVCAM-1 and IP-10. However, we recognize that a larger sample potentially could have provided greater power to find differences in these secondary endpoint measures and allowed better assessments of the changes in FMD and biomarkers in specific subgroups.

Overall, PTX was well-tolerated. Compared to placebo, the PTX participants did not report a greater frequency of gastrointestinal adverse events which have been previously associated with the use of this drug [22]. Although there was not a statistically significant increase in laboratory toxicities with PTX, the two neutropenia events, one of which led to drug discontinuation, suggests that this particular adverse event should be monitored for closely in other trials of PTX. In addition, the two participants who were lost to follow-up were both randomized to the PTX arm, so we cannot exclude the possibility of PTX-related adverse events in these two participants.

Our trial is unique in that changes in FMD and the biomarkers purportedly associated with cardiovascular disease in the HIV-infected population were assessed over time without the confounding influence of ART. This was possible as the treatment guidelines at the time at which this study was performed recommended ART initiation only in those with CD4 cell counts <350/µL. With current recommendations to initiate ART immediately without regard to CD4 cell count, such studies will likely not be possible in the future. We also note that the negative results of this current trial do not necessarily extend to those receiving ART, although we will examine this possibility in the aforementioned second trial of PTX.

In conclusion, PTX did not reduce systemic inflammation or improve endothelial function in HIV-infected persons not requiring antiretroviral therapy. Additional research investigating the utility of other anti-inflammatory interventions is clearly needed.

## Acknowledgments

We thank Ms. Beth Zwickl, NP for study coordination, Mr. Jeffrey Waltz, RDCS for performing the vascular ultrasonography studies, and Mr. Jonathon Mathews, BS for data management. We also thank Dr. Homer Twigg and Ms. Patricia Smith for performing the flow cytometry studies, Dr. Russell Tracy and Ms. Elaine Cornell for performing the batched biomarker assays, and Ms. Bonnie Klank and Denise Cox for preparing the study drug and matching placebo. Most of all, we thank the study participants for their generous participation.

## Supporting Information

Checklist S1 **CONSORT checklist.**
(DOC)

Protocol and Consent S1 **The revised (February 5, 2011) trial protocol and informed consent form.**
(DOC)

## References

[1] Triant VA, Lee H, Hadigan C, Grinspoon SK (2007) Increased acute myocardial infarction rates and cardiovascular risk factors among patients with human immunodeficiency virus disease. J Clin Endocrinol Metab 92: 2506–2512. 17456578 10.1210/jc.2006-2190 PMC2763385

[2] Obel N, Thomsen HF, Kronborg G, Larsen CS, Hildebrandt PR, et al (2007) Ischemic heart disease in HIV-infected and HIV-uninfected individuals: a population-based cohort study. Clin Infect Dis 44: 1625–1631. 17516408 10.1086/518285

[3] Friis-Moller N, Reiss P, Sabin CA, Weber R, Monforte A, et al (2007) Class of antiretroviral drugs and the risk of myocardial infarction. New England Journal of Medicine 356: 1723–1735. 17460226 10.1056/NEJMoa062744

[4] Calmy A, Gayet-Ageron A, Montecucco F, Nguyen A, Mach F, et al (2009) HIV increases markers of cardiovascular risk: results from a randomized, treatment interruption trial. AIDS 23: 929–939. 19425222 10.1097/qad.0b013e32832995fa

[5] Kuller LH, Tracy R, Belloso W, De Wit S, Drummond F, et al (2008) Inflammatory and coagulation biomarkers and mortality in patients with HIV infection. PLoS Med 5: e203. 18942885 10.1371/journal.pmed.0050203 PMC2570418

[6] El-Sadr WM, Lundgren JD, Neaton JD, Gordin F, Abrams D, et al (2006) CD4+ count-guided interruption of antiretroviral treatment. N Engl J Med 355: 2283–2296. 17135583 10.1056/NEJMoa062360

[7] Grunfeld C, Delaney JAC, Wanke C, Currier JS, Scherzer R, et al (2009) Preclinical atherosclerosis due to HIV infection: carotid intima-medial thickness measurements from the FRAM study. AIDS 23: 1841–1849. 19455012 10.1097/QAD.0b013e32832d3b85 PMC3156613

[8] Gokce N, Keaney JF Jr, Hunter LM, Watkins MT, Nedeljkovic ZS, et al (2003) Predictive value of noninvasively determined endothelial dysfunction for long-term cardiovascular events in patients with peripheral vascular disease. Journal of the American College of Cardiology 41: 1769–1775. 12767663 10.1016/s0735-1097(03)00333-4

[9] Suwaidi JA, Hamasaki S, Higano ST, Nishimura RA, Holmes DR Jr, et al (2000) Long-term follow-up of patients with mild coronary artery disease and endothelial dysfunction. Circulation 101: 948–954. 10704159 10.1161/01.cir.101.9.948

[10] Kang SM, Chung N, Kim JY, Koo BK, Choi D, et al (2002) Relation of vasodilator response of the brachial artery to inflammatory markers in patients with coronary artery disease. Echocardiography 19: 661–667. 12487635 10.1046/j.1540-8175.2002.00661.x

[11] Suessenbacher A, Frick M, Alber HF, Barbieri V, Pachinger O, et al (2006) Association of improvement of brachial artery flow-mediated vasodilation with cardiovascular events. Vasc Med 11: 239–244. 17390547 10.1177/1358863x06075006

[12] Modena MG, Bonetti L, Coppi F, Bursi F, Rossi R (2002) Prognostic role of reversible endothelial dysfunction in hypertensive postmenopausal women. Journal of the American College of Cardiology 40: 505–510. 12142118 10.1016/s0735-1097(02)01976-9

[13] Green LA, Kim C, Gupta SK, Rajashekhar G, Rehman J, et al. (2012, in press) Pentoxifylline Reduces Tumor Necrosis Factor-alpha and HIV-Induced Vascular Endothelial Activation. AIDS Research and Human Retroviruses. 10.1089/aid.2011.0385 PMC3448099 22463742

[14] Gupta SK, Johnson RM, Mather KJ, Clauss M, Rehman J, et al (2010) Anti-inflammatory treatment with pentoxifylline improves HIV-related endothelial dysfunction: a pilot study. AIDS 24: 1377–1380. 20559042 10.1097/QAD.0b013e3283396024 PMC2891092

[15] Hsue PY, Hunt PW, Wu Y, Schnell A, Ho JE, et al (2009) Association of abacavir and impaired endothelial function in treated and suppressed HIV-infected patients. AIDS 23: 2021–2027. 19542863 10.1097/QAD.0b013e32832e7140 PMC2785446

[16] Gupta SK, Shen C, Moe SM, Kamendulis LM, Goldman M, et al. (2012, in press.) Worsening endothelial function with efavirenz compared to protease inhibitors: A 12-month prospective study. PLOS ONE. 10.1371/journal.pone.0045716 PMC3447812 23029197

[17] Corretti MC, Anderson TJ, Benjamin EJ, Celermajer D, Charbonneau F, et al (2002) Guidelines for the ultrasound assessment of endothelial-dependent flow-mediated vasodilation of the brachial artery: a report of the International Brachial Artery Reactivity Task Force. Journal of the American College of Cardiology 39: 257–265. 11788217 10.1016/s0735-1097(01)01746-6

[18] Matthews DR, Hosker JP, Rudenski AS, Naylor BA, Treacher DF, et al (1985) Homeostasis model assessment: insulin resistance and beta-cell function from fasting plasma glucose and insulin concentrations in man. Diabetologia 28: 412–419. 3899825 10.1007/BF00280883

[19] Lachin JM (1999) Worst-rank score analysis with informatively missing observations in clinical trials. Controlled Clinical Trials 20: 408–422. 10503801 10.1016/s0197-2456(99)00022-7

[20] Berger VW, Exner DV (1999) Detecting selection bias in randomized clinical trials. Controlled Clinical Trials 20: 319–327. 10440559 10.1016/s0197-2456(99)00014-8

[21] Dezube BJ, Lederman MM (1995) Pentoxifylline for the treatment of HIV infection and its complications. Journal of Cardiovascular Pharmacology 25 Suppl 2S139–142. 8699854 10.1097/00005344-199500252-00029

[22] Dezube BJ, Lederman MM, Spritzler JG, Chapman B, Korvick JA, et al (1995) High-dose pentoxifylline in patients with AIDS: inhibition of tumor necrosis factor production. National Institute of Allergy and Infectious Diseases AIDS Clinical Trials Group. Journal of Infectious Diseases 171: 1628–1632. 7769305 10.1093/infdis/171.6.1628

[23] Dezube BJ, Pardee AB, Chapman B, Beckett LA, Korvick JA, et al (1993) Pentoxifylline decreases tumor necrosis factor expression and serum triglycerides in people with AIDS. NIAID AIDS Clinical Trials Group. Journal of Acquired Immune Deficiency Syndromes 6: 787–794. 8099612

[24] Fazely F, Dezube BJ, Allen-Ryan J, Pardee AB, Ruprecht RM (1991) Pentoxifylline (Trental) decreases the replication of the human immunodeficiency virus type 1 in human peripheral blood mononuclear cells and in cultured T cells. Blood 77: 1653–1656. 1707692

[25] Clerici M, Piconi S, Balotta C, Trabattoni D, Capetti A, et al (1997) Pentoxifylline improves cell-mediated immunity and reduces human immunodeficiency virus (HIV) plasma viremia in asymptomatic HIV-seropositive persons. Journal of Infectious Diseases 175: 1210–1215. 9129088 10.1086/593570

[26] Bilsborough W, O’Driscoll G, Stanton K, Weerasooriya R, Dembo L, et al (2002) Effect of lowering tumour necrosis factor-alpha on vascular endothelial function in Type II diabetes. Clin Sci 103: 163–169. 12149108 10.1042/cs1030163

