# Supplementary material for: Pentoxifylline, Inflammation, and Endothelial Function in HIV-Infected Persons: A Randomized, Placebo-Controlled Trial
Source: PLoS One. 2013 Apr 9;8(4):e60852. doi: 10.1371/journal.pone.0060852 (PMC3621886; doi:10.1371/journal.pone.0060852)
Supplement: Protocol and Consent S1 — The revised (February 5, 2011) trial protocol and informed consent form. (DOC) [file pone.0060852.s002.doc]

**A Randomized, Placebo-Controlled Trial of Pentoxifylline to Improve Endothelial Function in HIV-Infected Patients Not Requiring Antiretroviral Therapy**

Version 2.0

February 5, 2011

Funded by: National Institutes of Health

National Heart, Lung, and Blood Institute

R01 HL095149-01

Principal Investigator: ________________________________ _______________

Samir K. Gupta, M.D., M.S. Date

Division of Infectious Diseases

Indiana University School of Medicine

TABLE OF CONTENTS

PROTOCOL TEAM ROSTER……………………………………………………………...6

ABBREVIATIONS………………………………………………………………………....7

SCHEMA……………………………………………………………………………………8

1. STUDY OBJECTIVES……………………………………………………………...9
   1. PRIMARY OBJECTIVE…..………………………………………………..9
   2. SECONDARY OBJECTIVES………………………………………………9
2. INTRODUCTION…………………………………………………………………...9
   1. HYPOTHESIS……………………………………………………………….9
   2. BACKGROUND…………………………………………………………….9
      1. ENDOTHELIAL DYSFUNCTION AND

CARDIOVASCULAR DISEASE…………………………………..9

- - 1. HIV AND CARDIOVASCULAR EVENTS……………………….10
    2. HIV AND ENDOTHELIAL DYSFUNCTION…………………….10
    3. THE ROLE OF HIV-INDUCED INFLAMMATION ON

ATHEROSCLEROSIS AND ENDOTHELIAL DYSFUNCTION..11

- 1. PRELIMINARY STUDIES………………………………………………...11
     1. MEASUREMENTOF IN VIVO ENDOTHELIAL FUNCTION

USING THE FLOW-MEDIATED DILATION TECHNIQUE……11

- - 1. COMPARISON OF FLOW-MEDIATED DILATION IN HIV-

INFECTED AND HIV-UNINFECTED SUBJECTS………………12

- - 1. EFFECTS OF ANTI-INFLAMMATORY MEDICATION ON

ENDOTHELIAL FUNCTION IN HIV-INFECTED SUBJECTS…13

- 1. PENTOXIFYLLINE (TRENTAL®)……………………………………….16
     1. DESCRIPTION…………………………………………………….16
     2. CLINICAL PHARMACOLOGY…………………………………..16
     3. INDICATIONS AND USAGE……………………………………..17
     4. CONTRAINDICATIONS…………………………………………..17
     5. PRECAUTIONS……………………………………………………17
        1. GENERAL PRECAUTIONS………………………………17
        2. DRUG INTERACTIONS…………………………………..18
        3. CARCINOGENESIS………………………………………18
        4. USE IN PREGNANCY…………………………………….18
        5. NURSING MOTHERS……………………………………..18
     6. ADVERSE REACTIONS……………………………..…………..18
     7. OVERDOSAGE……………………………….…………………..19
     8. DOSAGE AND ANDMINISTRATION……….………………….19

2.5 STUDY RATIONALE…………………………………………………….19

1. STUDY DESIGN………………………………………………………………….. 20
   1. OVERVIEW……………………………………………………………….. 20
   2. SCREENING VISITS……………………………………………………… 20
   3. MAIN STUDY VISITS…………………………………………………… 21
   4. BRACHIAL ARTERY REACTIVITY INDICES………………………… 22
   5. STUDY DURATION AND SUBJECT RETENTION……………………. 22
2. SELECTION AND ENROLLMENT CRITERIA………………………………….23
   1. INCLUSION CRITERIA………………………………………………….. 23
   2. EXCLUSION CRITERIA…………………………………………………. 23
   3. COENROLLMENT GUIDELINES……………………………………….. 25
3. STUDY TREATMENT……………………….…………………………………. 25
   1. REGIMEN, ADMINISTRATION AND DURATION…………………… 25
   2. PRODUCT FORMULATION AND PREPARATION………………….. 25
   3. STUDY DRUG ACCOUNTABILITY AND DISPENSING ……………. 25
   4. CONCOMITANT MEDICATIONS……………………………………… 25

5.4.1 PROHIBITED MEDICATIONS…………………………………. 25

5.4.2 PRECAUTIONARY MEDICATIONS…………………………… 26

1. CLINICAL AND LABORATORY EVALUATIONS…………………………… 27
   1. SCHEDULE OF EVENTS………………………………………………. 27
   2. DEFINITIONS FOR SCHEDULE OF EVENTS……………………….. 28
      1. SCREENING EVALUATIONS………………………………….. 28
      2. MAIN STUDY EVALUATIONS………………………………… 28
      3. STUDY DISCONTINUATION EVALUATIONS………………. 28
   3. DEFINITIONS FOR SCHEDULE OF EVENTS-SPECIAL

INSTRUCTIONS AND DEFINITIONS OF EVALUATIONS………….. 28

6.3.1 DOCUMENTATION OF HIV-1 INFECTION…………………… 28

6.3.2 MEDICAL HISTORY…………………………………………….. 28

6.3.3 MEDICATION HISTORY………………………………………… 29

6.3.4 CONCOMITANT MEDICATIONS………………………………. 29

6.3.5 PENTOXIFYLLINE DOSING MODIFICATIONS…………….. 29

6.3.6 FAMILY HISTORY………………………………………………. 29

6.3.7 SMOKING HISTORY……………………………………………. 29

6.3.8 CLINICAL ASSESSMENTS …………………………………….. 29

6.3.8.1 HEIGHT…………………………………………………… 29

6.3.8.2 WEIGHT……………………………………………………30

6.3.8.3 RESTING BLOOD PRESSURE…………………………... 30

6.3.8.4 RESTING HEARTRATE…………………………………. 30

6.3.8.5 WAIST AND HIP MEASUREMENTS…………………… 30

6.3.8.6 BRIEF PHYSICAL EXAMINATION……………………. 30

6.3.8.7 SIGNS AND SYMPTONS………………………………… 31

6.3.8.8 DIAGNOSES……………………………………………….31

6.3.8.9 ADHERENCE ASSESSMENTS………………………….31

6.3.8.10ADHERENCE OF SUCCESSFUL BLINDING…………31

6.3.8.11LABORATORY EVALUATIONS………………………31

6.3.8.12SPECIMEN COLLECTION, PROCESSING, LABELING

AND STORAGE…………………………………………..33

6.3.8.13BRACHIAL ARTERY ULTRASOUND

MEASUREMENTS………………………………………..34

1. ADVERSE EVENT MANAGEMENT…………………………………………… 34

7.1 GRADE 1 OR 2 TOXICITY/ADVERSE EVENT………………………… 34

7.2 GRADE 3 OR 4 TOXICITY/ADVERSE EVENT………………………… 34

7.3 PENTOXIFYLLINE………………………………………………………. 35

7.3.1 DROP IN HEMOGLOBIN/BLEEDING EPISODE………………. 35

7.3.2 RISE IN LIVER FUNCTION TESTS…………………………… 35

7.3.3 ELEVATION OF SERUM CREATININE/DECREASE IN CALCULATED CREATININE CLEARANCE…………………. 35

7.3.4 RASH……………………………………………………………… 35

7.3.5 GASTROINTESTINAL…………………………………………… 36 7.3.6 NEUROLOGIC…………………………………………………… 36

7.4 NITROGLYCERIN……………………………………………………….. 36

7.4.1 HYPOTENSION………………………………………………….. 36

7.4.2 HEADACHE …………………………………………………….. 37

1. CRITERIA FOR STUDY DISCONTINUATION………………………………. 37
2. STATISTICAL CONSIDERATION AND DATA MANAGEMENT…………… 37

9.1 GENERAL CONDSIDERATIONS……………………………………….37

9.2 SAMPLE SIZE JUSTIFICATION…………………………………………37

9.3 DATA ANALYSIS…………………………………………………………38

9.3.1 GENERAL DATA ANALYSIS……………………………………38

9.3.2 PRIMARY ENDPOINT ANALYSIS………………………………38

9.3.3 SECONDARY ENDPOINTS ANALYSES………………………..39

9.3.4 RANDOMIZATION………………………………………………..39

9.4 STUDY POPULATION…………………………………………………….39

9.5 STUDY DURATION……………………………………………………….39

9.6 DATA MANAGEMENT……………………………………………………40

10.0 HUMAN SUBJECTS RESEARCH AND PROTECTION……………………….. 40

10.1 INVESTIGATOR TRAINING……………………………………………. 40

10.2 RISKS TO HUMAN SUBJECTS…………………………………………. 40

10.2.1 HUMAN SUBJECTS INVOLVEMENT AND

CHARACTERISTICS…………………………………………….. 40 10.2.2 SOURCES OF MATERIALS……………………………………. 41

10.2.3 POTENTIAL RISKS………………………………………………. 42

10.3 ADEQUACY OF PROTECTION AGAINST RISKS……………………. 42

10.3.1 RECRUITMENT AND INFORMED CONSENT………………… 42

10.3.2 PROTECTION AGAINST RISK…………………………………. 42

10.3.2.1 CONFIDENTIALITY…………………………………… 43

10.3.2.2 BLOOD DRAWS…………………………………………43

10.3.2.3 PENTOXIFYLLINE………………………………………43

10.3.2.4 NITROGLYCERIN……………………………………….43

10.3.2.5 WITHHOLDING ANTIRETROVIRAL THERAPIES…..43

10.3.2.6ADVERSE EVENT MANAGEMENT

AND REPORTING………………………………………44

10.3.2.7DATA AND SAFETY MONITORING COMMITTEE …44

10.4 POTENTIAL BENEFITS OF THE PROPOSED RESEARCH TO THE SUBJECTS AND OTHERS………………………………………………. 44

10.5 IMPORTANCE OF THE KNOWLEDGE TO BE GAINED…………….. 44

10.6 DATA AND SAFETY MONITORING PLAN……………………………44

10.7 INCLUSION OF WOMEN AND MINORITIES………………………….45

10.8 INCLUSION OF CHILDREN……………………………………………. 45

11.0 REFERENCES…………………………………………………………………….46

APPENDIX I: FORMULAS AND DEFINITIONS………………………………………. 55

INFORMED CONSENTS………………………………………………………………… 56

PROTOCOL TEAM ROSTER

# Principal Investigator

Samir K. Gupta, M.D., M.S.

Division of Infectious Diseases

Indiana University School of Medicine

Wishard Hospital, OPW-430

1001 W. 10^th^ Street

Indianapolis, IN 46202

Phone: 317-630-6602

Fax: 317-630-7522

Email: sgupta1@iupui.edu

# Co-Investigators

Raymond M. Johnson, M.D., Ph.D.

Division of Infectious Diseases

Indiana University School of Medicine

Kieren J. Mather, M.D., F.R.C.P.C.

Division of Endocrinology and Metabolism

Indiana University School of Medicine

Johnny He, Ph.D.

Division of Infectious Diseases

Indiana University School of Medicine

Matthias Clauss, Ph.D.

Department of Physiology

Indiana University School of Medicine

Michael P. Dubé, M.D.

Division of Infectious Diseases

# University of Southern California

Homer L. Twigg, M.D., Ph.D.

Division of Pulmonary Medicine

Indiana University School of Medicine

Desta Zeruesenay, Ph.D.

Division of Clinical Pharmacology

Indiana University School of Medicine

In-Woo Park, Ph.D.

Division of Infectious Diseases

Indiana University School of Medicine

Biostatisticians

Ziyue Liu, Ph.D.

Division of Biostatistics

Indiana University School of Medicine

Deming Mi, M.S.

Division of Biostatistics

Indiana University School of Medicine

# Data Managers

Jonathon Mathews, B.S.

Division of Biostatistics

Indiana University School of Medicine

# Research Assistants

Beth Zwickl, NP, APN-BC, ACRP

Infectious Diseases Research Clinic

Indiana University School of Medicine

Jeffrey Waltz, RDCS

Infectious Diseases Research Clinic

Indiana University School of Medicine

Sheila Ellinger, RN, MSN, CCRP

Infectious Diseases Research Clinic

Indiana University School of Medicine

ABBREVIATIONS

FMD Flow-mediated dilation BSA Body Surface Area

NTGMD Nitroglycerin-mediated dilation BMI Body Mass Index

HIV Human immunodeficiency virus PTX Pentoxifylline

AIDS Acquired immunodeficiency syndrome hsCRP High sensitivity C-reactive protein

cART combination antiretroviral therapy AST Aspartate aminotransferase

NF-κB Nuclear Factor-kappa Beta ALT Alanine aminotransferase

QUICKI Quantitative Insulin Check Index LFT Liver function test

HDL High density lipoprotein VCAM-1 Vascular cell adhesion molecule-1

LDL Low density lipoprotein MCP-1 Monocyte chemoattractant protein-1

CRF Case Report Form

IDRC Infectious Diseases Research Clinic

ICRC Indiana Clinical Research Center

SAE Serious Adverse Experience

GFR Glomerular Filtration Rate

SCr Serum Creatinine

BUN Blood Urea Nitrogen

TNF-α Tumor Necrosis Factor-alpha

SCHEMA

DESIGN

This is a randomized, double-blind, placebo-controlled trial comparing the efficacy and safety of pentoxifylline (PTX) vs. placebo in the treatment of endothelial dysfunction in a total of 26 subjects (13 per arm) HIV-infected patients seen at the Indiana University Medical Center with CD4 cell counts greater than 350/µL and not receiving antiretroviral therapy. Enrolled subjects will have their brachial artery flow-mediated dilation (FMD), a measure of endothelial function, and other cardiovascular and immunologic parameters measured at baseline and again after 4 and 8 weeks of treatment with PTX.

## OBJECTIVES

The primary objective of this study is to evaluate the effect of 400mg of PTX given orally thrice daily for 8 weeks on brachial artery FMD in HIV-1-infected patients not currently receiving antiretroviral therapy and with CD4 cell counts > 350/µL. Secondary objectives are to evaluate the effect of 4 weeks of PTX on FMD and also to determine the effects of PTX on various immunologic, metabolic, cardiovascular, and safety parameters at weeks 4 and 8.

## DURATION

Each individual subject will be followed for approximately 11 weeks. The total duration of this study will be approximately 22 months.

## POPULATION

## All subjects will be HIV-infected, 18 years of age or older, free of antiretroviral therapies for six months prior to enrollment, and not be expected to need antiretroviral therapies during the course of this study. Subjects will be recruited from the infectious diseases outpatient clinics of Wishard Hospital and the Indiana University Hospital.

# SAMPLE SIZE

Up to 50 subjects will be screened to achieve the goal of 26 randomized subjects.

1.0 STUDY OBJECTIVES

- 1. Primary Objective
     1. Determine the effect of pentoxifylline 400mg po tid given for 8 weeks on brachial artery FMD in HIV-infected subjects not currently requiring antiretroviral therapy and with CD4 cell counts ≥ 350/µL.
  2. Secondary Objectives
     1. Evaluate the safety and tolerability of pentoxifylline 400mg po tid given over 8 weeks in HIV-infected subjects not currently requiring antiretroviral therapy and with CD4 cell counts ≥ 350/µL.
     2. Determine the effect of pentoxifylline 400mg po tid given for 4 weeks on brachial artery FMD in HIV-infected subjects not currently requiring antiretroviral therapy and with CD4 cell counts ≥ 350/µL.

- - 1. Evaluate the effects of pentoxifylline 400mg po tid on serum MCP-1, sVCAM-1, IP-10, MMP-9, TIMP-1, PAI-1 active, hsCRP, fasting lipoprotein fractions/triglycerides, and peripheral blood immune activation (percentage of CD8+/CD38+/HLA-DR+ T cells) and correlate these changes with changes in FMD.

1. INTRODUCTION

2.1 Hypothesis

In HIV-infected subjects not requiring cART, pentoxifylline for 8 weeks will improve flow-mediated dilation of the brachial artery more than those receiving placebo.

2.2 Background

2.2.1 Endothelial dysfunction and cardiovascular disease

Endothelial dysfunction is a key, initial step in the development of atherosclerosis. The vascular endothelium is an autocrine, paracrine, and endocrine organ that regulates vascular tone, thrombosis, and inflammation [1, 2]. Endothelial dysfunction results in an impaired availability of endothelium-derived nitric oxide (NO), which is responsible for vascular dilation and the prevention of proatherogenic processes, such as platelet aggregation, vascular smooth muscle proliferation, and increased adhesion of leukocytes to the endothelium [3].

Coronary artery endothelial dysfunction was initially measured using invasive cardiac catheterization. To minimize the risks involved with catheterization, a non-invasive technique using high-resolution ultrasound has been developed to measure changes in brachial artery diameter due to a stimulus [4]. Measuring brachial artery reactivity in this manner correlates closely to coronary endothelial function [5, 6]. Brachial ultrasound measures the brachial artery diameter at baseline and then after endothelium-dependent and endothelium-independent stimuli are administered [7]. The endothelium-dependent stimulus is hyperemic blood flow triggered after the release of an inflated blood pressure cuff around the forearm. The shear stress from the increased blood flow subsequently causes endogenous vasodilators, predominantly NO, to be released from the endothelium which then relaxes the vascular smooth muscle. This flow-mediated dilation (FMD) of the brachial artery is thus a measure of the endothelium’s ability to respond normally to hyperemic shear stress. Because FMD is impaired when the vascular smooth muscle itself does not properly react to normal NO release from the endothelium, a second stimulus that directly relaxes smooth muscle is applied to determine the smooth muscle’s functional capacity. This endothelium-independent stimulus is exogenous nitroglycerin. Thus, nitroglycerin-mediated dilation (NTGMD) provides an internal control to which FMD can be compared. If FMD is low, but NTGMD is normal, the endothelium, and not the smooth muscle, can be safely assumed to be dysfunctional.

The major predictors of FMD in cross-sectional studies include traditional cardiovascular disease risk factors, such as diabetes, insulin resistance, hypertension, and smoking [4, 8-11]. Baseline brachial artery diameter, which is a key determinant to the degree of reactive flow, is strongly and inversely correlated with FMD in both HIV-negative [4] and HIV-infected subjects [12]. Lower FMD appears to predict future cardiovascular events in most [13-17], but not all [18, 19], prognostic studies.

FMD and NTGMD are calculated as the percent increases, respectively, in diameter over baseline after hyperemic flow and nitroglycerin stimuli are implemented. Levels of FMD and NTGMD in normal, healthy populations are in the ranges of 7-12% and 16-20%, respectively [9, 20]. An absolute increase of at least 3% in FMD after a therapeutic intervention is employed has been associated with reduced risk of future cardiovascular events in general population studies [21, 22].

2.2.2 HIV and cardiovascular events

It is increasingly apparent that HIV-infected individuals are at higher risk for cardiovascular events compared to the general population [23-26]. Much of this increased risk for both myocardial infarction and stroke has previously been attributed to cART [27-31] and the adverse metabolic profile associated with its use [31]. The Strategies for Management of Antiretroviral Therapy (SMART) Study Group recently highlighted the contribution of untreated HIV on cardiovascular disease. In this study, CD4 count-guided interruption of cART unexpectedly increased the rate of cardiovascular events compared to those who continued therapy [32]. This relationship persisted even when adjusting for most recent CD4 count and viral load [33]. Notably, the increased risk was not attenuated when subjects restarted cART. These results suggest that even short periods of untreated HIV may promote the development of vascular disease.

2.2.3 HIV and endothelial dysfunction

It has been shown that individual HIV proteins, and in particular soluble Tat and gp120, may directly cause endothelial activation [34] and permeability [35, 36] through inflammatory [37-39], oxidative stress [40, 41], and apoptotic pathways [42, 43]. In vivo physiologic endothelial dysfunction also appears to be more frequent in HIV-infected patients compared to those without HIV, even after adjusting for traditional cardiovascular risk factors [44-49]. Much of the focus on the causes of HIV-related endothelial dysfunction has centered on antiretroviral toxicity. Older generation protease inhibitors (indinavir) and thymidine analog nucleoside inhibitors (stavudine, zidovudine) have been associated with impaired endothelial function due to direct effects of the drug on the endothelium or due to drug-related metabolic dysregulation [47-54]. However, more recent data suggest that newer protease inhibitors (lopinavir/ritonavir, atazanavir) are not associated with endothelial dysfunction [12, 48, 55, 56].

The effects of newer cART regimens on FMD have recently been studied. In this trial, cART regimens including the non-nucleoside analog efavirenz, the protease inhibitor lopinavir/ritonavir, or a combination of the two were similarly effective in improving significantly impaired pre-cART flow-mediated dilation in 75 previously antiretroviral-naïve subjects [57]. After 24 weeks of therapy, FMD significantly improved with no differences between treatment regimens. Of all variables measured, only the magnitude of reduction in viral load significantly correlated with improvement in endothelial dysfunction, albeit weakly (r=0.30, p=0.01). Of note, FMD did not improve to normal levels in this study, suggesting virologic suppression with cART does not fully ameliorate HIV-induced endothelial dysfunction due to HIV. Further evidence suggesting that the cART may improve endothelial activation during the initial phase of therapy was demonstrated in a prospective, longitudinal study of HAART-naïve HIV-infected subjects [58]. In this study, the endothelial activation markers soluble vascular cell adhesion marker-1 (sVCAM-1), soluble intracellular cell adhesion marker-1 (sICAM-1), and von Willebrand Factor (vWF) all improved after a mean duration of cART use of 9 months. Similar to the Torriani study, the degree of improvement in these markers was similar between those using NNRTI or PI-based cART. Of note, the levels of these markers after cART remained significantly abnormal compared to a group of HIV-uninfected controls, suggesting that endothelial activation is still being triggered to a higher level despite controlling viremia with cART. Other mechanisms, such as increased inflammation, must play a role in this continued endothelial dysfunction.

2.2.4 The role of HIV-induced inflammation on atherosclerosis and endothelial dysfunction

It has been firmly established in the general population that immune cells play a critical role in the formation and progression of atherosclerotic lesions [59, 60]. Inflammatory mediators, either derived from existing immune cells from within vascular lesions or from extrinsic sources, can weaken and disrupt the atheromatous plaque [61], thereby resulting in an acute vascular event [62]. Increased levels of inflammatory markers, such as sVCAM-1, have been associated with endothelial dysfunction measured using brachial artery flow-mediated dilation [11, 63, 64]. Therapeutic reductions in such inflammatory markers have also been associated with improvement in FMD in HIV-uninfected subjects [65]. Extrinsic sources of inflammation also increase the risk for cardiovascular events [66-69].

The histology of coronary and aortic tissue samples from cART-naïve HIV-infected patients shows significantly increased leukocyte adhesion to the endothelium compared to HIV-uninfected controls [70-72]. These endothelial lesions are juxtaposed on smooth muscle hyperproliferation and luminal narrowing reminiscent of chronic allograft vasculopathy in organ transplant recipients [73].. These vascular abnormalities are also associated with upregulation of tumor necrosis factor-alpha (TNF-α), monocyte chemoattractant protein-1 (MCP-1), and vascular cell adhesion molecule-1 (VCAM-1). These cytokines are involved in attracting monocytes into these lesions which may foster HIV replication within the endothelium and smooth muscle and subsequently accelerate development of atherosclerosis [73-75]. Polymorphisms of MCP-1 that lead to increased levels of MCP-1 have been shown to be associated with worse atherosclerosis in HIV-infected subjects [76, 77].

HIV induces NF-κB activity in monocytes [78-80] and in endothelial cells [81] with subsequent production of pro-inflammatory cytokines [82], such as IL-6 and TNF-α. Multiple studies have shown that HIV induces the upregulation of leukocyte adhesion molecules on the endothelium [83]. Elevated baseline levels of the inflammatory markers IL-6 and amyloid-P in the SMART trial were significantly associated with ensuing cardiovascular events [84], thereby substantiating the role of inflammation in HIV-related cardiovascular disease. These results strongly suggest that inflammation, especially pathways involving monocyte attraction and attachment to the endothelium, is an important determinant of HIV-related vasculopathy.

Even when HIV is controlled with antiretroviral therapy, heightened systemic inflammation [85, 86] and immune activation [87, 88] may still persist. One source of this continued inflammation may be the gut. Recent data strongly suggest that intestinal mucosal effector T cells are quickly depleted in acute HIV infection [89] and during all stages of disease [90]. Moreover, cART in chronically infected patients does not completely restore gut CD4 stores [91] with only partial mucosal repair and ongoing inflammatory gene expression [92]. In fact, it has been demonstrated that persistent bacterial translocation across the disrupted gut lining, as measured by increased levels of lipopolysaccharide, may be a source for continued immune activation in HIV-infected patients [93]. In this latter study, increased immune activation was defined as the proportion of activated CD8+ cells (CD8+CD38+/HLA-DR+) measured by flow cytometry. This is an accepted and standard definition of immune activation which has correlated with AIDS progression [94-96]. This indirect source of continuing systemic inflammation may then contribute to the impaired endothelial function seen in HIV-infected patients receiving cART with undetectable viral loads. Therefore, controlling inflammation, regardless of the source, may significantly improve vascular function in HIV-infected subjects.

2.3 Preliminary Studies

2.3.1 Measurement of in vivo endothelial function using the flow-mediated dilation technique

Our laboratory can reliably measure in vivo endothelial function using the brachial artery reactivity technique to measure flow-mediated dilation (FMD) [12, 97, 98] as outlined in consensus guideline recommendations [20].

In brief, a 10 mHZ linear array vascular probe for B-mode imaging is used to visualize the brachial artery. ECG leads will be placed on the subject’s chest in order to track the imaging with the cardiac cycle. A small forearm cuff is placed on the widest part of the subject’s proximal forearm (approximately 1-2 cm distal to the antecubital fossa). The brachial artery will then be located with the image optimized for best resolution of all three layers of the anterior and posterior walls (with special emphasis on identifying the media-adventitia interface). Three consecutive cardiac cycles of B-mode images of the brachial artery to the disks will be acquired and stored. After this baseline evaluation, the measurement of endothelium-dependent vasodilation, or FMD, due to increased blood flow will begin. The forearm pressure cuff will be inflated to 250 mmHg for 5 minutes. At that time, imaging will be switched to B-mode. At 60 and 90 seconds after cuff deflation, three consecutive cardiac cycles of B-mode images of the brachial artery to the disc will be acquired and stored. The greater of the two diameters measured at these time points will be used for FMD analysis. After 15 minutes from time of cuff deflation, the measurement of endothelium-independent vasodilation, or NTGMD, will begin. Nitroglycerin (400 mcg SL) will then be administered. Three minutes later, three consecutive cardiac cycles of B-mode of the brachial artery will be recorded. All diameter measurements will be measured when the cardiac cycle is at the R-wave. All diameter measurements will be made in triplicate and averaged to improve precision.

Dr. Gupta and Mr. Waltz (our group’s registered vascular technician) have undergone certified training in these procedures through the University of Wisconsin Brachial Artery Reactivity Testing Symposium. We have now performed over 300 brachial reactivity testing procedures in our vascular laboratory over the past two years. As an example of the reproducibility of results in our laboratory, we performed paired FMD studies in 12 healthy volunteers. The intraclass correlations for baseline brachial artery diameter measurements and for FMD were 0.97 and 0.73, respectively, which compare favorably with the results from other laboratories [50, 99].

2.3.2 Comparison of flow-mediated dilation in HIV-infected and HIV-uninfected subjects

The published studies to date comparing FMD in those with and without HIV infection have all included subjects receiving cART. Because certain antiretrovirals, either directly or indirectly through their metabolic effects, may confound FMD results, it has been difficult to assess the role of HIV itself on endothelial function in these studies. The AIDS Clinical Trials Group [57] study of FMD in antiretroviral-naïve subjects suggested that endothelial function was impaired prior to initiation of cART, but no direct comparison was made with an HIV-uninfected control group. In addition, these subjects all were initiating cART, presumably for low CD4 count or symptomatic HIV infection, thereby precluding assessment of those with better immune function.

We compared a group of HIV-infected subjects with a wide spectrum of CD4 cell counts [median 362 (range, 25-814) cells/µL] and not receiving cART (N=41) with a group of HIV-uninfected subjects (N=14). None of these subjects had known vascular disease, diabetes, or hypertension. The median FMD in the HIV-infected group of 4.3% is similar to that reported by Torriani et al. As shown in Figure 1, the FMD in the uninfected group was significantly higher (p=0.044) than in the HIV-infected group. There were no significant correlations between FMD and either CD4 count or viral load in the HIV-infected group.

Although this analysis suggests that HIV is associated with impaired endothelial function, we did not assess for variables that might have confounded these results, such as body size, metabolic parameters, or smoking status. We also did not assess inflammatory or endothelial activation biomarkers in the HIV-negative group. A larger comparative study that tightly matches and adjusts these two groups for these variables would allow a definitive investigation into the relationship between untreated HIV and endothelial dysfunction. Such a study would also provide a novel opportunity to measure biomarkers that might suggest mechanisms underlying the relationship between HIV and endothelial dysfunction. Ex vivo samples from these subjects could also be used to investigate the mechanistic roles of HIV and inflammatory mediators on endothelial cell biology.

Figure 1. Comparison of FMD between HIV-infected and uninfected subjects.


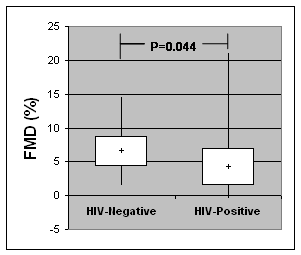


2.3.3 Effects of anti-inflammatory medications on endothelial function in HIV-infected subjects

Because cART alone may not fully reverse endothelial dysfunction, we postulated that agents that act to reduce inflammation through pathways not dependent on virologic replication may improve FMD. We performed pilot trials of two such medications. Because our specific goal was to evaluate anti-inflammatory drugs that were inexpensive, already widely available, relatively non-toxic, and without known drug interactions with HIV antiretrovirals, we chose to study salsalate and pentoxifylline.

Salsalate (SSA) is an FDA-approved drug, now available in generic preparations, with proven anti-inflammatory effects in patients with rheumatoid arthritis [100-103]. Salsalate is rapidly converted to salicylate after absorption. Salicylates may reduce systemic inflammation, as well HIV replication, via inhibition of NF-κB [80, 104]. Equivalent levels of plasma salicylate are achieved with 3 gm/day of SSA and 3.9 gm/day of aspirin [105, 106]. Unlike aspirin, salsalate is non-acetylated, thereby resulting in little antiplatelet action [106, 107] and reduced cyclo-oxygenase inhibition [108, 109]. This consequently reduces the risk of gastrointestinal bleeding [110, 111] compared to aspirin. SSA had not previously been studied for its impact on endothelial function. However, this drug would possibly provide a ‘cleaner’ and likely safer alternative to aspirin in testing the hypothesis that specifically inhibiting NF-κB using salicylates would improve FMD.

Pentoxifylline (PTX) is a generic drug initially approved for treatment of claudication, presumably because of its ability to improve red cell morphology. However, PTX, a phosphodiesterase inhibitor, also has been shown to block TNF-α production by monocytes and, like salsalate, inhibit NF-κB [112, 113]. PTX has been shown to reduce HIV-1 replication in mononuclear cells [114] by blocking HIV long terminal repeat (LTR) gene expression [115, 116]. At doses of 400mg thrice daily, PTX appears to reduce TNF-α expression and also reduces serum triglycerides in patients with AIDS [117] without causing significant gastrointestinal side effects that are seen when PTX is given at higher doses [118]. PTX also appears to reduce inflammation as measured by C-reactive protein [119-122] and oxidative stress as measured by serum malondialdehyde [123]. PTX improves endothelial function in animal models of hindlimb arterial ischemic injury, [124], possibly through potentiation of nitric oxide production in vascular smooth muscle cells [125, 126]. In the only previous clinical study evaluating PTX on FMD in humans [127], 13 patients with Type II diabetes were given 8 weeks of PTX with subsequent reduction in TNF-α levels and modestly improved FMD.

In both trials, the subjects had not received cART for at least 6 months prior to study entry and had CD4 counts ≥ 350cells/µL. Each pilot trial lasted 8 weeks with FMD measured at week 0 (baseline), week 4, and week 8. Subjects were excluded if they had any other pro-inflammatory condition, vascular disease, diabetes mellitus, or hypertension. Other exclusion criteria included receipt of other anti-inflammatory medications or lipid-lowering agents. We enrolled a total of 20 subjects over a 10 month period into these two trials. All laboratory measures were performed within one hour of starting the brachial artery reactivity testing. The Indiana University IRB fully approved these studies and stated that investigational new drug applications for these two products were not required.

Assuming any improvement in FMD with SSA would be dose-dependent, we gave 1500mg twice daily, the maximum FDA-approved dosage, of SSA to 11 subjects [97]. Two subjects were removed from the study before week 4 due to symptomatic hepatic transaminase elevations. Two additional subjects had the dosage of salsalate reduced by 750mg at week 4 due to mild, asymptomatic transaminase increases and subsequently finished the 8 week trial. Another subject was lost to follow-up after the week 4 visit. There was a significant median absolute increase in FMD of 4.2% (p=0.02) in the 8 subjects who completed the trial (Figure 2).The median FMD at week 8 (8.2%) is considered normal. NTGMD was normal at baseline (18%) and did not change over the course of the trial, suggesting that the ability of the arterial wall to dilate in response to a nitric oxide donor remained intact.

Several peripheral blood markers of inflammation and endothelial activation were measured using a customized commercial multiplex assay (SearchLight Human Analytes, Endogen, Rockford, IL). In the salsalate trial, we did not find any significant changes in the circulating inflammatory markers hsCRP, IL-6, MCP-1, sVCAM-1, sICAM-1, sTNFR1, sTNFR2, TNF-α, MMP-9, TIMP-1, PAI-1, SDF1b, IL-1ra, or IP-10. There was a significant decline in the endothelial activation markers vWF and albuminuria (both p=0.02) with salsalate. There were no significant changes in blood pressure, insulin sensitivity, lipid fraction, or HIV-1 RNA level.

Figure 2. Effects of salsalate and pentoxifylline on FMD in HIV-infected subjects not receiving cART.

**
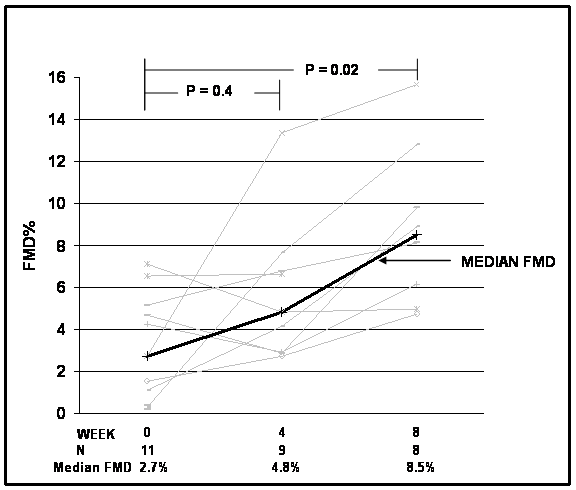
**

Salsalate

**
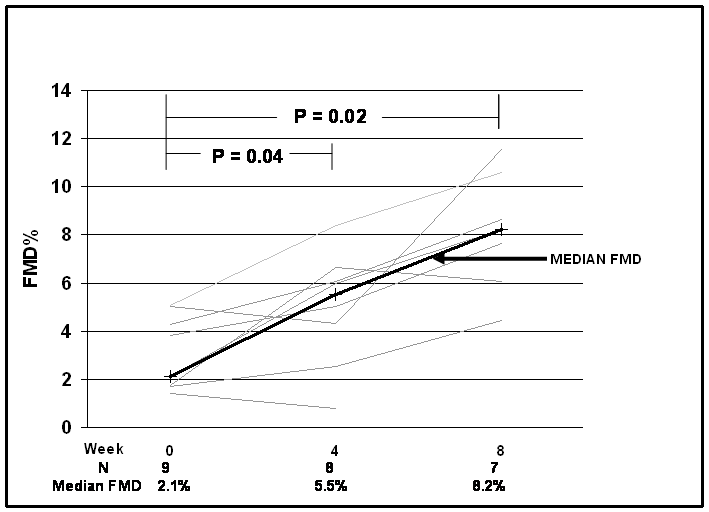
**

Pentoxifylline

PTX 400mg three times per day, was given to 9 subjects [128]. Two subjects were removed from study at weeks 2 and 5 due to initiation of prohibited concomitant drugs (inhaled steroid for bronchitis and cART for development of Kaposi Sarcoma, respectively) from their primary caregivers. FMD significantly improved after 4 weeks [median absolute increase 1.5%; p=0.04] and improved further at 8 weeks [median absolute increase from baseline 4.4%; p=0.02]. This degree of improvement in FMD has been associated with reduced risk of future cardiovascular events in the general population [21, 22]. Similar to what was observed with salsalate, 8 weeks of PTX resulted in normalization of FMD. Median NTGMD was 16% at entry and did not change over the course of the trial. It should be noted that the magnitude of improvement in FMD was similar regardless of initial FMD. . Looser stools were noticed by most of the subjects, but in none was this considered to be treatment-limiting. There were no drug-related laboratory toxicities.

We also observed in this pilot study a non-significant decline in triglycerides (p=0.09). No other lipid or metabolic parameter changed with PTX. Viral load did not change. Using an in-house HPLC assay, median plasma PTX concentrations were measured at weeks 4 and 8. The median PTX levels at weeks 4 and 8 were 73 ng/mL (270 nM) and 97 ng/mL (340 nM), respectively. There were no correlations between PTX concentrations and FMD response, which may reflect the small sample size of the study or that there is not a dose-response relationship between plasma PTX levels and FMD. We cannot rule out the possibility that intracellular levels of PTX may correlate with FMD response.

We examined the same peripheral blood markers of various potential inflammatory pathways as in the salsalate trial in order to gain insight into the pathways that may be responsible for the improvement in FMD with PTX. Because of the limited power in this pilot trial, we show in Table 1 those markers that we considered to have changed substantially, defined as either a change > 15% or a p-value < 0.05. Data are presented as median (range) due to small sample size.

Table 1. Markers of pathways that were affected by PTX.

| Biomarker* | Mechanistic Pathway | **HIV-infected Subjects Who Completed the 8 week trial of PTX (N=7)** | | |
| --- | --- | --- | --- | --- |
|  |  | Baseline  Median, pg/mL | % Change  (median) | P-value |
| MMP-9 | Induces vascular matrix degradation and promotes arterial stiffness | 28,713 | -22% | 0.68 |
| TIMP-1 | MMP inhibitor that is upregulated as a result of MMP elevation with higher levels predicting cardiovascular events | 151,500 | -17% | 0.46 |
| PAI-1 active | Inhibits fibrinolysis and promotes vascular thrombosis | 2,737 | -16% | 0.68 |
| hsCRP | General marker of acute phase inflammation | 606,800 | -24% | 0.8 |
| IP-10 (CCL10) | Promotes inflammatory cell recruitment | 124 | -28% | 0.02 |
| sVCAM-1 | Leukocyte cellular adhesion to the endothelium | 1,190,900 | -9% | 0.05 |

*MMP-9=matrix metalloproteinase 9; PAI-1=plasminogen activator inhibitor-1; TIMP-1=tissue inhibitor of metalloproteinase-1; SDF1b=stromal cell-derived factor 1b; IL-1ra=interleukin-1 receptor antagonist; IP-10=interferon gamma-inducible protein 10; hsCRP=highly sensitive C-reactive protein; sVCAM-1=soluble vascular cell adhesion molecule-1

These results suggest that anti-inflammatory drugs can improve endothelial function in HIV-infected patients not receiving cART. Salsalate, although effective, may be too unsafe to pursue in further trials. PTX improved FMD significantly and safely. The circulating biomarker data suggest that multiple inflammatory pathways may be inhibited by PTX. Based on the degree of statistical significance in the changes of these inflammatory biomarkers, it appears that the leukocyte adhesion pathway may be the most likely to be affected by treatment with pentoxifylline. Because cART only partially reduces sVCAM-1, adding PTX may provide additive benefits on inhibiting leukocyte adhesion with further improvements in endothelial function.

The reduction in the circulating markers of IP-10 and VCAM-1, although significant, appears relatively modest compared to the nearly three-fold improvement in FMD. It is possible that the inhibition of the arterial matrix degradation and vascular coagulation pathways ma be also significantly reduced with PTX. . Because of the reduction in triglycerides in this pilot study, we cannot exclude the possibility that improvement of dyslipidemia may also play a role in the beneficial effects of PTX on FMD. It should be noted that the pre-PTX plasma levels of circulating TNF-α in these subjects with higher CD4 cell counts (less than the detection limit of 4.7 pg/mL in 6 of 7 subjects) were much lower compared to those typically found in patients with AIDS [129]. PTX obviously did not further reduce these levels. If circulating TNF-α was the cause of endothelial inflammation and subsequent leukocyte adhesion, we should have observed higher pre-PTX levels with subsequent reduction with PTX. Therefore, PTX likely does not reduce endothelial inflammation by inhibiting production of TNF-α by circulating mononuclear cells in HIV-infected patients. An alternative explanation is that PTX has direct effects on the endothelial cell, perhaps by inhibiting NF-κB inhibition [112, 113] or reducing oxidative stress [123].

Given the strength of the evidence in the literature of the critical role also played by MCP-1 in the vascular lesions found in HIV-infected patients, we had were surprised that there were no significant changes in serum levels of MCP-1 with PTX (a modest median change of 1.9% over 8 weeks; p=1.0) despite having pre-PTX levels (median level 225 pg/mL) similar to those found in HIV-infected patients with atherosclerosis [77]. Although PTX may not affect MCP-1 production, one alternative explanation for these unexpected results is that PTX does blocks MCP-1 production by the endothelial cell, and, at least for this marker, this reduction is not reflected by a reduction in circulating levels.

If PTX does have direct beneficial effects on the endothelial, then this may partly explain the pronounced improvement in FMD seen in this pilot trial. However, these effects may not be reflected by peripheral blood biomarkers. , Because we cannot feasibly obtain vascular tissue samples from HIV-infected subjects to test this hypothesis, a cellular model is required to investigate these various possibilities.

2.4 Pentoxifylline (Trental**®**)

2.4.1 Description (adapted from the package insert for Pentoxifylline)

Pentoxifylline extended-release tablets for oral administration contain 400mg of the active drug and the following inactive ingredients: D&C Red No. 27 Aluminum Lake or FD&C Red No. 3, hypromellose USP, magnesium stearate NF, polyethylene glycol NF, povidone USP, talc USP, titanium dioxide USP, and other ingredients in a controlled-release formulation. Pentoxifylline is a tri-substituted xanthine derivative designed chemically as 1-(5-oxohexyl)-3. 7-dimethylxanthine that, unlike theophylline, is a hemorrheologic agent, i.e. an agent that affects blood viscosity. Pentoxifylline is soluble in water and ethanol, and sparingly soluble in toluene. The molecular formula is C_13_H_18_N_4_O_3_ and its molecular weight is 278.31.

- - 1. Clinical Pharmacology

Mode of Action

Pentoxifylline and its metabolites improve the flow properties of blood by decreasing its viscosity. In patients with chronic peripheral arterial disease, this increases blood flow to the affected microcirculation and enhances tissue oxygenation. The precise mode of action of pentoxifylline and the sequence of events leading to clinical improvement are still to be defined. Pentoxifylline administration has been shown to produce dose-related hemorrheologic effects, lowering blood viscosity, and improving erythrocyte flexibility. Leukocyte properties of hemorrheologic importance have been modified in animal and *in* *vitro* human studies. Pentoxifylline has been shown to increase leukocyte deformability and to inhibit neutrophil adhesion and activation. Tissue oxygen levels have been shown to be significantly increased by therapeutic doses of pentoxifylline in patients with peripheral arterial disease.

Pharmacokinetics and Metabolism

After oral administration in aqueous solution pentoxifylline is almost completely absorbed. It undergoes a first-pass effect and the various metabolites appear in plasma very soon after dosing. Peak plasma levels of the parent compound and its metabolites are reached within 1 hour. The major metabolites are Metabolite I (1-[5-hydroxyhexyl]-3, 7-dimethylxanthine), and Metabolite V (1-[3-carboxypropyl]-3, 7-dimethylxanthine), and plasma levels of these metabolites are 5 and 8 times greater, respectively, than pentoxifylline. Following oral administration of aqueous solutions containing 100 to 400mg of pentoxifylline, the pharmacokinetics of the parent compound and Metabolite I are dose-related and not proportional (non-linear), with half-life and area under the blood-level time curve (AUC) increasing with dose. The elimination kinetics of Metabolite V are not dose-dependent. The apparent plasma half-life of pentoxifylline varies from 0.4 to 0.8 hours and the apparent plasma half-lives of its metabolites vary from 1 to 1.6 hours. There is no evidence of accumulation or enzyme induction (Cytochrome P450) following multiple oral doses.

Excretion is almost totally urinary; the main biotransformation product is Metabolite V. Essentially no parent drug is found in the urine. Despite large variations in plasma levels of parent compound and its metabolites, the urinary recovery of Metabolite V is consistent and shows dose proportionality. Less than 4% of the administered dose is recovered in feces. Food intake shortly before dosing delays absorption of an immediate-release dosage form but does not affect total absorption. The pharmacokinetics and metabolism of pentoxifylline have not been studied in patients with renal and/or hepatic dysfunction, but AUC was increased and elimination rate decreased in an older population (60-68 years) compared to younger individuals (22-30 years).

After administration of the 400 mg extended-release pentoxifylline tablet, plasma levels of the parent compound and its metabolites reach their maximum within 2 to 4 hours and remain constant over an extended period of time. Co administration of pentoxifylline tablets with meals resulted in an increase in mean C_max_ and AUC by about 28% and 13% for pentoxifylline, respectively, C_max_ for Metabolite 1 also increased by about 20%. The extended-release of pentoxifylline from the tablet eliminates peaks and troughs in plasma levels for improved gastrointestinal tolerance.

2.4.3 Indications and Usage

Pentoxifylline is indicated for the treatment of patients with intermittent claudication on the basis of chronic occlusive arterial disease of the limbs. Pentoxifylline can improve function and symptoms but is not intended to replace more definitive therapy, such as surgical bypass, or removal of arterial obstructions when treating peripheral vascular disease.

- - 1. Contraindications

Pentoxifylline should not be used in patients with recent cerebral and/or retinal hemorrhage or in patients who have previously exhibited intolerance to this product or methylxanthines such as caffeine, theophylline, and theobromine.

- - 1. Precautions
       1. General Precautions

Patients with chronic occlusive arterial disease of the limbs frequently show other manifestations of arteriosclerotic disease. Pentoxifylline has been used safely for treatment of peripheral arterial disease in patients with concurrent coronary artery and cerebrovascular diseases, but there have been occasional reports of angina, hypotension, and arrhythmia. Controlled trials do not show that pentoxifylline causes such adverse effects more often than placebo, but as it is a methylxanthine derivative, it is possible some individuals will experience such responses. Patients on warfarin should have more frequent monitoring of prothrombin times, while patients with other risk factors complicated by hemorrhage (e.g., recent surgery, peptic ulceration, cerebral and/or retinal bleeding) should have periodic examinations for bleeding including hematocrit and/or hemoglobin.

- - - 1. Drug Interactions

Although a causal relationship has not been established, there have been reports of bleeding and/or prolonged prothrombin time in patients treated with pentoxifylline with and without anticoagulants or platelet aggregation inhibitors. Patients on warfarin should have more frequent monitoring of prothrombin times, while patients with other risk factors complicated by hemorrhage (e.g. recent surgery, peptic ulceration) should have periodic examinations for bleeding including hematocrit and/or hemoglobin. Concomitant administration of pentoxifylline and theophylline-containing drugs leads to increased theophylline levels and theophylline toxicity in some individuals. Such patients should be closely monitored for signs of toxicity and have their theophylline dosage adjusted as necessary. Pentoxifylline has been used concurrently with antihypertensive drugs, beta blockers, digitalis, diuretics, antidiabetic agents, and antiarrhythmics, without observed problems. Small decreases in blood pressure have been observed in some patients treated with pentoxifylline; periodic systemic blood pressure monitoring is recommended for patients receiving concomitant antihypertensive therapy. If indicated, dosage of the antihypertensive agents should be reduced.

- - - 1. Carcinogenesis, Mutagenesis and Impairment of Fertility

Long-term studies of the carcinogenic potential of pentoxifylline were conducted in mice and rats by dietary administration of the drug at doses up to 450mg/kg (approximately 19 times the maximum recommended human daily dose (MRHD) in both species when based on body weight; 1.5 times the MRHD in the mouse and 3.3 times the MRHD in the rat when based on body surface area). In mice, the drug was administered for 18 months, whereas in rats, the drug was administered for 18 months followed by an additional 6 months without drug exposure. In the rat study, there was a statistically significant increase in benign mammary fibroadenomas in females of the 450mg/kg group. The relevance of this finding to human use is uncertain. Pentoxifylline was devoid of mutagenic activity in various strains of *Salmonella* (Ames test) and in cultured mammalian cells (unscheduled DNA synthesis test) when tested in the presence and absence of metabolic activation. It was also negative in the *in vivo* mouse micronucleus test.

- - - 1. Use in Pregnancy

Category C. Teratongenicity studies have been performed in rats and rabbits using oral doses up to 576 and 264 mg/kg, respectively. On a weight basis, these doses are 24 and 11 times the maximum recommended human daily dose (MRHD); on a body-surface-area basis, they are 4.2 and 3.5 times the MRHD. No evidence of fetal malformation was observed. Increased resorption was seen in rats of the 576 mg/kg group. There are no adequate and well controlled studies in pregnant women. Pentoxifylline should be used during pregnancy only if the potential benefit justifies the potential risk to the fetus.

- - - 1. Nursing Mothers

Pentoxifylline and its metabolites are excreted in human milk. Because of the potential for tumorigenicity shown for pentoxifylline in rats, a decision should be made whether to discontinue nursing or discontinue the drug, taking into account the importance of the drug to the mother.

- - 1. Adverse Reactions

Clinical trials were conducted using either extended-release pentoxifylline tablets for up to 60 weeks or immediate-release pentoxifylline capsules for up to 24 weeks. Dosage ranges in the tablet studies were 400 mg bid to tid and in the capsule studies, 200-400 mg tid. The incidence of adverse reactions was higher in the capsule studies (where dose related increases were seen in digestive and nervous system side effects) than in the tablet studies. Studies with the capsule include domestic experience, whereas studies with the extended-release tablets were conducted outside of the US. Few patients discontinued because of adverse effects.

Pentoxifylline has been marketed in Europe and elsewhere since 1972. The following have been reported spontaneously since marketing or occurred in other clinical trials with an incidence of less than 1%; the causal relationship was uncertain:

Cardiovascular-dyspnea, edema, hypotension.

Digestive-anorexia, cholecystitis, constipation, dry mouth/thirst.

Nervous-anxiety, confusion, depression, seizures.

Respiratory-epistaxis, flu-like symptoms, laryngitis, nasal congestion.

Skin and Appendages-brittle fingernails, pruritus, rash, urticaria, angioedema.

Special Senses-blurred vision, conjunctivitis, earache, scotoma.

Miscellaneous-bad taste, excessive salivation, leucopenia, malaise, sore throat/swollen neck glands, weight change.

A few rate events have been reported spontaneously worldwide since marketing in 1972. Although they occurred under circumstances in which a casual relationship with pentoxifylline could not be established, they are listed to serve as information for physicians: Cardiovascular-angina, arrhythmia, tachycardia, anaphylactoid reactions. Digestive-hepatitis, jaundice, increased liver enzymes; and Hemic and Lymphatic-decreased serum fibrinogen, pancytopenia, aplastic anemia, leukemia, purpura, thrombocytopenia.

2.4.7 Overdosage

Overdosage with pentoxifylline has been reported in pediatric patients and adults. Symptoms appear to dose related. A report from a poison control center on 44 patients taking overdoses of enteric-coated pentoxifylline tablets noted that symptoms usually occurred 4-5 hours after ingestion and lasted about 12 hours. The highest amount ingested was 80mg/kg; flushing, hypotension, convulsions, somnolence, loss of consciousness, fever, and agitation occurred. All patients recovered. In addition to symptomatic treatment and gastric lavage, special attention must be given to supporting respiration, maintaining systemic blood pressure, and controlling convulsions. Activated charcoal has been used to absorb pentoxifylline in patients who have overdosed.

2.4.8 Dosage and Administration

The usual dosage of pentoxifylline in extended-release tablet form is one tablet (400 mg) three times a day with meals. While the effect of pentoxifylline may be seen within 2 to 4 weeks, it is recommended that treatment be continued for at least 8 weeks. Efficacy has been demonstrated in double-blind clinical studies of 6 months duration.

Digestive and central nervous system side effects are dose related. If patients develop these effects it is recommended that the dosage be lowered to one tablet twice a day (800 mg/day). If side effects persist at

this lower dosage, the administration of pentoxifylline should be discontinued.

2.5 Study Rationale

Finding inexpensive and easily tolerated agents for the improvement of HIV-related endothelial dysfunction and cardiovascular disease risk is vitally important given the worldwide HIV epidemic that disproportionately affects those living in developing economies.

The literature strongly suggests that HIV is associated with an increased risk of cardiovascular disease. Untreated HIV is associated with an inflammatory vasculopathy comprised of increased leukocyte adhesion and upregulated MCP-1 and VCAM-1. Untreated HIV is also associated with increased serum levels of soluble VCAM-1 and impaired endothelial function. This suggests that inhibition in the leukocyte recruitment and adhesion pathway may improve endothelial function and potentially reduce the risk of future cardiovascular events.

In this trial, we will seek to confirm our preliminary results by conducting a placebo-controlled, randomized study of PTX given for 8 weeks in HIV-infected patients not requiring cART and with a CD4 cell count >350/µL (13 per group; total N=26). Because we wish to avoid the potential confounding effects of cART on FMD, choosing this CD4 count criterion will allow us to evaluate patients who do not require antiretroviral therapies per current treatment guidelines. We had considered performing a longer trial of PTX in this group of subjects to determine sustainability of FMD improvement. However, our primary goal is to determine if PTX truly improves FMD, which should be achieved with an eight week trial based on our pilot data. The less expensive eight week study period will also allow us to evaluate the efficacy of PTX without concern of a decline in CD4 counts over this short timeframe that would then require initiation of cART and contaminate our results. If PTX does indeed improve FMD over this short timeframe, then future studies of longer duration can be designed to determine the sustainability of endothelial function improvement with PTX. Moreover, these investigations may serve as a model for other chronic inflammatory conditions associated with increased cardiovascular risk (e.g. systemic lupus erythematosis, hepatitis C, diabetes, and chronic kidney disease).

3.0 STUDY DESIGN

3.1 Overview

The objectives for this study will be met by conducting a randomized, placebo-controlled trial to determine the effects of 8 weeks of PTX on FMD in HIV-infected subjects not requiring cART and without any other significant cardiovascular risk factors (see Schedule of Events below). Based on our pilot data, the eight week design will allow sufficient time to determine if PTX truly improves FMD while being short enough to avoid contamination of cART initiation in the event of a decline in CD4 cell count. The dose of 400mg three times per day is the same dose used in our pilot study.

A total of 26 subjects, 13 in each arm, will be randomized (see Sample Size Estimation below). Up to 50 potential subjects will be screened to achieve the target of 26 randomizations. Because our preliminary data suggest that the improvement in FMD with PTX does not depend on baseline FMD, we will not stratify or limit inclusion based on this latter variable.

There are no published data that suggest that remote use of antiretroviral therapy affects FMD in those not receiving cART. Thus, we will allow subjects to enroll if they have not received antiretrovirals for at least six months prior to screening as opposed to mandating that subjects be completely antiretroviral-naïve to be eligible. Such subjects are likely those who were given cART despite having higher CD4 counts and stopped due to side effects. The six month period was chosen to provide sufficient time to allow washout of their previous therapies and return to baseline immune and virologic states. This criterion matches what we used in our pilot experiments, will increase the potential subject pool, and will make the study more generalizable while not affecting the validity of the final results. We will adjust for previous cART use as a baseline variable in the final analyses.

We will also study peripheral blood markers of inflammation (sVCAM-1, IP-10) that were affected by PTX in our preliminary investigations. Other potential confounding variables, such as smoking status, insulin resistance, dyslipidemia, and waist-hip ratio, will also be measured and adjusted for statistically in the final analyses. Plasma, serum, and urine samples will also be frozen and archived at both the central repository as mandated under the RFA supporting this trial and at our site for future studies of interest.

3.2 Screening visits

If deemed eligible for screening, the subject will be approached (with permission/referral of the potential subject’s primary care provider) by either a study investigator or by an Infectious Diseases Research Clinic study nurse to enter the screening phase of the study. After written, informed consent is provided, a random study code number will be assigned to the subject to ensure confidentiality. This study code number will be used for laboratory transport and processing, result reporting, and data recording.

At this visit, the medical/medication history will be reviewed and a brief physical examination will be performed. Blood will be drawn to determine if the subject meets the laboratory eligibility criteria (see Eligibility Criteria). Urine pregnancy testing for female study volunteers of reproductive potential will also be performed. All screening tests will be performed at the Indiana Clinical Research Center. If the eligibility criteria are met, then the subjects will enter the clinical trial.

Because we recognize that many inflammatory markers have not been systematically studied in HIV-infected patients (e.g. IP-10), we will attempt to minimize variability in baseline levels of these markers by averaging results from samples taken at both screening and at baseline.

3.3 Main study visits

After the subject has entered the study, s/he will come to the Indiana Clinical Research Center for the initial Week 0 (Entry) visit within three weeks of the screening visit. The subject’s medical records will be re-examined for clinical variables of interest. Adherence to study drug will be assessed by pill count, three-day recall, and eventually by PTX level which will be measured in batch at the end of each trial. To assess the success of the blinding mechanism/allocation, both the subjects and study nurse will be asked if they thought the subject was randomized to either PTX or placebo at study closeout.

Vital signs and anthropometrics will be measured per AIDS Adult Clinical Trials Group recommendations (<http://aactg.s-3.com/members/download/other/metabolic/>). The subject will provide fasting, morning blood samples for markers of cardiovascular risk, inflammation, endothelial activation, immune activation, and metabolism. All specimens not required for real-time safety monitoring will be frozen and batch-tested at the end of these studies to reduce interassay variability. The lone exception will be the flow cytometry measurements for activated CD8 cells, which will necessarily be performed on fresh whole blood samples.

The subject will then undergo brachial artery reactivity testing as outlined by the International Brachial Artery Reactivity Task Force [130]. The procedure for measuring brachial reactivity will require one hour of the subject’s time. There will be no documentation on the ultrasound scans regarding the HIV status (HIV-positive for the trial participants vs. HIV-negative for the external control subjects) or the randomization assignment of the participants in the two trials. This will ensure that Dr. Gupta, who will assess all the ultrasound results, will be blinded to these two key variables. All members of the study team will be blinded to the drug assignment for those subjects in the clinical trials. Female participants of reproductive potential will have their main study visits during the same phase of their menstrual cycle to minimize confounding by hormonal changes that may affect FMD results [131, 132]).

As a safety measure, a peripheral IV heplock will then be placed at the IU ICRC to reduce the number of venous punctures required for the serial blood draws required to estimate insulin resistance. The heplock will also facilitate the infusion of IV fluids if NTG-induced hypotension occurs during the brachial artery reactivity testing. By performing just one venous puncture for placement of the IV heplock, the risks of bleeding, bruising, pain, and infection to the subject will be minimized; this will also reduce the risk of accidental occupational exposure of potentially infectious material to the study team. The heplock will then be removed after assurance of subject hemodynamic stability once brachial reactivity testing is completed.

Safety and adverse events will be assessed at each study visit. Study participants will be encourage to notify the study team in between these visits if symptoms occur that may be related to study drug. In the event of an adverse event possibly related to study drug, a full assessment (examination, laboratories) will be performed immediately. Study discontinuation will occur if a Grade 3 or 4 event (per Division of AIDS Toxicity Grading System <http://roc.s-3.com/members/download/adulttox.pdf>), serious adverse event, or hospitalization likely due to study drug occurs. For less severe events thought to be related to study drug, then PTX or placebo dosing will be reduced by one pill (e.g. 400mg of active agent) and symptoms, signs, and laboratories will be reassessed after one week. If the adverse event continues at the same severity or improves only modestly, then dosing will be reduced by one more pill and again the participant will be reassessed after one week. If the adverse event persists at this time, then the subject will be discontinued from the study. However, if symptoms resolve after dose reduction, then the subject will continue on study using the new dosage level. The subject, of course, will have the option of withdrawing from the study at any time. All subjects who stop study drug prematurely due to an adverse event will be followed weekly until resolution. Please see Human Subjects Protection for further details on safety monitoring.

3.4 Brachial artery reactivity indices

The brachial artery diameter will be measured according to established guidelines [130] at baseline and in response to two stimuli: (1) endothelium-dependent vasodilation due to increased blood flow, or Flow-Mediated Dilation (FMD), and (2) endothelium-independent vasodilation due to sublingual nitroglycerin, or Nitroglycerin-Mediated Dilation (NTGMD). The same operator, blinded to case/control status of the subjects, will perform all studies.

Brachial reactivity measurements are subject to considerable day-to-day variability. Variables that will transiently affect vasomotor function include environmental conditions, tobacco and alcohol use, diet, and medications [133]. Therefore, it is desirable to control these factors in order to obtain results that more accurately reflect the underlying condition being studied. Therefore, the room temperature will be controlled at 68-71°F. The subject will rest supine for 10 minutes prior to imaging. Subjects will be told to not use tobacco-containing products or eat or drink anything other than water for 12 hours prior to the study. Female subjects will first have urine pregnancy testing performed prior to the ultrasound tests; if the subject is found to be pregnant, the subject’s participation will end. If any of the systolic blood pressures measured on either of the main study visits is <90mm Hg, then the visit must be rescheduled.

A 10 mHZ linear array vascular probe for B-mode imaging will be used to visualize the brachial artery. Data will be stored on a compact disc (for primary data analysis); these ultrasound data will also be backed-up on magneto-optical disks and the IU server with password protection. ECG leads will be placed on the subject’s chest and automatic blood pressure cuff to left upper arm, set at q.15 minutes. A small forearm cuff is placed on the widest part of the subject’s proximal right forearm (approximately 1-2 cm distal to the antecubital fossa). The brachial artery will then be located with the image optimized for best resolution of all three layers of the anterior and posterior walls (with special emphasis on identifying the media-adventitia interface). Three consecutive cardiac cycles of B-mode images of the brachial artery to the disks will be acquired and stored.

After this baseline evaluation, the measurement of endothelium-dependent vasodilation, or FMD, due to increased blood flow will begin. The forearm pressure cuff will be inflated to 250 mmHg for 5 minutes. At that time, imaging will be switched to B-mode. At 60 and 90 seconds after cuff deflation, three consecutive cardiac cycles of B-mode images of the brachial artery to the disc will be acquired and stored. The greater of the two diameters measured at these time points will be used for FMD analysis.

After 15 minutes from time of cuff deflation, the measurement of endothelium-independent vasodilation, or NTGMD, will begin. As before, three consecutive cardiac cycles of B-mode images of the brachial artery stored to the disc. Blood pressure measurement will then be repeated as a new baseline. Nitroglycerin (400 mcg SL) will then be administered. Three minutes later, three consecutive cardiac cycles of B-mode of the brachial artery stored to the disc and backed-up to a magneto-optical disk and the IU server with password protection.

3.5 Study Duration and Subject Retention

The maximum study period for each subject will be 11 weeks (screening phase of 3 weeks and main visit phase of 8 weeks). In order to promote retention in the study, the subject participants will be financially compensated for each screening and main visit.

4.0 SELECTION AND ENROLLMENT CRITERIA

4.1 Inclusion Criteria

4.1.1 HIV-1 infection, documented by (1) any licensed rapid HIV test or HIV enzyme or chemiluminescence immunoassay (E/CIA) test kit at any time prior to study entry and confirmed by a licensed Western blot or a second antibody test by a method other than the initial rapid HIV and/or E/CIA, or (2) by two detectable HIV-1 antigens, or (3) two detectable plasma HIV-1 RNA viral loads.

- - 1. Age equal to or greater than 18 years.
    2. CD4 cell count > 350/µL at screening.
    3. No receipt of any antiretroviral therapies within the six months prior to screening.
    4. No anticipated need for any antiretroviral therapies during the course of this study, as determined by the principal investigator or by the subject’s HIV caregiver.

Note: There is no HIV-1 RNA level eligibility criterion.

4.2 Exclusion Criteria

- - 1. Inability to complete written, informed consent.
    2. Incarceration at the time of any screening or main study visit.
    3. Diagnosed vascular disease (history of angina pectoris, coronary disease, peripheral vascular disease, cerebrovascular disease, aortic aneurysm, or otherwise known atherosclerotic disease).
    4. Diagnosed disease or process, besides HIV infection, associated with increased systemic inflammation (including, but not limited to, systemic lupus erythematosis, inflammatory bowel diseases, other collagen vascular diseases).

Note: Hepatitis B or C co-infections are NOT exclusionary

- - 1. History of bleeding diathesis, gastrointestinal ulceration or bleeding, cerebrovascular aneurysm or bleeding, or retinal hemorrhage.
    2. Known or suspected malignancy requiring systemic treatment within six months of screening.
    3. History of ADA-defined diabetes mellitus [134]

Note: History of gestational diabetes is not exclusionary if the potential subject does not have current ADA-defined diabetes.

- - 1. History of migraine headaches.
    2. History of Raynaud’s phenomenon.
    3. History of cardiac arrhythmias or cardiomyopathy.
    4. History of hypothyroidism or hyperthyroidism, even if treated.
    5. Known allergy or intolerance to pentoxifylline or other methylxanthines (e.g. theophylline, caffeine, theobromine).

Note: Use of caffeinated products, except on the mornings of the study visits, is not exclusionary.

- - 1. Known allergy or intolerance to nitroglycerin.
    2. History of carotid bruits.
    3. Creatinine clearance < 50mL/min (using the Cockcroft-Gault equation, see Appendix 1) using a serum creatinine level measured within 28 days prior to screening or at screening.
    4. Hemoglobin < 9.0mg/dL within 28 days of screening or at screening.
    5. Alanine aminotransferase (ALT) level or aspartate aminotransferase (AST) > 3 times ULN within 28 days of screening or at screening.
    6. Total bilirubin > 2.5 times ULN within 28 days prior to screening or at screening.
    7. Fever, defined as T ≥ 38.0C within 48 hours prior to screening.

Note: Fever within 48 hours prior to each main study visit will require postponement of that study visit until the patient has defervesced (T < 38.0C) for at least 48 hours; fevers continuing past the allowed study visit timeframe will result in study discontinuation.

- - 1. Therapy for acute infection or other serious medical illnesses within 14 days prior to screening.

Note: Therapy for acute infection or other serious medical illnesses that overlaps with a main study visit will result in postponement of that study visit until the course of therapy is completed; postponement outside of the allowed study visit timeframe will result in study discontinuation.

- - 1. Pregnancy or breastfeeding during the course of the study.
    2. Hypotension, defined as systolic blood pressure < 90mmHg, at time of screening.

Note: Hypotension noted prior to brachial artery reactivity testing on each main study visit will result in study visit postponement of at least one day until systolic pressure is ≥ 90mmHg the morning of brachial reactivity testing; postponement outside of the allowed study visit timeframe will result in study discontinuation.

- - 1. Hypertension, defined as the receipt of any antihypertensive medication within 28 days prior to screening or systolic blood pressure > 160mmHg at screening.
    2. Receipt of anti-inflammatory agents (including, but not limited to, plaquenil, infliximab, etanercept, mycophenylate mofetil, sirolimus, tacrolimus, cyclosporine, pentoxifylline, thalidomide) within 28 days of screening.
    3. Receipt of investigational agents, cytotoxic chemotherapy, systemic or topical glucocorticoids (of any dose and including inhaled or nasal steroids), or anabolic steroids within 28 days of screening.

Note: Physiologic testosterone replacement therapy is not exclusionary.

- - 1. Receipt of lipid-lowering drugs (including prescription or over the counter ‘fish oil’ or omega-3 fatty acid supplements), aspirin, other NSAIDS, acetazolamide, anticoagulants, anticonvulsants, or thyroid replacements within 28 days prior to screening.
    2. Use of sildenafil, vardenafil, or tadalafil within 72 hours (before or after) of each main study visit.
    3. Active drug or alcohol use or dependence that, in the opinion of the investigator, would interfere with adherence to study requirements.

If subjects are excluded due to the above criteria, they may be approached again in the future or have their study visit rescheduled within the allowable timeframe if these criteria are no longer applicable. Co-enrollment into other studies will be permitted provided no drugs prohibited on this study will be given in the other study/ies and that the required blood draws do not exceed safe limits when combined with those for this study.

We are specifically excluding subjects with diabetes, hypertension, and dyslipidemia requiring therapy at screening in these two studies in order to better evaluate the role of HIV as the primary determinant of endothelial dysfunction. However, we will not discontinue study participation if these criteria develop during the course of these trials for two reasons. First, this would limit generalizability as the development of diabetes, hypertension, and severe dyslipidemia with antiretroviral therapy is common in the HIV-infected population. Second, it is possible that reduction of inflammation by PTX may theoretically prevent insulin resistance, hypertension, and hypertriglyceridemia, which are important endpoints to capture in these trials.

4.3 Co-enrollment Guidelines

Co-enrollment into other studies will be permitted provided no drugs prohibited on this study will be given in the other study/ies and that the required blood draws do not exceed safe limits when combined with those for this study.

1. STUDY TREATMENT

5.1 Regimen, Administration, and Duration

At study entry, the study pharmacist at the Indiana University Health Investigational Drug Services (at Indiana University Hospital) must receive a prescription from a member of the study team for pentoxifylline 400mg po tid. As 400mg tablets will be provided, this translates to one tablet orally thrice daily. PTX should be taken with meals. Each subject will take PTX for 8 weeks. If the subject’s final main study visit is delayed past 56 days after study entry, then the subject will continue to take study drug/placebo until the final study visit is completed.

Dosage may be reduced by 400mg decrements in the daily dosage in the event of symptoms likely associated with PTX (e.g. nausea, vomiting, dizziness).

- 1. Product Formulation and Preparation

Extended-release pentoxifylline and matching placebo will be provided by the Indiana University Investigational Drug Services. The PTX ER tablets will be supplied as over-encapsulated capsules and will provided with backfill with standardized qualification procedures per FDA regulations. Placebos will match the study drug for color, taste, texture, size, and smell. All study medications will be stored in bulk at the Research Pharmacy at Indiana University Hospital at room temperature 20-35°C (68-77°F). Both PTX and the placebos will be dispensed in a tight, light-resistant container.

- 1. Study Drug Accountability and Dispensing

The study pharmacist at the Indiana University Health Investigational Drug Services (at Indiana University Hospital) is required to maintain complete records of all study drug. Subjects must bring back to clinic all study drug at each visit to examine pill counts (if necessary) and review dosing.

- 1. Concomitant Medications

5.4.1 Prohibited Medications

- Aspirin or other non-steroidal anti-inflammatory drugs (NSAIDS)
  - Anti-inflammatory agents (including, but not limited to, plaquenil, infliximab, etanercept, mycophenylate mofetil, sirolimus, tacrolimus, cyclosporine, pentoxifylline, theophylline, thalidomide)
- HIV-1/2 antiretroviral therapies
- Investigational agents
- Cytotoxic chemotherapy
- Systemic or topical glucocorticoids
- Anabolic steroids (physiologic testosterone replacement therapy is not exclusionary)
- Lipid-lowering drugs, including fish oil and omega-3 fatty acids
- Blood pressure-lowering drugs
- Anticoagulants
- Thyroid replacements
- Sildenafil, vardenafil, or tadalafil within 72 hours (before or after) of each main study visit
  - 1. Precautionary Medications
- None

1. CLINICAL AND LABORATORY EVALUATIONS

6.1 Schedule of Events

| **Evaluations** | **Screening** | **Study Visits** | | |
| --- | --- | --- | --- | --- |
|  | **-21 to -7 Day(s)** | **Week 0/**  **Entry** | **Week 4**  **Days 28-42** | **Week 8/Closeout**  **Days 56-70** |
| **Documentation of HIV Status** | X |  |  |  |
| **Medical History** | X |  |  |  |
| **Medication History** | X |  |  |  |
| **Concomitant Medications** |  | X | X | X |
| **Pentoxifylline/Placebo Dosing Modifications** |  | Anytime after entry based on adverse side effects | | |
| **Family History** |  | X |  |  |
| **Smoking History** |  | X |  |  |
| **Brief Physical Examination** | X | X | X | X |
| **Signs & Symptoms** |  | X | X | X |
| **Diagnoses** |  | X | X | X |
| **Adherence Questionnaire** |  |  | X | X |
| **Assessment of effective blinding** |  |  |  | X |
| **Height** |  | X |  |  |
| **Weight** | X | X | X | X |
| **Vital Signs** | X | X | X | X |
| **Waist/Hip Circumferences** |  | X | X | X |
| **Serum Creatinine** | X | X | X | X |
| **Calculated Creatinine Clearance** | X | X | X | X |
| **Liver Function Tests** | X | X | X | X |
| **Urine Pregnancy Test** | X | Whenever pregnancy suspected | | |
| **Urine Analysis** |  | X | X | X |
| **Hematology** | X | X | X | X |
| **CD4/CD8 cell counts and percentages** | X | X | X | X |
| **HIV-1 RNA Level (viral load)** |  | X | X | X |
| **Hepatitis B/C Serologies** |  | X |  |  |
| **PTX Levels** |  | X | X | X |
| **Whole blood for Advanced Flow Cytometry for Immune Activation (CD8+/CD38+/HLA-DR+ cells)** |  | X |  | X |
| **Frozen Serum/Plasma/Urine for Glucose, Insulin, Lipids, Albuminuria, Inflammatory and Endothelial Activation Markers** | X | X | X | X |
| **Frozen Plasma, Serum, & Urine for Future Studies** |  | X | X | X |
| **Brachial Artery Reactivity Testing** |  | X | X | X |

- 1. Definitions for Schedule of Events – Timing of Evaluations

6.2.1 Screening Evaluations

There will be one screening visit to determine eligibility. This must be completed within 21 days of entry (Week 0/Day0 Main Study Visit).

6.2.2 Main Study Evaluations

There will be three main study visits where blood and urine samples will be obtained, anthropometrics performed, and brachial artery ultrasound measurements will be performed. The Week 4 visit will occur as near to Day 28 after entry as possible, but can occur as late as 42 days after entry. The Week 8 visit will occur as near to Day 56 after entry as possible, but can occur as late as 70 days after entry. Each subject will continue to take study drug/placebo until the final study visit is completed.

- - 1. Study Discontinuation Evaluations

Study subjects who complete the entry (Week 0/Day 0) procedures and have taken at least two weeks of study drug but thereafter notify the study team that they will terminate their involvement prior to the Week 4 visit will have their Week 4 evaluations performed as soon as possible after the time of notice of termination. Subjects who notify the study team that they will terminate their involvement prior to the Week 8 visit and have taken at least 6 weeks of study drug will have their study Week 8 evaluations performed as soon as possible after the time of notice of termination. Subjects who notify the study team that they will terminate their involvement within two weeks after the Week 0 or Week 4 visit evaluations have been completed will have no further evaluations performed.

6.3 Definitions for Schedule of Events – Special Instructions and Definitions of Evaluations

6.3.1 Documentation of HIV-1 Infection

HIV-1 infection, as documented by any licensed ELISA test kit and confirmed by Western blot, must be present in the source documentation at the first screening visit. HIV-1 culture, HIV-1 antigen, plasma HIV-1 RNA, or a second antibody test by a method other than ELISA is acceptable as an alternative confirmatory test.

- - 1. Medical History

A medical history must be present in the source documents. Record the following on CRFs:

- Birthdate
- Sex
- Patient’s self report of ethnicity and race
- Initial date of HIV infection documentation
- Route of HIV infection
- Baseline, nadir, and most recent CD4 cell count (including dates of these values)
- Baseline, peak, and most recent HIV-1 RNA level (including dates of these values)
- Family history of cardiovascular disease
- Smoking history
- Menstrual cycle history (date of onset of last menstrual period)

- - 1. Medication History

A medication history must be present in source documents. The following information will be recorded on the CRFs:

- Complete HIV treatment history, including start and stop dates of any antiretroviral medication (estimated if the exact dates cannot be obtained), immune-based therapy, or HIV-related vaccines, including blinded study medications
- Aspirin or other NSAID use within 28 days prior to study entry
- Immune-modulating drugs within 28 days prior to study entry
- Any other prescription medications, within 28 days prior to entry
- Any vaccinations, within 28 days prior to entry
  - 1. Concomitant Medications

All new and/or discontinued prescription medications taken since the last clinic visit will be recorded on CRFs with start and stop dates. It is critical to have complete information on off-protocol use of aspirin or other NSAIDS. It is also critical to document the date(s) of receipt of any vaccinations since the last clinic visit.

- - 1. Pentoxifylline Dosing Modifications

All modifications to pentoxifylline dosing including initial doses, subject-initiated and/or protocol-mandated interruptions, modifications, and permanent discontinuation of treatment will be recorded on the CRFs at each visit. Subject-initiated and protocol-mandated interruptions include both inadvertent and deliberate interruptions of pentoxifylline doses for > 4 days.

- - 1. Family History

A family history of cardiovascular disease must be present in the source documentation and recorded on CRFs at entry.

- - 1. Smoking History

The subject’s past and current tobacco use must be present in the source documentation and recorded on CRFs at entry.

- - 1. Clinical Assessments

6.3.8.1 Height

Height will be recorded on CRFs at entry using the instructions found in the Adult AIDS Clinical Trials Group Metabolic webpage <http://aactg.s-3.com/members/download/other/metabolic/H&Wmeasures.doc>

- - - 1. Weight

Weight will be recorded on CRFs at screening and at each main study visits using instructions found in the Adult AIDS Clinical Trials Group Metabolic webpage <http://aactg.s-3.com/members/download/other/metabolic/H&Wmeasures.doc>

- - - 1. Resting Blood Pressure

Blood pressure measurements will be recorded on the CRFs at screening and at each main study visits. Blood pressure measurements should be obtained prior to the brachial artery ultrasound measurements.

Blood pressure measurements should be performed manually using an anaeroid sphygmomanometer on the same arm throughout the study. The subject should first sit quietly for five minutes. With the elbow and forearm resting comfortably on a flat table, the blood pressure should then be measured. After two minutes, repeat blood pressure measurement in the same arm. After another two minutes, repeat blood pressure measurements again. Therefore, three blood pressure measurements are to be documented in the CRFs at each main study visit.

- - - 1. Resting Heartrate

Resting heartrate measurements will be recorded on the CRFs at each main study visit. Heartrate measurements should be obtained prior to the brachial artery ultrasound measurements. Heartrates should be measured manually over the entire 60-second period. This may be done prior to the first blood pressure measurement. The subject should first sit quietly for five minutes.

- - - 1. Waist and Hip Measurements

Waist and hip circumferences will be measured at the time of each main study visit and recorded in the CRFs. These measurements will be made in triplicate according to the instructions found in the Adult AIDS Clinical Trials Group Metabolic webpage <http://aactg.s-3.com/members/download/other/metabolic/CIRCmeasures.doc>

- - - 1. Brief Physical Examination

The following assessments will be performed at screening:

- Auscultation of heart for signs of cardiac arrhythmias and valvular murmurs
- Auscultation of carotid arteries for bruits

The following assessments will be performed at each main study visit:

- Examination of the heart for arrhythmias
- Auscultation of the skin for rash or jaundice
  - Query of subject to ascertain whether s/he has experienced symptoms typically associated with pentoxifylline (abdominal discomfort, belching/flatus/bloating, dyspepsia, nausea/vomiting, flushing, dizziness, blurred vision)

These evaluations will be supplemented by additional features based on signs or symptoms that the subject experienced since the last study visit. All findings will be recorded on CRFs at screening or at each main study visit.

- - - 1. Signs and Symptoms

All signs and symptoms must be documented in the subject’s record. All signs and symptoms occurring within 30 days of entry and/or since the last study visit, regardless of grade, must be recorded on CRFs.

6.3.8.8 Diagnoses

All confirmed and probable diagnoses made since the last visit will be recorded on the CRFs, including current status at the time of the study visit.

6.3.8.9 Adherence Assessments

Subjects will be asked to bring their pill bottles with them at each visit so that a pill count can be estimates. They will also be asked to state how many doses of pentoxifylline were taken over the three days prior to the study visit. This information will be recorded on the CRFs.

6.3.8.10 Assessment of Successful Blinding

Once all study procedures have been completed, at the closeout visit each participant will be asked if s/he believed that each had received either pentoxifylline vs. placebo. The study nurse for each participant will also be asked this question. Answers will be recorded on CRFs for batched analysis at the end of the entire trial to assess the success of the blinding strategies for this trial.

6.3.8.11 Laboratory Evaluations

All screening and main study visit laboratory evaluations performed as part of this study must be present in source documentation. At entry, record all laboratory values on CRFs. For post-entry assessments, record on CRFs all Grade ≥ 2 laboratory toxicities and any laboratory toxicities that led to a change in pentoxifylline dosing regardless of grade. In addition, record on CRFs all values for hemoglobin, serum creatinine/calculated creatinine clearance, liver function tests, urine analysis, urine pregnancy tests, hepatitis B and C serologies, CD4/CD8 cell counts, and HIV-1 RNA levels. Refer to the Division of AIDS Table for Grading Adult Adverse Experiences on the Regulatory Compliance Center Web page: <http://rcc.tech-res-intl.com/>

Screening visit and main study visit safety blood and urine laboratories will be sent to the Indiana University clinical laboratory for immediate processing and analysis; results will be entered on CRFs and forwarded to the Biostatistical Division for electronic storage. Frozen plasma, serum, and urine specimens for future use will be batch analyzed by the IU clinical laboratory or by Searchlight/Endogen Laboratories; these data will be electronically forwarded to the Biostatistical Division. Serum, plasma, and urine specimens will be processed, stored, and shipped to the Data Coordinating Center for the RFA funding this trial. Brachial ultrasound results will be entered on CRFs and sent to the Biostatistical Division. Computer labels will be generated and affixed to the appropriate specimen containers or brachial ultrasound discs and will include the specimen number, subject number, specimen date, and primary specimen type. Labels must be affixed prior to freezing the vials. Specimen collection, processing, and storage instructions are listed under section 6.3.8.12.

NOTE: The peripheral IV heplock used for both drawing blood samples and for infusion of fluids if necessary during the BART procedure should be placed on an extremity other than the arm used for the brachial artery measurements.

Hematology

- Hemoglobin
- White blood cell count (WBC)
- Differential WBC
- Absolute neutrophil count (ANC)
- Platelets

Liver function tests

- ALT (SGPT)
- AST (SGOT)
- Total bilirubin

Blood Chemistries

- Serum creatinine/estimated creatinine clearance
- Serum glucose, potassium
- Hepatitis B surface antigen, hepatitis B surface antibody, hepatitis B core IgM antibody
- Hepatitis C antibody

Urine Specimen

- Urine analysis (record pH, WBC, RBC, Hgb, protein, leukocyte esterase, nitrite)
- Urine pregnancy test

CD4/CD8 cell counts and percentages

Because of the diurnal variation in CD4 and CD8 cell counts, determinations for individual subjects should be obtained consistently in ether the morning or the afternoon throughout the study, if possible.

Each time a CD4/CD8 cell count measurement is obtained, a WBC and differential from a sample obtained at the same time must also be performed.

HIV-1 RNA Levels

- Roche Amplicor 2.0 PCR viral loads through the IU commercial laboratory

Activated CD8 Cell Measurements

Fresh whole blood collected in K_3_ EDTA VACUTAINER (lavender) tubes will be transported within two hours of collection to the laboratory of Dr. Homer Twigg at the Roudebush VA Hospital for advanced flow cytometry measurements to determine the proportion of activated CD8 cells for each subject.

6.3.8.12 Specimen Collection, Processing, Labeling, and Storage for Specimens for Future Analysis

Plasma

- Collection:
  - For plasma #1, collect ONE (1) 7.0-mL, purple-top EDTA tube, FILL COMPLETELY (for possible additional HIV-1 RNA PCR testing). Complete processing within 6 hours of collection.
  - For plasma #2, collect ONE (1) 7.0-mL, purple-top EDTA tube, FILL COMPLETELY. After collection of blood, gently invert the tube 10 to 15 times and keep upright at room temperature until centrifugation. Keep refrigerated at 4^o^C after draw if unable to centrifuge immediately.
  - For plasma #3, collect an additional ONE (1) 7.0-ml 3.2% buffered Sodium citrate (SCI) tubes, fill completely (for storage). Mix gently by inverting 8 times immediately after filling. Do not shake the tube as this will break down fibrinogen in the sample. COMPLETE PROCESSING W/IN 1 HR OF COLLECTION.
- Processing:
  - For plasma #1 tube, spin at 800 x *g* for 10 minutes at room temperature. Plasma should be carefully removed from each tube, placed in a sterile polypropylene conical centrifuge tube and centrifuged again (at 800 x *g*) at room temperature for 10 minutes to completely remove platelets and cell debris. Prepare at least 3 aliquots (each containing 1.0-mL) from each 7mL EDTA tube and freeze at -80°C.
  - For plasma #2 tube, spin 3000 x *g* for 10 minutes at 4^o^C. Prepare at least 3 total aliquots containing at least 1.0-mL of plasma each and freeze at −80°C within 4 hours of collection.
  - For plasma #3 tube, spin at 1500 x *g* for 15 minutes at room temperature. Prepare at least 3 total aliquots containing at least 1.0-ml of plasma each and freeze at –80ºC within 60 minutes of collection.
- Labeling: Label on each aliquot the protocol-subject number, date, specimen type “HIV-PLA”, “E-PLA”, and “S-PLA” for tube number 1, 2, and 3, respectively.

Serum

- Collection: Collect FOUR (4) 7.0-mL red-top tubes without additive (for glucose, insulin, lipids, inflammatory markers, pentoxifylline levels, and other future studies) at room temperature. Let blood clot 30 minutes at room temperature in vertical position.
- Processing:
  - Spin tubes 10 minutes at 1300 x *g* to separate serum within 1 hour of collection. Prepare at least ten total aliquots containing at least 1.0-mL of serum. Separated serum MUST be refrigerated until frozen. Freeze at -80ºC as soon as possible within 8 hours of collection. Refrigerate aliquots if freezing cannot be accomplished immediately after processing.
- Labeling: Label on each aliquot the protocol-subject number, date, and specimen type “SER”

# Urine

# Collection: Collect at least 15 mL of urine in a standard collection cup using clean catch technique.

# Processing: Prepare at least 4 total aliquots containing at least 2.0-mL in screw-top plastic vials and freeze at ­­‑80ºC.

# Labeling: Label on each aliquot the protocol-subject number, date, and specimen type “URI”

6.3.8.13 Brachial artery ultrasound measurements

All brachial artery ultrasound measurements must be recorded on CRFs. If any of the systolic blood pressures measured on either of the main study visits prior to brachial artery ultrasound measurements is <90mm Hg, then the visit must be rescheduled.

Perform the assessment in the morning to avoid adrenergic stimulation after an overnight fast (approximately 8 hours).

Subjects may not use tobacco-containing products or eat or drink anything other than water for 8 hours prior to the assessment and until it is completed. See section 3.4 for the ultrasound procedure.

1. ADVERSE EVENT MANAGEMENT

The management guidelines listed here are for the adverse events most likely to occur during this protocol. Grade III or IV abnormalities (defined using the Division of AIDS Table for Grading Adult Adverse Experiences) will prompt immediate discontinuation of the drug. For all toxicities that requires pentoxifylline to be permanently discontinued, relevant clinical and laboratory tests will be repeated as needed until there is final resolution or stabilization of the toxicity. Clinical management decisions will be made by the principal investigator in conjunction with the subject’s primary caregiver; care plans and outcomes must be included in the source documentation. Serious adverse events (SAEs) will be documented on CRFs and forwarded to the Indiana University IRB within 10 working days of the event.

7.1 Grade 1 or 2 Toxicity/Adverse Event

Subjects who develop a Grade 1 or 2 adverse event or toxicity (with the exception of drop in hemoglobin/bleeding, rise in LFTs, rise in serum creatinine/decrease in creatinine clearance, rash, and hypotension as addressed below) may continue on protocol. Subjects experiencing Grade 1 or 2 adverse events who choose to discontinue permanently PTX treatment should be encouraged to complete the premature treatment discontinuation evaluations.

7.2 Grade 3 or 4 Toxicity/Adverse Event

Subjects who develop a Grade 3 or 4 adverse event or toxicity will have PTX permanently discontinued. However, if the primary investigator or primary caregiver has compelling evidence that the adverse event is NOT due to PTX, dosing may continue. Subjects experiencing Grade 3 or 4 adverse events who choose to discontinue permanently pentoxifylline treatment should be followed weekly until resolution of the adverse event.

7.3 Pentoxifylline (PTX)

7.3.1 Drop in Hemoglobin/Bleeding Episode

Subjects must be educated about the possible occurrence of anemia and bleeding, including abdominal pain, hematochezia, melena, and sudden onset headache. If these symptoms/signs occur, then a hemoglobin level must be checked. A 2gm/dL or greater drop from baseline in hemoglobin or bleeding episode from any site will result in permanent discontinuation of PTX. However, if the primary investigator or primary caregiver has compelling evidence that the drop in hemoglobin or bleeding episode is NOT due to PTX (e.g. trauma), dosing may continue with no more than a one week hiatus at the discretion of the primary investigator or primary caregiver. Rescheduling of a main study visit, if necessary, will be at the discretion of the primary investigator.

7.3.2 Rise in Liver Function Tests

Subjects must be educated about the possible occurrence of hepatitis and its manifestations, including abdominal pain, nausea, vomiting, and jaundice. If these symptoms/signs occur, then liver function tests must be obtained. If a Grade 3 or 4 toxicity for ALT, AST, or total bilirubin is found either when obtained for symptoms occurrence or at the Week 4 visit, then PTX will be permanently discontinued. However, if the primary investigator or primary caregiver has compelling evidence that the rise in serum ALT is NOT due to PTX, dosing may continue with no more than a one week hiatus at the discretion of the primary investigator or primary caregiver. Rescheduling of a main study visit, if necessary, will be at the discretion of the primary investigator.

7.3.3 Elevation of Serum Creatinine/Decrease in Calculated Creatinine Clearance

A decline in calculated creatinine clearance of > 25mL/min compared with the entry value or to an absolute value < 50mL/min that is confirmed (within one week of the original finding) and is not found to be reversible will result in permanent discontinuation of PTX. A urine analysis and CBC with differential should be obtained with the follow-up serum creatinine testing to assess for the possibility of nephritis (e.g. increase in urine WBCs and/or eosinophilia). However, if the primary investigator or primary caregiver has compelling evidence that the rise in serum ALT is NOT due to PTX (e.g. volume depletion/dehydration), dosing may continue with no more than a one week hiatus at the discretion of the primary investigator or primary caregiver. Rescheduling of a main study visit, if necessary, will be at the discretion of the primary investigator.

7.3.4 Rash

For a Grade 1 rash, management of PTX is at the discretion of the principal investigator and the primary caregiver.

For a Grade 2 rash, the principal investigator and primary caregiver will hold PTX until rash is resolved. At that time, the subject can be rechallenged at the same PTX dose. However, if the primary investigator or primary caregiver has compelling evidence that the rash is NOT due to PTX (e.g. sunburn), dosing may continue with no more than a one week hiatus at the discretion of the primary investigator or primary caregiver. Rescheduling of a main study visit, if necessary, will be at the discretion of the primary investigator.

If a Grade 2 rash recurs within one week, or if a Grade 3 or 4 rash occurs at any time, then PTX will be permanently discontinued.

7.3.5 Gastrointestinal

Gastrointestinal side effects from pentoxifylline appear to be dose-related. The development of nausea, vomiting, bloating, dyspepsia, or other gastrointestinal symptoms of any toxicity grade that is likely due to PTX will result in dosage reduction of PTX to 400mg po bid (one tablet twice daily instead of one tablet thrice daily). If this does not result in a reduction of symptoms of at least one toxicity grade, then PTX will be permanently discontinued.

7.3.6 Neurologic

Neurologic side effects from pentoxifylline appear to be dose-related. The development of dizziness, agitation/nervousness, blurred vision, or other neurologic symptoms of any toxicity grade that is likely due to PTX will result in dosage reduction of PTX to 400mg po bid (one tablet twice daily instead of one tablet thrice daily). If this does not result in a reduction of symptoms by at least one toxicity grade, then PTX will be permanently discontinued.

7.4 Nitroglycerin

7.4.1 Hypotension

The primary risk inherent with the administration of nitroglycerin as part of the brachial reactivity measurements is transient hypotension. Subjects will be questioned regarding the use of sildenafil, vardenafil, or tadalafil; if the subject used any of these agents within 72 hours before the ultrasound or intends to use any of these agents within 72 hours after the ultrasound, the visit will be rescheduled. To protect against the risks of nitroglycerin administration, trained medical personnel will monitor the subject’s blood pressure throughout brachial reactivity testing. If the subject becomes symptomatic (e.g. dizzy, perspiring, weak, or nauseated) or if the systolic blood pressure falls below 80mmHg, the study will be stopped immediately. The subject will be placed the Trendelenberg position, and if necessary, administered 0.9% NS (normal saline) through the previously placed IV heplock until the subject is stable.

Subjects who have an “excessive drop” in systolic blood pressure or have any other serious adverse event after receiving nitroglycerin will not undergo NTGMD as part of the brachial reactivity testing; however, FMD evaluations will still be performed. An “excessive drop” is defined as a decrease in systolic blood pressure to less than 80 mmHg, or a 30 mmHg or greater decrease from the pre-nitroglycerin systolic blood pressure.

- - 1. Headache

If the patient develops a headache that lasts for over an hour after the receipt of nitroglycerin, an analgesic will be recommended.

1. CRITERIA FOR STUDY DISCONTINUATION

- Request by the subject to withdraw
- Request of the primary care provider if s/he believes the study is no longer in the best interest of the subject
- If the subject is found to be pregnant or begins breastfeeding during the course of this study
- If the subject has develops fever, need for systemic therapy for acute or serious illness, or hypotension after screening that precludes completion of the main study visits within the allowed timeframes
- If the patient develops a need for antiretroviral therapy, as determined by the Principal Investigator or by the subject’s primary HIV caregiver
- Requirement for prohibited concomitant medication(s)
- Study drug-related toxicity (see section 7.0 Adverse Event Management)
- Clinical reasons believed life threatening by the physician
- Subject, as judged by the investigators, to be at risk of failing to comply with the provisions of the study protocol as to cause harm to self or interfere with the validity of the study results
- At the discretion of the funding agency

1. STATISTICAL CONSIDERATIONS AND DATA MANAGEMENT

9.1 General Considerations

For all analyses, an intention-to-treat (ITT) approach will be used. That is, study results will be analyzed by the treatment groups to which subjects are randomly assigned, regardless of compliance in taking the medication, adherence to the protocol, and completion of the study. This trial will be a double-blind, randomized, placebo-controlled, two parallel group study. Statistical significance will be considered if two-sided p-values are less than 5%. SAS version 9.1 will be used for all statistical analyses. The software PASS was used for sample size calculation.

9.2 Sample Size Justification

The primary endpoint is change in FMD from baseline to week 8. We used our preliminary data for estimating the detectable difference (Δ) and standard deviation for changes in FMD after 8 weeks with PTX. In the 7 subjects who completed the week 8 visit, an absolute mean (SD) improvement of 4.8% (1.34%) in the change in FMD at week 8 was observed. The 95% confidence interval was 3.5−6.0%. The correlation between week 0 FMD and week 8 FMD was 0.88. We used a very conservative approach, that is, the lower 95% confidence limit for a minimum detectable difference and assumed no change in FMD on average for the placebo group. We also used a conservative approach for estimating the standard deviation in the change in FMD. Our pilot study on 17 HIV positive patients not on cART showed a variability of 2.21 in baseline FMD. Assuming a correlation of 0.30 between week 0 and week8 FMD, we estimated the variability to be 2.62 for the change in FMD which is much higher than the variability we observed in the PTX study.

The sample size was determined based on a two-sample, independent, two-tailed t-test with 5% type I error. Samples sizes were adjusted for an estimated 20% dropout rate. A sample size of 10 per group will have at least 80% power to detect a difference in FMD of at least 3.5% assuming a common standard deviation of 2.62. To adjust for a possible 20% dropout rate, we will recruit 13 per group. The Figure below is a power curve demonstrating the ability to detect various magnitudes of treatment difference assuming a common SD of 2.62.

If a subject withdraws prior to study entry, this subject will be replaced. We expect up to 50 potential subjects will need to be screened to meet the target of 26 randomizations. If we find that the drop-out rate for those on study is greater than the anticipated 20%, then we will replace sufficient numbers of subjects so that the dropout rate is no longer greater than 20%.

9.3. Data Analysis

9.3.1 General Data Analysis

Continuous variables will be summarized by treatment groups using descriptive statistics (e.g., mean, standard deviation, minimum and maximum values). Categorical variables will be summarized using frequency counts and percentages. Baseline clinical and demographic data will be compared between two treatment groups to assess the effectiveness of the randomization. Dichotomous and ordinal variables will be examined using chi-square tests or Fisher’s exact test and continuous measures with Student’s t-tests. If some of the continuous variables are non-normal, a logarithmic or other appropriate transformation will be used to approximate a normal distribution in subsequent analyses that assume normality. Exploratory analyses will be performed to compute p-values which will be presented as an aid to evaluate the overall comparability of the treatment groups at baseline.

#### 9.3.2 Primary Endpoint Analysis

#### The primary outcome is the change in FMD at week 8 of post treatment from baseline. An analysis of covariance (ANCOVA) model will be used to compare the effect of intervention on this outcome variable. The model will include an indicator variable for intervention, baseline FMD, and other potential covariates, including CD4 cell count, HIV-1 RNA level (viral load), age, sex, race, and BMI. We will include covariates one at a time while keeping the intervention and baseline FMD. All covariates except baseline FMD will be excluded from the model if they have insignificant association with the change in FMD. If the response variable does not follow a normal distribution, an appropriate transformation will be used to approximate a normal distribution. The assumption of the constant variance will also be tested, and a transformation will be used if this assumption is violated.

9.3.3 Secondary endpoints analyses

The secondary endpoints are the change in FMD at week 4 and the changes in plasma levels of MCP-1, sVCAM-1, IP-10, fasting lipids, HOMA-IR, CD4 cell count, activated CD8 cell proportions, and HIV-1 RNA level at week 8 from baseline. The secondary analyses will be in exploratory nature and will primarily be used for hypothesis generation. All the secondary outcomes will be analyzed by the same statistical methods as for the primary endpoint. To analyze how the changes in secondary outcomes correlate with changes in FMD, Spearman Rank correlation will be used.

9.3.4 Randomization

26 HIV-infected patients not on cART will randomly be assigned to either the placebo or the pentoxifylline groups. The study statistician will generate a computerized randomization plan that will employ a certain block size for this trial. The block size will be known only to the statistician to maximize blinding for the subject and other study investigators.

9.4 Study Population

The subjects for these studies will be recruited from the HIV outpatient clinics at Indiana University and Wishard Hospitals. A survey of these clinics (total active clinic population of 2000 patients) suggest that at least 150 patients are not currently receiving cART and have CD4 counts >350cells/µL. Even accounting for other criteria that may exclude potential subjects, we expect that there should be no difficulties in enrolling the required 26 total subjects for this trial.

9.5 Study Duration

Based on the enrollments rates into our pilot trials, we expect to enroll 2 subjects per month. Therefore, the enrollment period will be approximately 13 months. The study period for each subject will be no more than 11 weeks (screening phase of 3 weeks and main visit phase of 8 weeks). Thus, the study durations will be 16 months. Including an additional 6 months to account for any unlikely difficulties in enrollment or trial procedures will raise this estimate to 22 months.

9.6 Data Management

A comprehensive web-based data management system will be developed for this study which will allow controlled entry through the internet. All data will be entered and stored in a password-protected Microsoft SQL Server 2005 database housed on a secured server separate from the web-server in the Division of Biostatistics. Server-side scripting hides the database calls from the web clients, obfuscating the data storage location.  An experienced Database Administrator provides database creation, daily backup, and installation of security patches.

A unique identifier will be assigned to each study participant and their associated study specimens. Patient identifiers will be located only within the subject’s study file in a separate, locked cabinet within the Infectious Diseases Research Clinic (IDRC) at University Hospital. Hardcopies of laboratory source records will also be stored in a locked file cabinet. All entryways to the IDRC are secured by padkey codes. Initial screening, consenting of potential subjects, and data abstraction and recording will be completed by the primary investigator with assistance from the research study nurse coordinators from the IDRC.

The data management system will facilitate quality control efforts, prevent entry of erroneous values, provide warnings for possible outliers, generate missing data reports, track subjects and samples, and assist in the preparation of data for statistical analysis. All data will be reviewed and processed through multiple verification and edit checking programs post entry to ensure data quality.

To facilitate data harmonization and ensure consistency with other research sites, every effort will be made to coordinate the collection and storage of data. The data managers will work with the Data Coordinating Center (DCC) to establish standards for terminology, data elements, and methodologies. Electronic data and research specimens will be provided to the DCC as needed.

1. HUMAN SUBJECTS RESEARCH AND PROTECTION

10.1 Investigator Training

All Indiana University personnel involved with this application have successfully completed the training and examination involved with the Indiana University Human Subjects Protection Course.

10.2 Risks to the Subjects

10.2.1 Human Subjects Involvement and Characteristics

- HIV-infected subjects will be recruited to participate in two randomized, placebo-controlled clinical trials investigating the efficacy of oral pentoxifylline to improve endothelial function.
  - Subjects must be at least 18 years of age, have documented HIV infection, and have not been hospitalized or untreated for severe illness/infection within 14 days prior to screening. A total of 26 subjects will be randomized.
  - The primary inclusion criteria are no receipt of cART within 6 months and no anticipated need for cART during the course of the trial. The chief exclusion criteria include known vascular disease, renal disease, diabetes, and treatment for hypertension or dyslipidemia at screening.
  - Potential subjects will be recruited from the Indiana University Hospital and Wishard Hospital infectious diseases outpatient clinics (both hospitals are affiliated with the Indiana University School of Medicine in Indianapolis). All procedures will occur in the Indiana Clinical Research Center at Indiana University Hospital.
- The only potential vulnerable subject population that may be included for these trials include children of at least 18 years of age.

10.2.2 Sources of Materials

- All data for this study will be obtained only after informed consent is provided by each subject. Existing medical records will be reviewed for demographics, medical diagnoses, and medications. Urine samples will be obtained for assessment of pregnancy (females only), dipstick measurements, and quantitative proteinuria/albuminuria levels. Serum/plasma samples will be obtained for testing for renal function, fasting metabolic parameters, hepatitis B and C co-infection status, HIV-1 RNA levels, CD4 cell counts, pentoxifylline levels, and inflammatory markers. Whole blood will also be collected for measurement of activated CD8 cells. Urine, serum, plasma, and cells will be obtained for deposition at the DCC and also at IU for future studies of interest. DNA will not be stored or analyzed for these studies. Ultrasound images from the brachial artery flow-mediated dilation testing will also be collected. Please see DATA SHARING PLAN for additional information regarding distribution of the unique datasets constructed from these studies.
- Results from pertinent medical records and procedures performed for these studies, as outlined above, will be recorded on the human subjects involved in the projects in this application.
- Data will be stored in a password-protected computerized database that will include only the subjects’ study identification number (names and other identifiable information will not be included). Therefore, the SID# will be the only link to the subject. Only the principal investigators, co-investigators, and research personnel who will directly obtain the necessary data will have access to the subject identities. All data obtained for this study will be obtained only after written, informed consent is provided by each subject.
- Records will be reviewed manually. Urine specimens will be obtained via standard clean-catch technique. Blood specimens will be obtained via peripheral venipuncture. Flow-mediated dilation of the brachial artery will be measured using high frequency ultrasound with images downloaded to a secure, encrypted electronic database. These data will be collected solely for the purpose of the proposed research projects.

10.2.3 Potential Risks

- There are minimal risks to the subjects enrolled in the proposed research. The first is the potential loss of subject confidentiality. The second consists of the risks associated with blood drawing/needle sticks, which include pain, bruising, infection, and phlebitis. The amounts of blood to be drawn at screening and at each main study visit are 15cc (one tablespoon) and 45cc (three tablespoons), respectively. The total amount of blood to be obtained would be approximately 150cc (10 tablespoons) over at least 11 weeks. The primary risks associated with the use of pentoxifylline are gastrointestinal (nausea, vomiting, dyspepsia), flushing, and dizziness. PTX is a Pregnancy Category C drug and is found in breastmilk. There are no known or potential interactions with antiretroviral medications or commonly used therapies in the HIV-infected population. Nevertheless, we will carefully monitor CD4 counts and HIV-1 viral loads to assess for any potential worsening of immunologic/virologic status for those randomized to PTX during these studies and will be evaluated carefully by the DSMB. The primary risks associated with nitroglycerin administration as part of the brachial artery testing are headache and transient hypotension. NTG may also infrequently cause the sensation of flushing of the skin and rash. There may also be moderate pain associated with the inflation of forearm cuff as part of the brachial reactivity testing.
- The principal alternative to these procedures would be not to participate in the research.

10.3 Adequacy of Protection Against Risks

10.3.1 Recruitment and Informed Consent

- Recruitment will only begin once the Indiana University Institutional Review Board has approved this study and advertisements to the community for the HIV-uninfected control participants. All subjects will be recruited from the outpatient HIV care clinics at Indiana University and Wishard Memorial Hospital. Self-referrals from other venues will also be considered. If the primary caregiver for the patient believes he or she is eligible for the study and allows the patient to be approached for screening, one of the study investigators or a study nurse will approach each potential participant during his or her regularly scheduled clinic visit. If eligibility is confirmed, then the purpose, procedures, and risks and benefits of the study will be discussed with the subject. Subjects will have ample opportunity to ask questions and to have all concerns addressed. If the subject wishes to pursue screening, then written informed consent will be obtained (and a copy given to the subject). All consent forms will be stored in a locked file cabinet.

10.3.2 Protection Against Risk

10.3.2.1 Confidentiality. To minimize the risk to subject confidentiality, patient identifiers will be removed once his or her data is abstracted and recorded, and only the random study identification number (generated when consent is provided) will be used. All hardcopy study data will be kept in a secured and locked file cabinet. All electronic data will be kept in a password-protected computer database. The only link between patient identifiers and the randomized study identification number will be kept in separate files. Identifiers will never be used in the analysis or presentation of study results.

10.3.2.2 Blood draws. The risks of blood drawing will be minimized by having only experienced medical personnel perform this procedure. The amount of blood that will be drawn falls well within safety standards for blood donation.

10.3.2.3 Pentoxifylline. Our pilot data and safety results from the literature suggest that PTX is very well-tolerated, so we do not expect any serious adverse events from use of this agent. However, to minimize potential risks with this drug, the eligibility criteria will reduce the potential adverse events associated with this drug by excluding those at higher risk. Adverse events will be monitored for closely. Safety laboratories (complete blood cell counts including platelet counts, chemistries, renal and liver function tests) will be obtained at each main study visit or upon development of symptoms. Grade III or IV abnormalities (defined using the Division of AIDS Table for Grading Adult Adverse Experiences) will prompt immediate discontinuation of the drug. Pregnant and breastfeeding women will be excluded. Women of reproductive age will be asked to use two forms of contraception during the clinical trials.

10.3.2.4 Nitroglycerin. Brachial artery reactivity testing will be performed in a controlled setting in the Indiana Clinical Research Center. To protect against the risks of nitroglycerin administration, registered nurses in the ICRC will monitor the subject’s blood pressure throughout brachial reactivity testing. Subjects with inherent resting hypotension will be excluded from the NTG-mediated flow dilation portion of the brachial reactivity testing. If the subject becomes symptomatic (e.g. dizzy, perspiring, weak or nauseated, or if the systolic blood pressure falls below 80mmHg or more then 30mmHg below their baseline systolic blood pressure, the study will be stopped immediately). The patient will be placed in Trendelenberg position and be given fluids through the previously placed IV heplock if the blood pressure does not recover spontaneously. If the patient develops a headache, an analgesic will be recommended.

10.3.2.5 Withholding Antiretroviral Therapies. Subjects will be included if their HIV caregiver has decided that antiretroviral therapy is not required and thus has been free of cART for at least six months time. There is a minimal risk to the subjects by continuing to refrain from use of antiretrovirals during the short course of this trial (maximum 11 weeks from screening to last main visit). To reduce this small risk, subjects must have CD4 cell counts above 350/µL, which is the level that the Department of Health and Human Services HIV Management Guidelines (<http://aidsinfo.nih.gov/contentfiles/AdultandAdolescentGL.pdf>) states that initiation of ART is not required. Per these guidelines, CD4 cell counts need only be routinely monitored every 3-4 months (12-16 weeks) in the untreated, asymptomatic patient. Therefore, the study course for each subject should be completed by the time their CD4 cell count would normally be rechecked in the clinic. However, if the subject develops signs, symptoms, or conditions associated with a reduction in CD4 cell count, the subject will have their CD4 cell count checked immediately. The principal investigator and the primary caregiver will then decide if antiretroviral therapies should be started immediately with permanent discontinuation of this study.

- - - 1. Adverse Event Management and Reporting. In the event of an adverse event, necessary medical and professional intervention will be provided immediately and billed to the subject’s medical insurance (if available). If the subject does not have insurance, care will be provided via the indigent care program at Wishard Memorial hospital. Standard procedures for reporting deviations from protocols and serious adverse events will be reported to the Indiana University IRB within 30 days (standard protocol). Deaths will be reported within 3 days. All adverse events will be graded using The Division of AIDS Table for Grading Adult Adverse Experiences is located at: <http://roc.s-3.com/members/download/adulttox.pdf>.
      2. Data and Safety Monitoring Committee. Dr. Allon Friedman (Indiana University Division of Nephrology) will serve as the Chair of the independent Data and Safety Monitoring Board for investigations proposed here. Drs. Gupta and Clauss (as co-PIs on this application) and Dr. Liu (as protocol statistician) will also be members of this board.
  1. Potential Benefits of the Proposed Research to the Subjects and Others

Potential benefits include an evaluation of their cardiovascular and immunologic status. They may also derive short-term benefits from the provision of pentoxifylline, although this is not guaranteed. Additionally, the nominal reimbursement for the subject’s time and effort may provide valuable resources for both the subject and his/her dependents. Finally, the subjects may also benefit from knowing that their participation will accrue knowledge that could benefit other HIV-infected patients.

Although there are no guaranteed clinical benefits from those who are randomized to treatment with pentoxifylline, this agent appears quite safe when used in HIV-infected subjects. The standard of care will not be altered in the control subjects. Therefore, the ancillary benefits to the participants in the proposed studies significantly outweigh the minimal risks in this study. Moreover, the proposed research may lead to other prevention and therapeutic studies that would demonstrate how to improve endothelial function and reduce future cardiovascular events in the HIV-infected population. This would benefit society directly by impacting clinical practice.

10.5 Importance of the Knowledge to be Gained

The knowledge that will be gained from this study will determine the relationships between HIV, inflammation, and endothelial dysfunction. This would potentially impact the clinical care of HIV-infected patients at risk for cardiovascular disease. Furthermore, prevention and therapeutic strategies (including the use of the inexpensive agent pentoxifylline) for these highly prevalent diseases can then be formulated, thereby reducing morbidity, mortality, and cost to the patients and society in general.

Again, the risks to the subjects are considered minimal. Even if the results are negative, the results of these investigations will add substantially to our knowledge on the mechanisms underlying endothelial dysfunction in HIV-infected patients. Therefore, the importance of the knowledge gained outweighs the risks to the participants.

10.6 Data and Safety Monitoring Plan

Progress of these studies, including data monitoring, subject enrollment, protocol deviations, and all SAE, will be reviewed by a panel including the PIs (Drs. Gupta and Clauss), the study statistician (Dr. Liu), and an expert investigator at Indiana University not directly connected with this study (Dr. Allon Friedman; Division of Nephrology, Indiana University). Reports, which will include descriptions of all adverse events, will be prepared for review by this panel every 6 months. Any study prematurely terminated due to an adverse event will be reviewed immediately. Standard procedures for reporting deviations from protocols to the I CRC, IRB, and NHLBI will be implemented. Serious Adverse Events (SAEs) will also be reported to the IRB within 3 working days and subsequently forwarded to NHLBI as required.

10.7 Inclusion of Women and Minorities

There are no exclusion criteria based on gender, racial category, or ethnicity in the proposed studies. Please see the attached Targeted/Planned Enrollment Table. Based on our previous cumulative experience and the general HIV-infected population cared for at the study sites at Indiana University, it is anticipated that approximately 25% of the study subjects will be women. Pregnant women will be excluded due to the confounding effects of pregnancy on cardiovascular physiology and because pentoxifylline is a Pregnancy Category C agent; we will ask all women of reproductive potential to use two forms of contraception during study participation. Breastfeeding women will also be excluded due to the presence of PTX in the breastmilk of women using pentoxifylline.

It is anticipated that approximately 40% and 8% of the study subjects will be black and Hispanic, respectively. American Indians, Alaskan Natives, Asians, Native Hawaiians or Other Pacific Islanders are not expected to be represented in the proposed study population due to extremely low representation of these groups within the Indiana University Medical Center outpatient clinics and in Indianapolis in general.

10.8 Inclusion of Children

Subjects of age 18 or greater will be included in this study. HIV-infected patients of age less than 18 years will be excluded due to the varying effects of puberty on metabolic parameters, inflammatory processes, and endothelial function (the primary outcome measure in the clinical trials proposed in this application).

11.0 REFERENCES

1. Healy B. **Endothelial cell dysfunction: an emerging endocrinopathy linked to coronary disease.** *J Am Coll Cardiol* 1990, **16**:357-358.

2. Vane JR, Anggard EE, Botting RM. **Regulatory functions of the vascular endothelium**. *N Engl J Med* 1990, **323**:27-36.

3. Egashira K. **Clinical importance of endothelial function in arteriosclerosis and ischemic heart disease**. *Circulation Journal* 2002, **66**:529-533.

4. Celermajer DS, Sorensen KE, Gooch VM, Spiegelhalter DJ, Miller OI, Sullivan ID*, et al.* **Non-invasive detection of endothelial dysfunction in children and adults at risk of atherosclerosis**. *Lancet* 1992, **340**:1111-1115.

5. Takase B, Uehata A, Akima T, Nagai T, Nishioka T, Hamabe A*, et al.* **Endothelium-dependent flow-mediated vasodilation in coronary and brachial arteries in suspected coronary artery disease**. *Am J Cardiol* 1998, **82**:1535-1539, A1537-1538.

6. Anderson TJ, Uehata A, Gerhard MD, Meredith IT, Knab S, Delagrange D*, et al.* **Close relation of endothelial function in the human coronary and peripheral circulations**. *J Am Coll Cardiol* 1995, **26**:1235-1241.

7. Vogel RA. **Measurement of endothelial function by brachial artery flow-mediated vasodilation**. *Am J Cardiol* 2001, **88**:31E-34E.

8. Ross R. **The pathogenesis of atherosclerosis--an update**. *N Engl J Med* 1986, **314**:488-500.

9. Celermajer DS, Sorensen KE, Bull C, Robinson J, Deanfield JE. **Endothelium-dependent dilation in the systemic arteries of asymptomatic subjects relates to coronary risk factors and their interaction**. *J Am Coll Cardiol* 1994, **24**:1468-1474.

10. Benjamin EJ, Larson MG, Keyes MJ, Mitchell GF, Vasan RS, Keaney JF, Jr.*, et al.* **Clinical correlates and heritability of flow-mediated dilation in the community: the Framingham Heart Study**. *Circulation* 2004, **109**:613-619.

11. Vita JA, Keaney JF, Jr., Larson MG, Keyes MJ, Massaro JM, Lipinska I*, et al.* **Brachial artery vasodilator function and systemic inflammation in the Framingham Offspring Study**. *Circulation* 2004, **110**:3604-3609.

12. Gupta SK, Mather KJ, Agarwal R, Saha CK, Considine RV, Dube MP. **Proteinuria and endothelial dysfunction in stable HIV-infected patients. A pilot study**. *J Acquir Immune Defic Syndr* 2007, **45**:596-598.

13. Gokce N, Keaney JF, Jr., Hunter LM, Watkins MT, Nedeljkovic ZS, Menzoian JO*, et al.* **Predictive value of noninvasively determined endothelial dysfunction for long-term cardiovascular events in patients with peripheral vascular disease**. *J Am Coll Cardiol* 2003, **41**:1769-1775.

14. Perticone F, Ceravolo R, Pujia A, Ventura G, Iacopino S, Scozzafava A*, et al.* **Prognostic significance of endothelial dysfunction in hypertensive patients**. *Circulation* 2001, **104**:191-196.

15. Neunteufl T, Heher S, Katzenschlager R, Wolfl G, Kostner K, Maurer G*, et al.* **Late prognostic value of flow-mediated dilation in the brachial artery of patients with chest pain**. *Am J Cardiol* 2000, **86**:207-210.

16. Suwaidi JA, Hamasaki S, Higano ST, Nishimura RA, Holmes DR, Jr., Lerman A. **Long-term follow-up of patients with mild coronary artery disease and endothelial dysfunction**. *Circulation* 2000, **101**:948-954.

17. Chan SY, Mancini GBJ, Kuramoto L, Schulzer M, Frohlich J, Ignaszewski A. **The prognostic importance of endothelial dysfunction and carotid atheroma burden in patients with coronary artery disease**. *J Am Coll Cardiol* 2003, **42**:1037-1043.

18. Frick M, Suessenbacher A, Alber HF, Dichtl W, Ulmer H, Pachinger O*, et al.* **Prognostic value of brachial artery endothelial function and wall thickness**. *J Am Coll Cardiol* 2005, **46**:1006-1010.

19. Shimbo D, Grahame-Clarke C, Miyake Y, Rodriguez C, Sciacca R, Di Tullio M*, et al.* **The association between endothelial dysfunction and cardiovascular outcomes in a population-based multi-ethnic cohort**. *Atherosclerosis* 2006.

20. Corretti MC, Anderson TJ, Benjamin EJ, Celermajer D, Charbonneau F, Creager MA*, et al.* **Guidelines for the ultrasound assessment of endothelial-dependent flow-mediated vasodilation of the brachial artery: a report of the International Brachial Artery Reactivity Task Force**. *J Am Coll Cardiol* 2002, **39**:257-265.

21. Suessenbacher A, Frick M, Alber HF, Barbieri V, Pachinger O, Weidinger F. **Association of improvement of brachial artery flow-mediated vasodilation with cardiovascular events**. *Vasc Med* 2006, **11**:239-244.

22. Modena MG, Bonetti L, Coppi F, Bursi F, Rossi R. **Prognostic role of reversible endothelial dysfunction in hypertensive postmenopausal women**. *J Am Coll Cardiol* 2002, **40**:505-510.

23. Triant VA, Lee H, Hadigan C, Grinspoon SK. **Increased Acute Myocardial Infarction Rates and Cardiovascular Risk Factors Among Patients with HIV Disease**. *J Clin Endocrinol Metab* 2007.

24. Obel N, Thomsen HF, Kronborg G, Larsen CS, Hildebrandt PR, Sorensen HT*, et al.* **Ischemic heart disease in HIV-infected and HIV-uninfected individuals: a population-based cohort study**. *Clin Infect Dis* 2007, **44**:1625-1631.

25. Klein D, Hurley LB, Quesenberry CP, Jr., Sidney S. **Do protease inhibitors increase the risk for coronary heart disease in patients with HIV-1 infection?** *Journal of Acquired Immune Deficiency Syndromes: JAIDS* 2002, **30**:471-477.

26. Vittecoq D, Escaut L, Chironi G, Teicher E, Monsuez JJ, Andrejak M*, et al.* **Coronary heart disease in HIV-infected patients in the highly active antiretroviral treatment era**. *AIDS* 2003, **17**:S70-76.

27. Friis-Moller N, Sabin CA, Weber R, d'Arminio Monforte A, El-Sadr WM, Reiss P*, et al.* **Combination antiretroviral therapy and the risk of myocardial infarction**. *N Engl J Med* 2003, **349**:1993-2003.

28. Holmberg SD, Moorman AC, Williamson JM, Tong TC, Ward DJ, Wood KC*, et al.* **Protease inhibitors and cardiovascular outcomes in patients with HIV-1**. *Lancet* 2002, **360**:1747-1748.

29. d'Arminio A, Sabin CA, Phillips AN, Reiss P, Weber R, Kirk O*, et al.* **Cardio- and cerebrovascular events in HIV-infected persons**. *AIDS* 2004, **18**:1811-1817.

30. Mary-Krause M, Cotte L, Simon A, Partisani M, Costagliola D. **Increased risk of myocardial infarction with duration of protease inhibitor therapy in HIV-infected men**. *AIDS* 2003, **17**:2479-2486.

31. Friis-Moller N, Reiss P, Sabin CA, Weber R, Monforte A, El-Sadr W*, et al.* **Class of antiretroviral drugs and the risk of myocardial infarction**. *N Engl J Med* 2007, **356**:1723-1735.

32. El-Sadr WM, Lundgren JD, Neaton JD, Gordin F, Abrams D, Arduino RC*, et al.* **CD4+ count-guided interruption of antiretroviral treatment**. *N Engl J Med* 2006, **355**:2283-2296.

33. Phillips A, Carr A, Neuhaus J, Visnegarwala F, Prineas R, Burman W*, et al.* **Interruption of ART and Risk of Cardiovascular Disease: Findings from SMART**. *14th Conference on Retroviruses and Opportunistic Infections*. Los Angeles, CA 2007.

34. Cota-Gomez A, Flores NC, Cruz C, Casullo A, Aw TY, Ichikawa H*, et al.* **The human immunodeficiency virus-1 Tat protein activates human umbilical vein endothelial cell E-selectin expression via an NF-kappa B-dependent mechanism**. *J Biol Chem* 2002, **277**:14390-14399.

35. Andras IE, Pu H, Deli MA, Nath A, Hennig B, Toborek M. **HIV-1 Tat protein alters tight junction protein expression and distribution in cultured brain endothelial cells**. *J Neurosci Res* 2003, **74**:255-265.

36. Avraham HK, Jiang S, Lee TH, Prakash O, Avraham S. **HIV-1 Tat-mediated effects on focal adhesion assembly and permeability in brain microvascular endothelial cells**. *J Immunol* 2004, **173**:6228-6233.

37. Henderson WW, Ruhl R, Lewis P, Bentley M, Nelson JA, Moses AV. **Human immunodeficiency virus (HIV) type 1 Vpu induces the expression of CD40 in endothelial cells and regulates HIV-induced adhesion of B-lymphoma cells**. *J Virol* 2004, **78**:4408-4420.

38. Liu K, Chi DS, Li C, Hall HK, Milhorn DM, Krishnaswamy G. **HIV-1 Tat protein-induced VCAM-1 expression in human pulmonary artery endothelial cells and its signaling**. *Am J Physiol Lung Cell Mol Physiol* 2005, **289**:L252-260.

39. Matzen K, Dirkx AE, oude Egbrink MG, Speth C, Gotte M, Ascherl G*, et al.* **HIV-1 Tat increases the adhesion of monocytes and T-cells to the endothelium in vitro and in vivo: implications for AIDS-associated vasculopathy**. *Virus Res* 2004, **104**:145-155.

40. Shiu C, Barbier E, Di Cello F, Choi HJ, Stins M. **HIV-1 gp120 as well as alcohol affect blood-brain barrier permeability and stress fiber formation: involvement of reactive oxygen species**. *Alcohol Clin Exp Res* 2007, **31**:130-137.

41. Baliga RS, Chaves AA, Jing L, Ayers LW, Bauer JA. **Aids Related Vasculopathy: Evidence For Oxidative And Inflammatory Pathways In Murine And Human Aids**. *Am J Physiol Heart Circ Physiol* 2005.

42. Kanmogne GD, Primeaux C, Grammas P. **Induction of apoptosis and endothelin-1 secretion in primary human lung endothelial cells by HIV-1 gp120 proteins**. *Biochem Biophys Res Commun* 2005, **333**:1107-1115.

43. Laurence J, Mitra D, Steiner M, Staiano-Coico L, Jaffe E. **Plasma from patients with idiopathic and human immunodeficiency virus-associated thrombotic thrombocytopenic purpura induces apoptosis in microvascular endothelial cells**. *Blood* 1996, **87**:3245-3254.

44. Monsuez JJ, Dufaux J, Vittecoq D, Vicaut E. **Reduced reactive hyperemia in HIV-infected patients**. *Journal of Acquired Immune Deficiency Syndromes: JAIDS* 2000, **25**:434-442.

45. Rios Blanco JJ, Suarez Garcia I, Gomez Cerezo J, Pena Sanchez de Rivera JM, Moreno Anaya P, Garcia Raya P*, et al.* **Endothelial function in HIV-infected patients with low or mild cardiovascular risk**. *J Antimicrob Chemother* 2006.

46. Bonnet D, Aggoun Y, Szezepanski I, Bellal N, Blanche S. **Arterial stiffness and endothelial dysfunction in HIV-infected children**. *AIDS* 2004, **18**:1037-1041.

47. Charakida M, Donald AE, Green H, Storry C, Clapson M, Caslake M*, et al.* **Early structural and functional changes of the vasculature in HIV-infected children: impact of disease and antiretroviral therapy**. *Circulation* 2005, **112**:103-109.

48. Solages A, Vita JA, Thornton DJ, Murray J, Heeren T, Craven DE*, et al.* **Endothelial function in HIV-infected persons**. *Clin Infect Dis* 2006, **42**:1325-1332.

49. van Wijk JP, de Koning EJ, Cabezas MC, Joven J, op't Roodt J, Rabelink TJ*, et al.* **Functional and structural markers of atherosclerosis in human immunodeficiency virus-infected patients**. *J Am Coll Cardiol* 2006, **47**:1117-1123.

50. Stein JH, Klein MA, Bellehumeur JL, McBride PE, Wiebe DA, Otvos JD*, et al.* **Use of human immunodeficiency virus-1 protease inhibitors is associated with atherogenic lipoprotein changes and endothelial dysfunction**. *Circulation* 2001, **104**:257-262.

51. Shankar SS, Dube MP, Gorski JC, Klaunig JE, Steinberg HO. **Indinavir impairs endothelial function in healthy HIV-negative men**. *Am Heart J* 2005, **150**:933.

52. Conklin BS, Fu W, Lin PH, Lumsden AB, Yao Q, Chen C. **HIV protease inhibitor ritonavir decreases endothelium-dependent vasorelaxation and increases superoxide in porcine arteries**. *Cardiovasc Res* 2004, **63**:168-175.

53. Sutliff RL, Dikalov S, Weiss D, Parker J, Raidel S, Racine AK*, et al.* **Nucleoside reverse transcriptase inhibitors impair endothelium-dependent relaxation by increasing superoxide**. *Am J Physiol Heart Circ Physiol* 2002, **283**:H2363-2370.

54. Fu W, Chai H, Yao Q, Chen C. **Effects of HIV Protease Inhibitor Ritonavir on Vasomotor Function and Endothelial Nitric Oxide Synthase Expression**. *J Acquir Immune Defic Syndr* 2005, **39**:152-158.

55. Dube MP, Shen C, Greenwald ML, Mather K. **Effects of 4 weeks of atazanavir, lopinavir/ritonavir or placebo on endothelial function and insulin sensitivity in healthy men**. *Antiviral Therapy* 2007, **Supplement 2**:L14.

56. Grubb JR, Dejam A, Voell J, Blackwelder WC, Sklar PA, Kovacs JA*, et al.* **Lopinavir-ritonavir: effects on endothelial cell function in healthy subjects**. *J Infect Dis* 2006, **193**:1516-1519.

57. Torriani FJ, Komarow L, Cotter BR, Murphy RL, Fichtenbaum CJ, Currier JS*, et al.* **Control of HIV viral replication is associated with rapid improvement in endothelial function sustained over 24 weeks: A5152s, a substudy of A5142** *Antiviral Therapy* 2007, **12**:L15.

58. Wolf K, Tsakiris DA, Weber R, Erb P, Battegay M. **Antiretroviral therapy reduces markers of endothelial and coagulation activation in patients infected with human immunodeficiency virus type 1**. *J Infect Dis* 2002, **185**:456-462.

59. Jonasson L, Holm J, Skalli O, Bondjers G, Hansson GK. **Regional accumulations of T cells, macrophages, and smooth muscle cells in the human atherosclerotic plaque**. *Arteriosclerosis* 1986, **6**:131-138.

60. Hansson GK, Holm J, Jonasson L. **Detection of activated T lymphocytes in the human atherosclerotic plaque**. *Am J Pathol* 1989, **135**:169-175.

61. Frostegard J, Ulfgren AK, Nyberg P, Hedin U, Swedenborg J, Andersson U*, et al.* **Cytokine expression in advanced human atherosclerotic plaques: dominance of pro-inflammatory (Th1) and macrophage-stimulating cytokines**. *Atherosclerosis* 1999, **145**:33-43.

62. van der Wal AC, Becker AE, van der Loos CM, Das PK. **Site of intimal rupture or erosion of thrombosed coronary atherosclerotic plaques is characterized by an inflammatory process irrespective of the dominant plaque morphology.** *Circulation* 1994, **89**:36-44.

63. Kang SM, Chung N, Kim JY, Koo BK, Choi D, Jang Y*, et al.* **Relation of vasodilator response of the brachial artery to inflammatory markers in patients with coronary artery disease**. *Echocardiography* 2002, **19**:661-667.

64. Clapp BR, Hingorani AD, Kharbanda RK, Mohamed-Ali V, Stephens JW, Vallance P*, et al.* **Inflammation-induced endothelial dysfunction involves reduced nitric oxide bioavailability and increased oxidant stress**. *Cardiovasc Res* 2004, **64**:172-178.

65. Tonetti MS, D'Aiuto F, Nibali L, Donald A, Storry C, Parkar M*, et al.* **Treatment of periodontitis and endothelial function**. *N Engl J Med* 2007, **356**:911-920.

66. Smeeth L, Thomas SL, Hall AJ, Hubbard R, Farrington P, Vallance P. **Risk of myocardial infarction and stroke after acute infection or vaccination.** *N Engl J Med* 2004, **351**:2611-2618.

67. Aburawi E, Liuba P, Pesonen E, Yla-Herttuala S, Sjoblad S. **Acute respiratory viral infections aggravate arterial endothelial dysfunction in children with type 1 diabetes**. *Diabetes Care* 2004, **27**:2733-2735.

68. Charakida M, Donald AE, Terese M, Leary S, Halcox JP, Ness A*, et al.* **Endothelial dysfunction in childhood infection**. *Circulation* 2005, **111**:1660-1665.

69. Marchesi S, Lupattelli G, Lombardini R, Sensini A, Siepi D, Mannarino M*, et al.* **Acute inflammatory state during influenza infection and endothelial function**. *Atherosclerosis* 2005, **178**:345-350.

70. Tabib A, Greenland T, Mercier I, Loire R, Mornex JF. **Coronary lesions in young HIV-positive subjects at necropsy**. *Lancet* 1992, **340**:730.

71. Tabib A, Leroux C, Mornex JF, Loire R. **Accelerated coronary atherosclerosis and arteriosclerosis in young human-immunodeficiency-virus-positive patients**. *Coron Artery Dis* 2000, **11**:41-46.

72. Zietz C, Hotz B, Sturzl M, Rauch E, Penning R, Lohrs U. **Aortic endothelium in HIV-1 infection: chronic injury, activation, and increased leukocyte adherence**. *Am J Pathol* 1996, **149**:1887-1898.

73. Eugenin EA, Morgello S, Kotman ME, Mosoian A, Lento PA, Berman JW*, et al.* **Human immuodeficiency virus (HIV) infects human arterial smooth muscle cells in vivo and in vitro**. *Am J Pathol* 2008, **172**:1-12.

74. Cenacchi G, Re MC, Preda P, Pasquinelli G, Furlini G, Apkarian RP*, et al.* **Human immunodeficiency virus type-1 (HIV-1) infection of endothelial cells in vitro: a virological, ultrastructural and immuno-cytochemical approach**. *J Submicrosc Cytol Pathol* 1992, **24**:155-161.

75. Conaldi PG, Serra C, Dolei A, Basolo F, Falcone V, Mariani G*, et al.* **Productive HIV-1 infection of human vascular endothelial cells requires cell proliferation and is stimulated by combined treatment with interleukin-1 beta plus tumor necrosis factor-alpha**. *J Med Virol* 1995, **47**:355-363.

76. Joven J, Coll B, Tous M, Ferre N, Alonso-Villaverde C, Parra S*, et al.* **The influence of HIV infection on the correlation between plasma concentrations of monocyte chemoattractant protein-1 and carotid atherosclerosis**. *Clin Chim Acta* 2006, **368**:114-119.

77. Alonso-Villaverde C, Coll B, Parra S, Montero M, Calvo N, Tous M*, et al.* **Atherosclerosis in patients infected with HIV is influenced by a mutant monocyte chemoattractant protein-1 allele**. *Circulation* 2004, **110**:2204-2209.

78. Varin A, Manna SK, Quivy V, Decrion AZ, Van Lint C, Herbein G*, et al.* **Exogenous Nef protein activates NF-kappa B, AP-1, and c-Jun N-terminal kinase and stimulates HIV transcription in promonocytic cells. Role in AIDS pathogenesis**. *J Biol Chem* 2003, **278**:2219-2227.

79. Bachelerie F, Alcami J, Arenzana-Seisdedos F, Virelizier JL. **HIV enhancer activity perpetuated by NF-kappa B induction on infection of monocytes.** *Nature* 1991, **350**:709-712.

80. Kopp E, Ghosh S. **Inhibition of NF-kappa B by sodium salicylate and aspirin.** *Science* 1994, **265**:956-959.

81. Pieper GM, Olds CL, Bub JD, Lindholm PF. **Transfection of human endothelial cells with HIV-1 tat gene activates NF-kappa B and enhances monocyte adhesion**. *American Journal of Physiology - Heart & Circulatory Physiology* 2002, **283**:H2315-2321.

82. Chirmule N, Kalyanaraman VS, Pahwa S. **Signals transduced through the CD4 molecule on T lymphocytes activate NF-kappa B**. *Biochem Biophys Res Commun* 1994, **203**:498-505.

83. Dhawan S, Puri RK, Kumar A, Duplan H, Masson JM, Aggarwal BB. **Human immunodeficiency virus-1-tat protein induces the cell surface expression of endothelial leukocyte adhesion molecule-1, vascular cell adhesion molecule-1, and intercellular adhesion molecule-1 in human endothelial cells**. *Blood* 1997, **90**:1535-1544.

84. Kuller L, Group SS. **Elevated Levels of Interleukin-6 and D-dimer Are Associated with an Increased Risk of Death in Patients with HIV**. *15th Conference on Retroviruses and Opportunistic Infections*. Boston, MA, Feb 3-6 2008.

85. de Larranaga GF, Bocassi AR, Puga LM, Alonso BS, Benetucci JA. **Endothelial markers and HIV infection in the era of highly active antiretroviral treatment**. *Thromb Res* 2003, **110**:93-98.

86. Henry K, Kitch D, Dube M, Zackin R, Parker RA, Sprecher D*, et al.* **C-Reactive protein levels over time and cardiovascular risk in HIV-infected individuals suppressed on an indinavir-based regimen: AIDS Clinical Trials Group 5056s**. *AIDS* 2004, **18**:2434-2437.

87. Autran B, Carcelain G, Li TS, Blanc C, Mathez D, Tubiana R*, et al.* **Positive effects of combined antiretroviral therapy on CD4+ T cell homeostasis and function in advanced HIV disease.** *Science* 1997, **277**:112-116.

88. Koblavi-Deme S, Maran M, Kabran N, Borget MY, Kalou M, Kestens L*, et al.* **Changes in levels of immune activation and reconstitution markers among HIV-1-infected Africans receiving antiretroviral therapy**. *AIDS* 2003, **17 Suppl 3**:S17-22.

89. Mattapallil JJ, Douek DC, Hill B, Nishimura Y, Martin M, Roederer M. **Massive infection and loss of memory CD4+ T cells in multiple tissues during acute SIV infection.** *Nature* 2005, **434**:1093-1097.

90. Brenchley JM, Schacker TW, Ruff LE, Price DA, Taylor JH, Beilman GJ*, et al.* **CD4+ T cell depletion during all stages of HIV disease occurs predominantly in the gastrointestinal tract**. *J Exp Med* 2004, **200**:749-759.

91. Guadalupe M, Reay E, Sankaran S, Prindiville T, Flamm J, McNeil A*, et al.* **Severe CD4+ T-cell depletion in gut lymphoid tissue during primary human immunodeficiency virus type 1 infection and substantial delay in restoration following highly active antiretroviral therapy**. *J Virol* 2003, **77**:11708-11717.

92. Guadalupe M, Sankaran S, George MD, Reay E, Verhoeven D, Shacklett BL*, et al.* **Viral suppression and immune restoration in the gastrointestinal mucosa of human immunodeficiency virus type 1-infected patients initiating therapy during primary or chronic infection**. *J Virol* 2006, **80**:8236-8247.

93. Brenchley JM, Price DA, Schacker TW, Asher TE, Silvestri G, Rao S*, et al.* **Microbial translocation is a cause of systemic immune activation in chronic HIV infection.** *Nat Med* 2006, **12**:1365-1371.

94. Giorgi JV, Hultin LE, McKeating JA, Johnson TD, Owens B, Jacobson LP*, et al.* **Shorter survival in advanced human immunodeficiency virus type 1 infection is more closely associated with T lymphocyte activation than with plasma virus burden or virus chemokine coreceptor usage**. *J Infect Dis* 1999, **179**:859-870.

95. Hazenberg MD, Otto SA, van Benthem BHB, Roos MTL, Coutinho RA, Lange JMA*, et al.* **Persistent immune activation in HIV-1 infection is associated with progression to AIDS**. *AIDS* 2003, **17**:1881-1888.

96. Hunt PW, Martin JN, Sinclair E, Bredt B, Hagos E, Lampiris H*, et al.* **T cell activation is associated with lower CD4+ T cell gains in human immunodeficiency virus-infected patients with sustained viral suppression during antiretroviral therapy**. *J Infect Dis* 2003, **187**:1534-1543.

97. Gupta SK, Johnson RM, Saha CK, Mather KJ, Greenwald ML, Waltz JS*, et al.* **Improvement in HIV-Related Endothelial Dysfunction Using the Anti-Inflammatory Agent Salsalate: A Pilot Study**. *AIDS* 2008, **22**:653-655.

98. Dubé MP, Shen C, Waltz JS, Greenwald ML, Mather K, Gupta SK. **Relationship of body composition, antiretroviral use, and HIV disease factors to endothelial dysfunction in HIV-infected subjects.** *Antiviral Therapy* 2007, **Supplement 2**:L15.

99. Kathiresan S, Gona P, Larson MG, Vita JA, Mitchell GF, Tofler GH*, et al.* **Cross-sectional relations of multiple biomarkers from distinct biological pathways to brachial artery endothelial function**. *Circulation* 2006, **113**:938-945.

100. Fazarinc F, Steen SN. **Long-term management of rheumatoid arthritis with disalcid**. *J Int Med Res* 1980, **8**:339-342.

101. Singleton PT, Jr. **Salsalate: its role in the management of rheumatic disease**. *Clin Ther* 1980, **3**:80-102.

102. McPherson TC. **Salsalate for arthritis: a clinical evaluation**. *Clin Ther* 1984, **6**:388-403.

103. April P, Abeles M, Baraf H, Cohen S, Curran N, Doucette M*, et al.* **Does the acetyl group of aspirin contribute to the antiinflammatory efficacy of salicylic acid in the treatment of rheumatoid arthritis?** *Semin Arthritis Rheum* 1990, **19**:20-28.

104. Yin MJ, Yamamoto Y, Gaynor RB. **The anti-inflammatory agents aspirin and salicylate inhibit the activity of I(kappa)B kinase-beta**. *Nature* 1998, **396**:77-80.

105. Mielants H, Veys EM, Verbruggen G, Schelstraete. **Comparison of serum salicylate levels and gastro-intestinal blood loss between salsalate (Disalcid)and other forms of salicylates**. *Scand J Rheumatol* 1981, **10**:169-173.

106. Morris HG, Sherman NA, McQuain C, Goldlust MB, Chang SF, Harrison LI. **Effects of salsalate (nonacetylated salicylate) and aspirin on serum prostaglandins in humans**. *Ther Drug Monit* 1985, **7**:435-438.

107. Estes D, Kaplan K. **Lack of platelet effect with the aspirin analog, salsalate**. *Arthritis Rheum* 1980, **23**:1303-1307.

108. Cryer B, Goldschmiedt M, Redfern JS, Feldman M. **Comparison of salsalate and aspirin on mucosal injury and gastroduodenal mucosal prostaglandins**. *Gastroenterology* 1990, **99**:1616-1621.

109. Scheiman JM, Elta GH. **Gastroduodenal mucosal damage with salsalate versus aspirin: results of experimental models and endoscopic studies in humans**. *Semin Arthritis Rheum* 1990, **20**:121-127.

110. Leonards JR. **Absence of gastrointestinal bleeding following administration of salicylsalicylic acid**. *J Lab Clin Med* 1969, **74**:911-914.

111. Cohen A. **Fecal blood loss and plasma salicylate study of salicylsalicylic acid and aspirin**. *J Clin Pharmacol* 1979, **19**:242-247.

112. Heinkelein M, Schneider-Schaulies J, Walker BD, Jassoy C. **Inhibition of cytotoxicity and cytokine release of CD8+ HIV-specific cytotoxic T lymphocytes by pentoxifylline**. *Journal of Acquired Immune Deficiency Syndromes & Human Retrovirology* 1995, **10**:417-424.

113. Wanchu A, Khullar M, Bhatnagar A, Sud A, Bambery P, Singh S. **Pentoxiphylline reduces nitric oxide production among patients with HIV infection**. *Immunol Lett* 2000, **74**:121-125.

114. Fazely F, Dezube BJ, Allen-Ryan J, Pardee AB, Ruprecht RM. **Pentoxifylline (Trental) decreases the replication of the human immunodeficiency virus type 1 in human peripheral blood mononuclear cells and in cultured T cells.** *Blood* 1991, **77**:1653-1656.

115. Biswas DK, Ahlers CM, Dezube BJ, Pardee AB. **Cooperative inhibition of NF-kappa B and Tat-induced superactivation of human immunodeficiency virus type 1 long terminal repeat**. *Proc Natl Acad Sci U S A* 1993, **90**:11044-11048.

116. Biswas DK, Ahlers CM, Dezube BJ, Pardee AB. **Pentoxifylline and other protein kinase C inhibitors down-regulate HIV-LTR NF-kappa B induced gene expression**. *Mol Med* 1994, **1**:31-43.

117. Dezube BJ, Lederman MM, Spritzler JG, Chapman B, Korvick JA, Flexner C*, et al.* **High-dose pentoxifylline in patients with AIDS: inhibition of tumor necrosis factor production. National Institute of Allergy and Infectious Diseases AIDS Clinical Trials Group**. *J Infect Dis* 1995, **171**:1628-1632.

118. Dezube BJ, Pardee AB, Chapman B, Beckett LA, Korvick JA, Novick WJ*, et al.* **Pentoxifylline decreases tumor necrosis factor expression and serum triglycerides in people with AIDS. NIAID AIDS Clinical Trials Group.** *J Acquir Immune Defic Syndr* 1993, **6**:787-794.

119. Fossat C, Fabre D, Alimi Y, Bienvenu J, Aillaud MF, Lenoble M*, et al.* **Leukocyte activation study during occlusive arterial disease of the lower limb: effect of pentoxifylline infusion**. *J Cardiovasc Pharmacol* 1995, **25 Suppl 2**:S96-100.

120. Boldt J, Brosch C, Lehmann A, Haisch G, Lang J, Isgro F. **Prophylactic use of pentoxifylline on inflammation in elderly cardiac surgery patients**. *Ann Thorac Surg* 2001, **71**:1524-1529.

121. Tsang GM, Allen S, Pagano D, Wong C, Graham TR, Bonser RS. **Pentoxifylline preloading reduces endothelial injury and permeability in cardiopulmonary bypass**. *ASAIO J* 1996, **42**:M429-434.

122. Sliwa K, Woodiwiss A, Kone VN, Candy G, Badenhorst D, Norton G*, et al.* **Therapy of ischemic cardiomyopathy with the immunomodulating agent pentoxifylline: results of a randomized study**. *Circulation* 2004, **109**:750-755.

123. Prasad K, Lee P. **Suppression of hypercholesterolemic atherosclerosis by pentoxifylline and its mechanism**. *Atherosclerosis* 2007, **192**:313-322.

124. Coe DA, Freischlag JA, Johnson D, Mudaliar JH, Kosciesza SA, Traul DK*, et al.* **Pentoxifylline prevents endothelial damage due to ischemia and reperfusion injury**. *J Surg Res* 1997, **67**:21-25.

125. Kim NY, Pae HO, Kim YC, Choi CK, Rim JS, Lee HS*, et al.* **Pentoxifylline potentiates nitric oxide production in interleukin-1beta-stimulated vascular smooth muscle cells through cyclic AMP-dependent protein kinase A pathway**. *Gen Pharmacol* 2000, **35**:205-211.

126. Berkenboom G, Fang ZY, Unger P, Goldman M, Fontaine J. **Endothelium-dependent effects of pentoxifylline in rat aorta**. *Eur J Pharmacol* 1991, **193**:81-86.

127. Bilsborough W, O'Driscoll G, Stanton K, Weerasooriya R, Dembo L, Taylor R*, et al.* **Effect of lowering tumour necrosis factor-alpha on vascular endothelial function in Type II diabetes**. *Clin Sci* 2002, **103**:163-169.

128. Gupta S, Johnson R, Saha C, Mather K, Waltz J, Greenwald M*, et al.* **A Pilot Study of the TNF-alpha Inhibitor Pentoxifylline to Improve HIV-Related Endothelial Dysfunction**. *15th Conference on Retroviruses and Opportunistic Infections (CROI 2008)*. Boston, MA, Feb 3-6 2008.

129. Grunfeld C, Pang M, Doerrler W, Shigenaga JK, Jensen P, Feingold KR. **Lipids, lipoproteins, triglyceride clearance, and cytokines in human immunodeficiency virus infection and the acquired immunodeficiency syndrome**. *J Clin Endocrinol Metab* 1992, **74**:1045-1052.

130. Corretti MC, Anderson TJ, Benjamin EJ, Celermajer D, Charbonneau F, Creager MA*, et al.* **Guidelines for the ultrasound assessment of endothelial-dependent flow-mediated vasodilation of the brachial artery: a report of the International Brachial Artery Reactivity Task Force.** *J Am Coll Cardiol* 2002, **39**:257-265.

131. Williams MR, Westerman RA, Kingwell BA, Paige J, Blombery PA, Sudhir K*, et al.* **Variations in endothelial function and arterial compliance during the menstrual cycle**. *J Clin Endocrinol Metab* 2001, **86**:5389-5395.

132. Torgrimson BN, Meendering JR, Kaplan PF, Minson CT. **Endothelial Function across an Oral Contraceptive Cycle in Women Using Levonorgestrel and Ethinyl Estradiol**. *Am J Physiol Heart Circ Physiol* 2007.

133. Sorensen KE, Celermajer DS, Spiegelhalter DJ, Georgakopoulos D, Robinson J, Thomas O*, et al.* **Non-invasive measurement of human endothelium dependent arterial responses: accuracy and reproducibility**. *Br Heart J* 1995, **74**:247-253.

134. Expert Committee on the Diagnosis and Classification of Diabetes M. **Report of the expert committee on the diagnosis and classification of diabetes mellitus**. *Diabetes Care* 2003, **26 Suppl 1**:S5-20.

135. Cockcroft DW, Gault MH. **Prediction of creatinine clearance from serum creatinine**. *Nephron* 1976, **16**:31-41.

136. Mather KJ, Hunt AE, Steinberg HO, Paradisi G, Hook G, Katz A*, et al.* **Repeatability characteristics of simple indices of insulin resistance: implications for research applications**. *J Clin Endocrinol Metab* 2001, **86**:5457-5464.

137. Matthews DR, Hosker JP, Rudenski AS, Naylor BA, Treacher DF, Turner RC. **Homeostasis model assessment: insulin resistance and beta-cell function from fasting plasma glucose and insulin concentrations in man**. *Diabetologia* 1985, **28**:412-419.

APPENDIX I: FORMULAS AND DEFINITIONS

1. Cockcroft-Gault Equation [135] for Estimated Creatinine Clearance (C_Cr_)

C-G C_Cr_ (mL/min) = [ (140-age) * (weight in kg) * (0.85 if female) / 72] / (S_Cr_ in mg/dL)

2. Blood Pressure Measurements: For statistical purposes, the value for the continuous variable of blood pressure will be the mean of the second and third measurements recorded.

3. Waist to Hip Ratio: For statistical purposes, the value for the continuous variable of waist circumference will be the mean of the three midwaist measurements. This value will then be divided by the mean of the three hip circumference measurements.

4. Quantitative Insulin Check Index (QUICKI) [136]

QUICKI = 1 / [ log_10_(mean glucose) + log_10_(mean insulin) ],

Where the mean fasting glucose (mg/dL) and insulin (mIU/mL) are those two sets of measurements obtained 10 minutes apart

5. Homeostasis Model Assessment of Insulin Resistance (HOMA-IR) [137]

HOMA_IR_ = mean fasting insulin (µIU/mL) / [22.5 * mean fasting glucose (mmol/L)

Where the mean fasting glucose (mg/dL) and insulin (mIU/mL) are those two sets of measurements obtained 10 minutes apart;

To convert the value for serum glucose in mg/dL to mmol/L, multiply by 0.05551

6. Body Mass Index (BMI)

BMI (kg/m^2^) = [weight (kg) / height^2^ (m)]

7. Body Surface Area (BSA)

BSA (m²) = { [height (cm) * weight (kg) ] / 3600}^½^

# Study No.: 0809-20

# INDIANA UNIVERSITY INFORMED CONSENT STATEMENT FOR

**A Randomized, Placebo-Controlled Trial of Pentoxifylline to Improve Endothelial function in HIV-Infected Patients Not Requiring Antiretroviral Therapy**

**Version 2.0**

You are being asked to take part in this research study because you are infected with the Human Immunodeficiency Virus (HIV), the virus that causes Acquired Immunodeficiency Syndrome (AIDS). This study is sponsored by the National Institutes of Health. The doctor in charge of this study is Dr. Samir K. Gupta. Before you decide if you want to be a part of this study, we want you to know about the study.

This is a consent form. It gives you information about this study. The study staff will talk with you about this information. You are free to ask questions about this study at any time. If you agree to take part in this study, you will be asked to sign this consent form. You will get a copy to keep.

**STUDY PURPOSE**

Blood vessels carry blood away from the heart to all parts of the body. These blood vessels are capable of relaxing and narrowing when needed to provide more blood to the body. That is, the inside of the vessel gets bigger and smaller depending on how well the vessel reacts to the need for more blood flow. The inability of the blood vessel to relax may increase the risk for heart disease and stroke. Inflammation (the way the body reacts to injury or infection) of the blood vessels may not allow them to relax as much as they should. Because HIV is associated with inflammation, it is possible that the blood vessels in people infected with HIV may not relax properly. Pentoxifylline is a medication that has been approved by the Food and Drug Administration to improve leg pain in patients with blood vessel blockages in the legs. It is also a strong blocker of inflammation. Previous research suggests that this medication may reduce inflammation in patients infected with HIV. We have recently showed in a small study that this medicine may also improve blood vessel function in HIV-infected patients. However, a larger study is needed to confirm that pentoxifylline truly works for patients with HIV infection. The purpose of this study is to see if pentoxifylline safely improves the relaxation of blood vessels in the arteries in the arm in people with HIV infection who are not taking HIV medications. Half of the study participants will receive pentoxifylline and the other will receive placebo (no active drug). If it appears that pentoxifylline might have a beneficial effect in this study when compared to placebo, then we will study this medicine in a larger study to determine if this drug prevents heart attacks and strokes.

**NUMBER OF PEOPLE TAKING PART IN THE STUDY:**

If you agree to participate, you will be one of approximately 26 participants here at the Indiana University Medical Center to enter the trial. If you agree to participate, you will be asked to stay in this study for approximately 11 weeks.

**PROCEDURES FOR THE STUDY:**

If you agree to be in the study, you will do the following things:

## Screening Visit

The purpose of the one screening visit is to see if you qualify for this study. This visit will take no longer than 60 minutes. No matter where you usually receive your HIV care (IU or Wishard), the screening visit will take place at the Indiana Clinical Research Center (ICRC). At this screening visit, your medical records will be reviewed, we will ask you about your past medical history and the medications that you take, and a short physical examination will be performed. Blood tests (up to one tablespoon) to check your HIV status, immune function (ability to fight infection), liver function, kidney function, and blood cell counts will be performed at this screening visit. If you are a woman who is able to become pregnant, you will also have a urine pregnancy test done. The test results will be given to you and must show that you are not pregnant to continue participation in the study. If you decide not to take part in this study, or if you do not meet the eligibility requirements, we will still use the results from the screening tests to see why patients were not eligible for the study.

## Main Study Visits

*Entry Visit (Day 0, Week 0)*

If you qualify for the main part of the study, we will ask you to return to the ICRC within three weeks of your screening visit. Your medical records will be reviewed more completely. We will ask you about your smoking habits and your family history of medical problems. Your blood pressure, pulse (heart rate), height, weight, temperature, and waist and hip sizes will also be measured.

You will need to fast (nothing to eat or drink, except for water) for at least 8 hours before you come for this study visit. You will also be asked not to use any tobacco products for 8 hours before the study visit. You may use water with your morning medications. But if you need to take medications with food, then bring these medications with you so you can take them after the visit is completed. For the time you will be on this part of the study, you are asked not to change the amount of fruits, juices, antioxidants, tea, and coffee that you consume. Antioxidants slow down changes in the body that occur during the development of diseases. Examples are vitamins A, C, and E. It is important that whatever amount you take, you do not drastically change that amount. If you are taking sildenafil (Viagra, Silagra, or other generic preparations), vardenafil (Levitra), or tadalafil (Cialis), you should not be using it for at least 72 hours (3 days) before coming to the clinic and should not take it for at least 72 hours (3 days) after the tests. For your own safety, it is very important that you answer truthfully.

We will then place and keep an IV (small plastic tube) into a vein in your arm to draw your blood to measure the amount of fat and sugar in the blood. With your written permission, the results of these fat and sugar level tests will be sent to your main doctor when available. We will also do blood tests to check your kidney function and liver function, and to check for low blood counts. We also check if you have hepatitis B or hepatitis C, which are viruses that can cause liver damage. The total amount of blood that will be drawn at each main study visit will be approximately 4 tablespoons. We will also check a urine sample for high levels of protein in the urine. Some of your blood and urine will be prepared for testing, stored (with no information that could identify you personally), and tested for other special chemicals and proteins at a later time. Some of these samples will be sent to a central storage facility (without any links to your identity) as part of a larger study sponsored by the National Institutes of Health. The remaining samples will be kept here at IU. The results of these special tests will not be made available to you as they are used for research purposes and not for HIV management. You will be asked to sign a separate consent statement for this aspect of the study.

The ultrasound portion of the main study visit will be performed at the ICRC after your blood and urine tests are completed. You will have the first part of the ultrasound test done on an artery in your right lower arm. The ultrasound, which is a standard medical test, is painless and involves bouncing sound beams off an artery in your arm and so it can be seen on a TV screen and measurements may be made. A blood pressure cuff will be applied to your lower arm and inflated for approximately 5 minutes. After release of the cuff, a repeat ultrasound test of the artery in your arm will be obtained. After a 15-minute rest period, you will be given one dose of nitroglycerin by placing it under your tongue. When you take nitroglycerin it relaxes the arteries in your body, including in your arms, and has been approved by the United States Food and Drug Administration for this use. After taking nitroglycerin, your blood pressure will be monitored to make sure that it does not drop too much, and you will have another ultrasound test. Your heart rhythm will be closely checked during all parts of the ultrasound test using an electronic detector that will be placed on your chest. Once the ultrasound tests are done and it is certain that your blood pressure and heart rhythm are stable, then the IV will be taken out. Both parts of the ultrasound test will take a total of 30 minutes of your time. Approximately 2 hours will be needed to complete all parts of this main study visit.

At this time, you will be randomly assigned (as if by the toss of a coin) to receive pentoxifylline or placebo. These pills will look the same, so neither you nor the research team will know which types of pills you have received. You will be asked to start taking one tablet (either pentoxifylline or placebo) three times per day, or one tablet with each meal. You will receive the pills from the Indiana University Investigational Drug Services pharmacy. These pills will be provided free of charge. You will receive enough to last you to the next study visit. If at any time you have questions or problems with this medication, please contact the study team immediately.

*Second (Week 4) and Third (Week 8) Main Study Visits*

You will then be asked to return to the ICRC in approximately 4 and 8 weeks for the follow-up visits. We will review your medical records again, ask how you are feeling in general, and ask if you think the study pills are causing any problems. We will also ask you to bring in your pill bottle so we can check how many you have taken since the last study visit. We will also ask you how many pills you have missed taking in the past 3 days. Your blood pressure, pulse, height, weight, and waist and hip sizes will also be measured again. After one 8-hour period of fasting, you will then be asked to repeat the same blood tests (approximately 4 tablespoons), urine tests, and ultrasound measurements. We will also check for levels of pentoxifylline in the blood. The results of the fat and sugar tests will be given to your main doctor when available, but the results of the special tests (for research-related chemicals and proteins) will not be made available. Each of these visits will take no more than 2 hours. The total volume of blood required for study completion (screening and main study visits) will be no more than 1 cup. After all of these tests are done at the Week 8 visit, your participation in this study will be complete.

If, for any reason, you cannot complete these follow-up visits, you will be asked to return to the ICRC as soon as you can to complete one visit earlier than scheduled. After an 8 hour fast, you will have the same blood and urine tests done the same way as the first main study visit. You will also be asked to complete the ultrasound test.

**RISKS OF TAKING PART IN THE STUDY:**

Risks of taking Pentoxifylline

There may be risks or serious and/or life threatening side effects when other medications are taken with pentoxifylline. For your safety, you must tell the study doctor or nurse about all medications you are taking before you start this study. You must also tell him or her before you start any new medications while on the study. In addition, you must tell the study doctor or nurse before enrolling in any other clinical trials while on this study.

In general, pentoxifylline is considered a safe type of medication to reduce inflammation. However, pentoxifylline may still have side effects, most of which stop when the medication is stopped. The list below includes the more serious or common side effects with a known possible relationship with pentoxifylline. Please note that the list does not include all the side effects seen with this drug. If you have questions concerning the additional side effects, please ask the study team.

The more common risks associated with pentoxifylline include:

- Flushing (redness and warmth feeling in the body)
- Rash
- Nausea
- Abdominal pain or heartburn
- Vomiting or diarrhea
- Chest pain or palpitations (heartbeat sensations that feel like your **heart** is pounding or racing) or heart rhythm abnormalities
- Dizziness
- Nervousness
- Blurred vision
- Low blood cell counts leading to weakness, bleeding, or lowered ability to fight infection
- Kidney problems
- Liver problems

If any of these problems occur while taking pentoxifylline, we will ask you to contact us immediately so that we can do the appropriate tests to see if the medication is responsible. We will also do these tests at the Week 4 and 8 visits. We may ask you to reduce the amount of pentoxifylline based on these tests. If the study team or your doctor believes that you should stop taking pentoxifylline, you will be told to do so immediately and your participation in this study will be completed.

Risks of not being on HIV medications

You can only participate in this study if your HIV doctor has decided that you do not need HIV medications for at least the next 3-4 months. Your CD4 cell count, a measure of your immune function, has to be in the range where experts believe HIV medications are not necessary. Therefore, the risk of not being on HIV medications during this study is quite low. If at any time during this study, however, the study team or your HIV doctor thinks that you need to have HIV medications started immediately, this will be done and you will no longer be a participant in this study.

Risks of the arm ultrasound test

The arm ultrasound test is a painless imaging test that has no short or long-term risks. The test usually causes a mild to moderate uncomfortable feeling in your arm and hand because of the blood pressure cuff that is applied to your arm tightly. This is relieved once the blood pressure cuff is released.

Risks of taking nitroglycerin

You should not take sildenafil (Viagra, Silagra, or other generic preparations), VARDENAFIL (LEVITRA), OR TADALAFIL (CIALIS) within 72 hours (3 days) before or after taking nitroglycerin. Taking these medications together can cause serious, life-threatening side effects.

Your blood pressure may also go lower after taking nitroglycerin. The typical symptoms of low blood pressure include nausea, vomiting, weakness, perspiration and collapse. If you experience low blood pressure after taking nitroglycerin, your legs may be put up on a few pillows. Usually this side effect lasts for no more than a few minutes and goes away on its own as the nitroglycerin wears off.

Rarely, your blood pressure may drop very low, or not return to normal fast enough. In these rare cases, fluids may need to be given through the IV in your arm to return your blood pressure to normal. If your blood pressure drops too low after a dose of nitroglycerin you will not be given nitroglycerin for the remaining arm ultrasound tests.

Nitroglycerin can also cause a severe headache following the test. If the headache lasts more than one hour, the usual dose of a common over-the-counter pain reliever medication, Tylenol, will be recommended.

Nitroglycerin may also cause a sensation of flushing of the skin. It may also cause a rash.

Risks for putting in an IV in the arm

Putting in an IV can be mildly painful, and is associated with a very low risk of bruising, fainting, or a skin infection.

### The risks of possible loss of confidentiality

We will not tell others other than your main doctor that you are taking part in this study. All your blood and urine samples will have a special code that will not be linked to your identity. The electronic copies of the ultrasound tests will not have your name on them. All test results will be locked in a cabinet and restricted.

Risks related to pregnancy or breastfeeding

If you are pregnant or breastfeeding, you will not be allowed to participate in this study, because pentoxifylline may be unsafe for unborn babies or to the breastfed baby. A pregnancy test will be done at the screening visit to make sure you are not pregnant. It is important for you to tell us if you think you may be pregnant any time during the study so that we can do the proper tests.

**BECAUSE OF THE RISK INVOLVED,** **If you are a woman who could become pregnant, you and your partner must use TWO (2) reliable Forms of birth controL that you discuss with the study team. You must continue to use birth control until 1 month after stopping PENTOXIFYLLINE. Acceptable methods of birth control include two of the following methods:**

- **condoms (with or without a gel that can kill sperm),**
- **a diaphragm or cervical cap (with or without a gel that can kill sperm),**
- **an intrauterine device (IUD),**
- **or hormonal-based birth control (“the pill”).**

**Condoms are recommended AS ONE OF THE METHODS, because their appropriate use is the only contraception method effective for preventing HIV transmission.**

**BENEFITS OF TAKING PART IN THE STUDY:**

You may receive no direct benefit from participating in this study. However, you may receive some short-term benefits if inflammation and blood vessel function improves while receiving pentoxifylline. Also, the results of blood and urine testing done as part of this study may contribute to treatment decisions made between you and your primary care provider. Information learned from this study may help others who have HIV.

**ALTERNATIVES TO TAKING PART IN THE STUDY:**

Instead of being in the study, you have the option not to participate.

**CONFIDENTIALITY:**

Efforts will be made to keep your personal information confidential. We cannot guarantee absolute confidentiality. Your personal information may be disclosed if required by law. Your identity will be held in confidence in reports in which the study may be published.

Organizations that may inspect and/or copy your research records for quality assurance and data analysis include groups such as the investigator and his research associates, the Indiana University Institutional Review Board or its designees, and state or federal agencies, specifically the Office for Human Research Protections (OHRP) and the National Institutes of Health (NIH).

**COSTS:**

The costs of all study-related tests and for the pentoxifylline will be provided by the study. However, taking part in this study may lead to added costs to you or your insurance company. Please ask about any expected added costs or insurance problems.

**PAYMENT:**

You will receive payment for taking part in this study: You will be given $10 for the screening visit, even if you are not found to be eligible for the main study. If you are eligible for the main study visits, you will receive $100 for completing each of the 3 main study visits (blood and urine tests and arm ultrasound measurements). The total amount of money that you can receive is $310. You will be paid following the completion of each study visit. A check will be sent to through the US Postal Services. Once a visit is completed, it can take up to 2 weeks for you to receive the check.

**COMPENSATION FOR INJURY:**

In the event of physical injury resulting from your participation in this research, necessary medical treatment will be provided to you and billed as part of your medical expenses. Costs not covered by your health care insurer will be your responsibility. Also, it is your responsibility to determine the extent of your health care coverage. There is no program in place for other monetary compensation for such injuries. However, you are not giving up any legal rights or benefits to which you are otherwise entitled.

**CONTACTS FOR QUESTIONS OR PROBLEMS:**

You are encouraged to ask questions any time during the study. In the event you experience a side effect or have further questions about the study, you may call the clinic at (317) 944-8456 or Dr. Samir K. Gupta at 317-274-7936. If you cannot reach the researcher during regular business hours (i.e. 8:00AM-5:00PM), please call the Indiana University Human Subjects Office at 317/278-3458 or 800/696-2949. On nights, weekends, or holidays, and as a 24-hour contact, you may reach the Infectious Disease physician on call at (317) 944-5000. For questions about your rights as a research participant or to discuss problems, complaints or concerns about a research study, or to obtain information, or offer input, contact the Indiana University Human Subjects Office at (317) 278-3458 or (800) 696-2949. For emergencies related to the use of pentoxifylline, you may contact the Indiana University Hospital Pharmacy Number at (317) 944-0362.

**VOLUNTARY NATURE OF STUDY:**

Taking part in this study is voluntary. You may choose not to take part or may leave the study at any time. Not taking part will not affect your HIV or general health care. Leaving the study will not result in any penalty or loss of benefits to which you are entitled.

You will be told about new information that may affect your health, welfare, or willingness to stay in the study.

The study doctor may need to take you off the study without your permission, if he feels that it is not in your best interest for you to participate.

**SUBJECT’S CONSENT:**

In consideration of all of the above, I give my consent to participate in this research study.

I will be given a copy of this informed consent statement to keep for my records.

SUBJECTS SIGNATURE: Date:

(must be dated by the subject)

SIGNATURE OF PERSON OBTAINING CONSENT: Date:

INDIANA UNIVERSITY INFORMED CONSENT STATEMENT FOR

**A Randomized, Placebo-Controlled Trial of Pentoxifylline to Improve Endothelial function in HIV-Infected Patients Not Requiring Antiretroviral Therapy**

**Version 2.0**

CONSENT FOR FUTURE USE AND STORAGE OF BLOOD SAMPLES

You are being asked to allow the researchers to store and to use some of the blood and urine obtained from you during the study named above. You may choose not to take part in this aspect of the main study and still participate in the main study. Your blood samples may be stored indefinitely in laboratories at the Indiana University Medical Center and at a central storage facility sponsored by the National Institutes of Health to find out more about how HIV causes disease and affects blood vessel function and also how pentoxifylline affects HIV and inflammation. These specimens will be labeled with a coded number that can be linked to you, but will not include your name. There will be no human genetic tests performed on your specimens. The study of human genetics looks at inherited traits or conditions that are passed at birth from parents to children.

If in the future, you decide that you do not want your specimens used for such research, please notify the Infectious Disease Research Clinic at (317) 944-8456 or in writing to Infectious Diseases Research Clinic, University Hospital, Room 5510, 550 N. University Boulevard, Indianapolis, IN 46202, and your specimens will be destroyed.

SUBJECT’S CONSENT:

In consideration of all of the above, I give my consent to have any of my samples obtained under the study named above to be stored and used for research related to HIV infection and its complications. This research will be done at a later date. I acknowledge receipt of a copy of this informed consent statement.

SUBJECTS SIGNATURE: Date:

(must be dated by the subject)

SIGNATURE OF PERSON OBTAINING CONSENT: Date:
